# Longitudinal antibody profiling after dengue reveals distinct dynamics by antibody specificity over 18 months

**DOI:** 10.1038/s41467-026-74406-0

**Published:** 2026-06-21

**Authors:** Sandra Bos, Tulika Singh, José Victor Zambrana, Elias Duarte, Reinaldo Mercado-Hernandez, Julia Huffaker, Aaron Graber, Angel Balmaseda, Eva Harris

**Affiliations:** 1 Division of Infectious Diseases and Vaccinology, School of Public Health, University of California, Berkeley, Berkeley, CA USA; 2 Department of Infectious Diseases and Microbiology, School of Public Health, University of Pittsburgh, Pittsburgh, PA USA; 3 Sustainable Sciences Institute, Oakland, CA USA; 4 Department of Epidemiology, School of Public Health, University of Michigan, Ann Arbor, MI USA; 5 Laboratorio Nacional de Virología, Centro Nacional de Diagnóstico y Referencia, Ministerio de Salud, Managua, Nicaragua; 6 Sustainable Sciences Institute, Managua, Nicaragua

**Keywords:** Antibodies, Dengue virus

## Abstract

The four dengue virus serotypes (DENV1-4) co-circulate worldwide, posing major challenges for vaccine development. One key issue is that certain levels and subsets of cross-reactive antibodies have been associated with enhanced disease during subsequent infection with a different DENV serotype. To understand the heterogeneity of DENV antibody responses and delineate their distinct kinetics, we define the magnitude and kinetics of 84 antiviral antibody subsets (by isotype, subclass, antigen, and cross-reactivity) after primary versus secondary dengue, using longitudinal samples collected <1, 3, 6 and 18 months post-symptom onset from a pediatric hospital study in Nicaragua. Interestingly, we find that after primary infection, cross-reactive IgG antibody responses against the envelope protein rise, not wane, over time. Antibody kinetics vary by specificity as measured by homologous versus cross-reactive subsets, viral antigen, and subdomain of a single antigen. Further, a substantial fraction of subjects still have IgA, IgM, and IgG3 responses above the assay background at 18 months post-infection. Overall, we find that the cross-reactive subset of post-primary anti-DENV antibody responses demonstrates distinct kinetics from overall DENV-binding antibodies as well as from secondary immune responses, which has implications for the outcome of subsequent DENV infections.

## Abstract (web summary)

Longitudinal antibody profiling in a Nicaraguan pediatric dengue cohort shows that cross-reactive IgG against the envelope protein rises, not wanes, over 18 months post-primary infection, with antibody kinetics differing by viral protein and domain.

## Introduction

Dengue virus serotypes 1–4 (DENV1–4) cause febrile illness with intense body pain, occasionally progressing to severe complications, including shock^1^. More than half the world’s population is at risk, with well over 100 million infections annually^2^. Further, DENV transmission zones are expanding with climate change beyond the tropics and sub-tropics^3^. Addressing dengue is urgent due to inconsistent mosquito control, the lack of DENV-specific therapies, and limited vaccine efficacy and availability^4^. Given the potential for antibody-mediated immunopathology^5^, understanding antibody responses to DENV infection is critical.

Antibodies can either protect against or enhance dengue disease^6–9^. Primary DENV infection is thought to induce lifelong immunity against the infecting serotype but generates cross-reactive (XR) IgG antibodies, which have the potential to exacerbate infection with a distinct serotype^5^. Among several mechanisms for antibody-mediated immunopathogenesis, the theory of antibody-dependent enhancement posits that antibodies facilitate viral entry into immune cells via Fcγ receptors, leading to increased viral replication, immune activation, and disease severity^10–15^. Related flaviviruses, such as Zika virus (ZIKV), also produce XR antibodies that can increase the risk of severe dengue during subsequent infections^16–20^. In contrast, post-secondary DENV infections generally confer broader, protective immunity through higher-quality XR antibodies, although the durability of these responses remains unclear^21–24^.

Current evidence supports only short-term (3–18 months) cross-protection after primary infection^25–29^. The classical hypothesis underlying severe secondary dengue posits that XR DENV antibodies wane to enhancing levels following primary infection but remain elevated and protective after secondary infection. However, recent studies have found stable long-term DENV-binding inhibition ELISA and neutralizing antibody levels post-primary infection and slow antibody waning post-secondary infection^19,30–32^. Thus, antibody waning versus stability post-primary infection remains controversial.

The antigenic relatedness between flaviviruses varies significantly across viral proteins due to differences in their conservation and structure^33^. The envelope (E) protein, forming dimers on the virus surface, elicits most antibodies, with dimer-specific neutralizing antibodies primarily targeting domains EDI and EDII, while the conserved fusion loop in EDII generates mostly poorly neutralizing antibodies^11,33–37^. Antibodies targeting the secreted non-structural protein 1 (NS1), although not directly neutralizing, may reduce disease severity, yet the long-term stability of antibodies against this protein remains poorly understood^38^.

Flavivirus immunity is influenced by IgG subclass composition (IgG1–4), which differ in FcγR-mediated immune functions, with IgG1 and IgG3 being pro-inflammatory, IgG2 arising in response to polysaccharide antigens, and IgG4 being immunoregulatory^39,40^. Minor serum isotypes, including IgM and IgA, also affect immunity, with IgM facilitating early neutralization and complement activation and IgA potentially modulating disease severity^41–44^. However, limited information exists regarding the long-term persistence of these lower-abundance antibodies.

In this study, we sought to understand the heterogeneity of DENV-binding antibody subsets and their distinct kinetics post-primary versus post-secondary dengue. We characterized the longitudinal dynamics of antibody responses by analyzing antibody isotypes, IgG subclasses, viral antigen specificity, and cross-reactivity profiles. Utilizing multiplex bead-based immunoassays on longitudinal serum samples ( < 1, 3, 6, and 18 months post-symptom onset) from pediatric dengue cases enrolled in a hospital-based study in Managua, Nicaragua, we investigated the kinetics and durability of these antibody populations. Our findings provide a comprehensive description of antibody subpopulations and their distinct kinetic patterns, offering new insights into dengue immunity relevant for vaccine development.

## Results

### Characteristics of participants

In this study, we analyzed plasma samples from 79 pediatric patients with either DENV1 or DENV3 infection. These participants were selected as a convenience sample from a hospital-based observational study in Managua, Nicaragua. We compared the plasma antibody profile of post-primary (1°; *n* = 39 after exclusion of one participant with a missing early convalescent sample) versus post-secondary (2°; *n* = 39) dengue groups. Longitudinal sampling was conducted at 2–4 weeks post-symptom onset (convalescence; Cv), as well as 3, 6, and 18 months (M) post-symptom onset (Supplementary Table S1).

### Definition of antibody compartments and analytical framework

To interrogate the composition and kinetics of anti-DENV antibody responses, we leveraged antigen selection to distinguish antibody compartments (Supplementary Fig. S1). For 1° dengue cases, antibodies measured against viral proteins from the infecting serotype (homologous antigens) represent a composite response that includes both type-specific antibodies unique to that serotype and cross-reactive antibodies targeting epitopes conserved across dengue viruses. In contrast, antibodies measured against proteins from non-infecting serotypes (heterologous antigens) must arise exclusively from cross-reactive antibodies, as individuals had no prior exposure to those serotypes. Additionally, for assessment of cross-reactive antibodies in both 1° and 2° cases, we considered that DENV4 did not circulate at epidemiologically relevant levels in Nicaragua from the early 1990s through the end of the study period (2013) and was not detected in 1° symptomatic infections. Therefore, DENV4 antigens were reliably used as the heterologous antigen to isolate cross-reactive antibody responses for both 1° and 2° dengue cases. We therefore use homologous antigens to measure the total (type-specific plus cross-reactive) antibody response, and heterologous DENV4 antigens to specifically capture the dynamics of cross-reactive antibodies.

Antibody kinetics were primarily inferred using linear mixed-effects models (LMMs) following the experimental and analytic approaches illustrated in Supplementary Fig. S2. Bayesian hierarchical modeling and non-parametric Wilcoxon analyses were applied as complementary approaches to assess robustness of observed trends. A summary of concordance across analytical frameworks for comparisons of infection groups is provided in Table S2 and discussed in Supplementary Note 1. Unless otherwise stated, biological interpretations in the main text are based on the primary LMM framework, with divergences across methods explicitly noted when present.

### Distinct antibody kinetics to homologous E protein following primary and secondary DENV infection

**Fig. 1:**
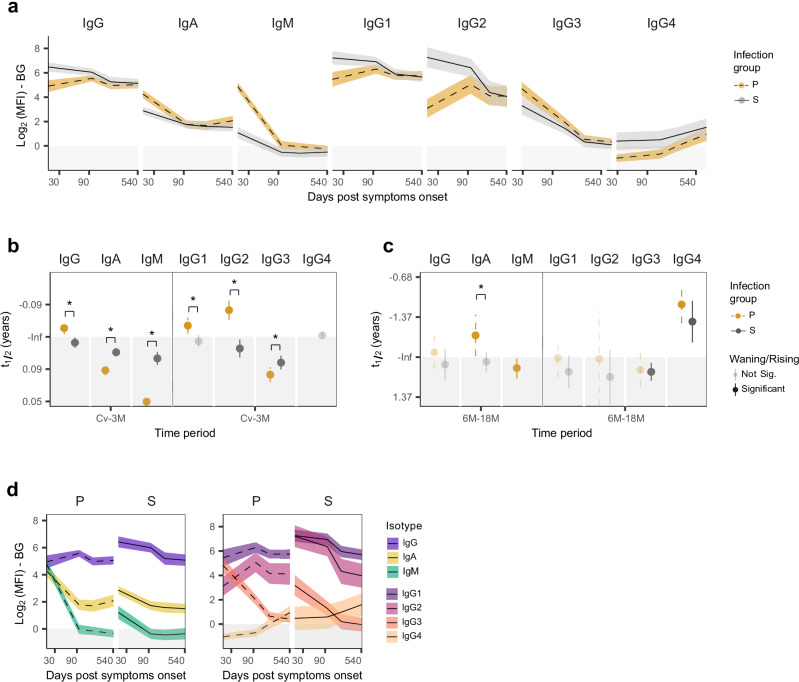
Antibody responses against homologous E protein after primary and secondary dengue. E-binding antibodies against the homologous infecting serotype were measured as Log_2_ of mean fluorescence intensity (MFI) after background subtraction (BG; shaded area). A linear mixed-effects model with bootstrapping was applied to estimate a linear rate of decay for three time-period segments: 30–90, >90–180, and >180–540 days post-symptom onset, corresponding to early convalescence to 3 months (Cv–3 M), 3–6 months (3–6 M), and 6–18 months (6–18 M) post-symptom onset (*n* = 39 post-primary; *n* = 39 post-secondary dengue cases). **a** Homologous E-binding antibody levels over time of primary (P; dotted line, yellow) versus secondary (S; solid line, grey) dengue cases, for each isotype and IgG subclass. Lines represent the model-estimated mean, and shaded bands indicate the 95% bootstrap confidence interval. Antibody levels are represented as the Log_2_ fold-change over background (Log_2_[MFI]–Log_2_[Background]). **b**, **c** Short-term antibody half-lives (*t*_1/2_) estimated from Cv–3 M antibody decay rates (**b**), and long-term *t*_1/2_ estimated from 6 to 18 M antibody decay rates (**c**). Points and error bars represent the bootstrap mean and 95% confidence interval. Transparent colors indicate stable decay rates with non-estimable infinite (−Inf) *t*_1/2_ (Not Sig.). Solid colors are significantly rising (−*t*_1/2_ above −Inf on *y*-axis) or waning (+*t*_1/2_ below −Inf on* y*-axis; shaded area) antibody kinetics. For time segments in which the modelled trajectory remained at or below zero over the entire interval (**a**), the corresponding half-life estimates and CI were omitted from (**b** and **c**). **d** Comparison of homologous E-binding antibody trajectories of primary (P; dotted line) versus secondary (S; solid line) dengue cases, with isotypes (IgG, IgA, IgM) and IgG subclasses (IgG1–4) overlaid. Lines represent the model-estimated mean, and shaded bands indicate the 95% bootstrap confidence interval; colors indicate antibody isotype/subclass (legend). Asterisks (*) denote significant differences between primary and secondary decay rates based on a confidence-interval overlap criterion. Source data are provided as a Source Data file.

To understand the generation and durability of the humoral immune response upon 1° versus 2° dengue, we first measured antibodies against the E protein of the infecting serotype (i.e., homologous) (Fig. 1 and Supplementary Figs. S3 and 4). We found that the initial magnitude of homologous E-reactive IgG (E-IgG) at Cv was higher post-2° than post-1° but decreased to a similar level by 18 M (Fig. 1a). The kinetics of initiating the E-IgG response differed in the Cv–3 M period: rising post-1° infection (Cv–3 M *t*_1/2_ = −0.34 years, 95% CI = −0.20, −1.11) and waning post-2° infection (Cv–3 M *t*_1/2_ = 0.51 years, 95% CI = 0.27, 4.37; Fig. 1a, b). This trend is driven by the IgG1 and IgG2 subclasses (Fig. 1d). In contrast, the initial magnitude of anti-E IgA, IgM, and IgG3 responses were higher post-1° than post-2° infection, reaching maximal observed levels at or before convalescence (Fig. 1a). Accordingly, the initial waning kinetic (Cv–3 M) for these subsets is steeper post-1° than post-2° infection (Fig. 1b, d). The initial levels and kinetics of E-IgG2 and E-IgM compartments demonstrate the greatest differences in 1° versus 2° immunity (Fig. 1a).

**Fig. 2:**
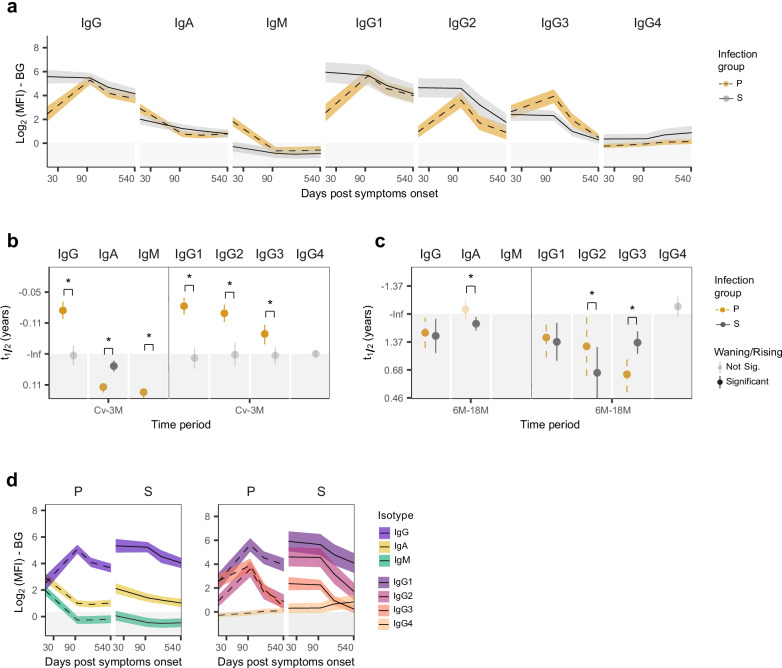
Dynamics of the antibody response against homologous DENV NS1 protein up to 18 months post-symptom onset. NS1-binding antibodies against the homologous infecting serotype (DENV1 or DENV3) were measured as Log_2_ of mean fluorescence intensity (MFI) after background subtraction (BG; shaded area). A linear mixed-effects model with bootstrapping was applied to estimate a linear rate of decay for three time-period segments: 30–90, >90–180, and >180–540 days post-symptom onset, corresponding to convalescent to 3-month (Cv–3 M), 3–6 months (3–6 M), and 6–18 months (6–18 M) post-symptom onset (*n* = 39 post-primary; *n* = 39 post-secondary dengue cases). **a** Homologous DENV NS1-binding antibody levels over time of primary (P; dotted line, yellow) versus secondary (S; solid line, grey) dengue cases, for each isotype and IgG subclass. Lines represent the model-estimated mean, and shaded bands indicate the 95% bootstrap confidence interval. Antibody levels are represented as Log_2_ fold-change over background (Log_2_[MFI]–Log_2_[Background]). **b**, **c** Short-term antibody half-lives (*t*_1/2_) estimated from Cv–3 M antibody decay rates (**b**), and long-term *t*_1/2_ estimated from 6 to 18 M antibody decay rates (**c**). Points and error bars represent the bootstrap mean and 95% confidence interval. Transparent colors indicate stable decay rates (Not Sig.); solid colors are significantly rising or waning antibody kinetics. For time segments in which the modelled trajectory remained at or below zero over the entire interval (**a**), the corresponding half-life estimates and CI were omitted from (**b** and **c**). **d** Comparison of homologous NS1-binding antibody trajectories of primary (P; dotted line) versus secondary (S; solid line) dengue cases, with isotypes (IgG, IgA, IgM) and IgG subclasses (IgG1–4) overlaid. Lines represent the model-estimated mean, and shaded bands indicate the 95% bootstrap confidence interval; colors indicate antibody isotype/subclass (legend). Asterisks (*) denote significant differences between primary and secondary decay rates based on a confidence-interval overlap criterion. Source data are provided as a Source Data file.

We then evaluated antibody durability from 6 to 18 M. E-IgG was stable in both 1° and 2° infections, as supported by the IgG1 and IgG2 compartments (Fig. 1c). Surprisingly, E-IgA increased after 1° (6–18 M *t*_1/2_ = −2.52 years, 95% CI = −1.29, −54.82) but not after 2° infections (6–18 M *t*_1/2_ = 11.78 years, 95% CI = 3.76, −10.37; Fig. 1c), a pattern supported by both modeling approaches but not by Wilcoxon testing (Supplementary Fig. S3 and Table S2). Intriguingly, E-IgG4 continued to rise long-term both after 1° (6–18 M *t*_1/2_ = −1.04 years, 95% CI = −0.77, −1.62; Fig. 1c, d) and 2° infections (6–18 M *t*_1/2_ = −1.54 years, 95% CI = −0.97, −3.75; Fig. 1d). Also, we found that by 18 M, the magnitude of each anti-E binding antibody subset was similar across both post-1° and post-2° dengue groups (Fig. 1a).

Although the anti-E antibody types generally followed the compositional hierarchy observed in healthy human blood (IgG > IgA > IgM, and IgG1 > IgG2 > IgG3 > IgG4), there were some exceptions^45^. In the Cv–3 M phase of the 1° immune response, IgM, IgG1, and IgG3 responses were equivalent (Fig. 1d). In the Cv–3 M phase of the 2° immune response, IgG2 and IgG1 responses were similar (Fig. 1d). In the durable post-2° IgG compartment, E-IgG4 exceeded E-IgG3 levels (Fig. 1d).

### Antibody responses against homologous NS1 demonstrate long-term waning

We next assessed how NS1-reactive antibody subsets differ from anti-E antibodies in post-1° versus post-2° groups by measuring the magnitude of antibodies against homologous NS1 (Fig. 2 and Supplementary Fig. S5). Many aspects were comparable, such as higher levels of NS1-IgG in early 2° compared to 1° responses, a more gradual rise toward maximal recorded levels of NS1-IgG in 1° compared to 2° responses, and a similar directionality of NS1-IgA and NS1-IgM in both 1° and 2° responses (Fig. 2a). Nonetheless, some distinct patterns emerged. The initial (Cv–3 M) rise of NS1-IgG in 1° infections was steeper than the Cv–3 M rise in post-1° E-IgG (Fig. 2a, b). In 2° infections, NS1-IgG and its IgG1 and IgG2 subclasses remained elevated at maximal observed levels through 3 M, whereas anti-E IgG declined in this period (Fig. 2a, d). Most different was NS1-IgG3, which rose in the 1° Cv–3 M period and was stable in 2° Cv–3 M, whereas E-IgG3 waned (Fig. 2a, b), suggesting a later initiation of NS1 responses than E responses, although 1° and 2° differences for this subclass were supported by both modeling approaches but not by Wilcoxon testing (Supplementary Fig. S5 and Table S2). In the 6–18 M period, anti-NS1 IgG, IgG1, IgG2, and IgG3 waned substantially, whereas the anti-E compartments were largely stable (Fig. 2c, d). Unlike with E-IgA in the 6–18 M phase, NS1-IgA antibodies were stable post-1° and waned substantially post-2° (Fig. 2c). At 18 M, post-2° anti-NS1 IgG2 and IgG4 levels were higher than post-1° levels (Fig. 2a, d). Thus, long-lasting NS1-IgG wanes, unlike E-IgG, and NS1-IgA demonstrates distinct trends in 1° versus 2° immunity.

### DENV serocomplex E-binding XR antibodies rise post-primary dengue

**Fig. 3:**
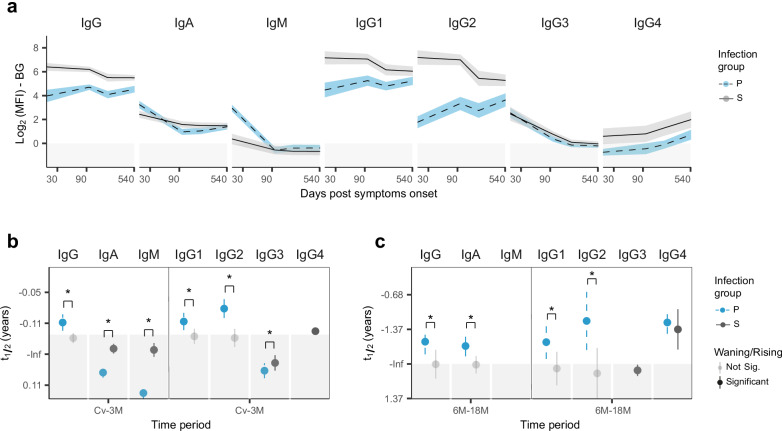
DENV serocomplex cross-reactive anti-E antibodies rise after primary dengue but are stable post-secondary dengue. Cross-reactive (XR) E-binding antibodies were assessed as Log_2_ of mean fluorescence intensity (MFI) after background subtraction (BG; shaded area), against the DENV4 serotype, which was infrequent in our cohort during the time-period of infections sampled here. A linear mixed-effects model with bootstrapping was applied to estimate a linear rate of decay for three time-period segments (Cv–3, 3–6, 6–18 M; see “Methods”) (*n* = 39 post-primary; *n* = 39 post-secondary dengue cases). **a** XR E-binding antibody levels over time of primary (P; dotted line, blue) versus secondary (S; solid line, grey) dengue cases, for each isotype and IgG subclass. Lines represent the model-estimated mean, and shaded bands indicate the 95% bootstrap confidence interval. Antibody levels are represented as Log_2_ fold-change over background (Log_2_[MFI]–Log_2_[Background]). **b**, **c** Short-term antibody half-lives (*t*_1/2_) estimated from Cv–3 M antibody decay rates (**b**), and long-term *t*_1/2_ estimated from 6 to 18 M antibody decay rates (**c**). Points and error bars represent the bootstrap mean and 95% confidence interval. Transparent colors indicate stable decay rates (Not Sig.); solid colors are significantly rising or waning antibody kinetics. For time segments in which the modelled trajectory remained at or below zero over the entire interval (**a**), the corresponding half-life estimates and CI were omitted from (**b** and **c**). Asterisks (*) denote significant differences between primary and secondary decay rates based on a confidence-interval overlap criterion. Source data are provided as a Source Data file.

Since DENV E-binding XR antibodies can be associated with subsequent immunopathology, we evaluated if this subset demonstrates distinct kinetics. We tested the differences in DENV4 XR antibody kinetics in post-1° versus post-2° dengue groups (Fig. 3 and Supplementary Figs. S6 and S7). While the early (Cv–3 M) kinetics of XR E-IgG were comparable to homologous E-IgG, the divergence in magnitude of 1° versus 2° IgG responses was more pronounced, particularly in the XR E-IgG1 and E-IgG2 compartment (Fig. 3a, b). Post-2° XR anti-E IgG, IgG1 and IgG2 started higher and remained higher (through 18 M) than post-1° responses, supporting more efficient selection of a XR antibody repertoire in 2° infections (Fig. 3a). While XR IgA and IgM anti-E antibodies were higher post-1° than post-2° at convalescence, this may be due to XR epitopes being occupied by higher affinity IgG in 2° infections (Fig. 3a).

The most notable differences between antibody responses to homologous versus DENV4-XR E were observed in the post-1° 6–18 M phase. IgG, IgG1, and IgG2 against XR E rose significantly after 1° infection (6–18 M *t*_1/2_ = −2.13 years, 95% CI = −1.36, −4.91), whereas antibodies against homologous E remained stable and did not rise (Fig. 3c and Supplementary Fig. S8A). The rising dynamics of XR IgG1 and IgG2 after 1° infection were also supported by Bayesian hierarchical modeling, and differences in dynamics between infection groups for these subclasses were consistently detected across all analytical frameworks (Supplementary Table 2). Also, after 2° infections, IgG, IgG1, and IgG2 against cross-reactive and homologous E were both stable (*t*_1/2_ = 140.35 years, 95% CI = −3.36, 3.21; Fig. 3c). Thus, increasing XR IgG after 1° infection contrasts with stable XR IgG post 2° infection. No waning was observed.

### Low and waning NS1 XR antibody responses

Next, we examined XR NS1 responses, as these antibodies may also contribute to subsequent protection or pathology. As with XR E antibodies, we quantified XR NS1 antibodies using DENV4-NS1, comparing 1° and 2° DENV infection groups. However, as cross-reactivity to DENV4-NS1 was often minimal or even undetectable following 1° infection, cross-reactivity to DENV2-NS1 was also assessed for this group (Supplementary Figs. S9 and 10). We found that the level of XR NS1 antibodies was much lower than the total response to NS1 in both 1° and 2° infection groups, indicating that only a small portion of the anti-NS1 response was XR (Supplementary Fig. S10A, D). In the 1° infection group at the 6–18 M phase, homologous NS1-IgG waned, but XR NS1-IgG was stable though low (Fig. 2 and Supplementary Fig. S10A, C). In the 2° infection group at the Cv–3 M phase, the homologous NS1-IgG response was stable, whereas the XR NS1-IgG response waned (Fig. 2 and Supplementary Fig. S10A, B, D). At 6–18 M phase after 2° dengue, both homologous and XR NS1-IgG and NS1-IgA waned significantly (Supplementary Fig. S10C, D). Thus, total versus XR NS1-IgG responses demonstrate distinct antibody kinetics.

### Different antibody dynamics by viral antigen and subdomain of the E protein

**Fig. 4:**
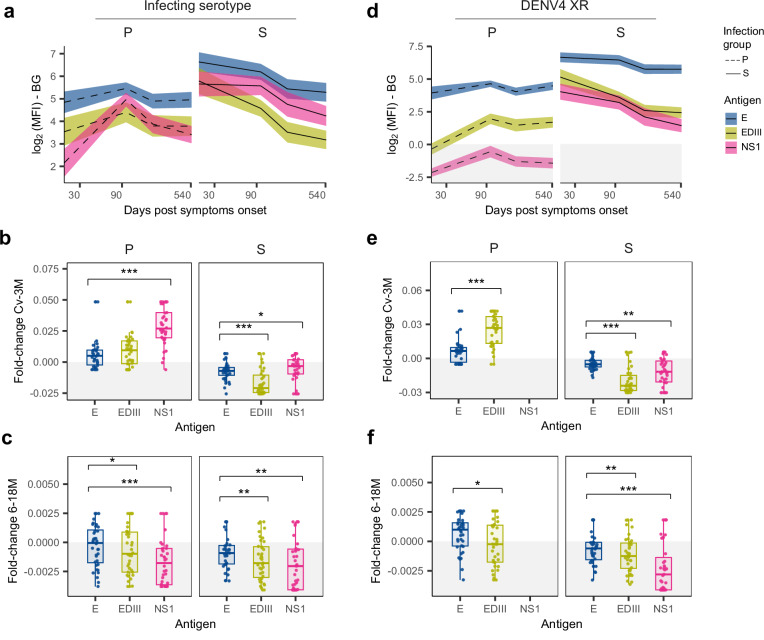
Distinct dynamics of antibodies against E, EDIII, and NS1 proteins. DENV-binding IgG levels were measured as Log_2_ of mean fluorescence intensity (MFI) after background subtraction (BG; shaded area). A linear mixed-effects model with bootstrapping was applied to estimate a linear rate of decay for three time-period segments (Cv–3, 3–6, 6–18 M; see “Methods”) (*n* = 39 post-primary; *n* = 39 post-secondary dengue cases). **a**, **d** Comparison of homologous (**a**) and DENV4 (**d**) E (blue), EDIII (lime green), and NS1 (pink) binding IgG levels over time in the primary (P; dotted line) versus secondary (S; solid line) dengue groups. Lines represent the model-estimated mean, and shaded bands indicate the 95% bootstrap confidence interval. Antibody levels are represented as Log_2_ fold-change over background (Log_2_[MFI]–Log_2_[Background]). **b**, **c** Annualized fold-change in binding IgG levels against E, EDIII, and NS1 from the infecting serotype between Cv–3 M (**b**) and 6–18 M (**c**). **e**, **f** Annualized fold-change in binding IgG levels against DENV4 E, EDIII, and NS1 between Cv–3 M (**e**) and 6–18 M (**f**). In (**b**, **c**, **e**, **f**), box plots show the median (center line), 25th–75th percentile (box), and 1.5 × IQR (whiskers); each dot represents one participant. For each infection group, fold-change in EDIII and NS1 responses was compared with fold-change in the corresponding E response using paired, two-sided, Bonferroni-corrected Wilcoxon signed-rank tests. Asterisks denote significant differences derived from adjusted *p*-values (*p < 0.05; **p < 0.01; ***p < 0.001); exact adjusted *p*-values are provided in the Source Data file. When the modelled antigen trajectory in (**a**) remained at or below zero over the entire time segment, the corresponding fold-change values were omitted from (**b**, **c**, **e**, and **f**). Source data are provided as a Source Data file.

We next examined whether IgG dynamics differed by viral antigen by comparing responses to EDIII (Supplementary Figs. S11–S14) and NS1 relative to the corresponding E response within individuals using paired Wilcoxon tests (Fig. 4, Source Data). This approach allowed us to assess differences in antibody initiation and durability across structural and non-structural antigens, as well as among antibodies targeting distinct regions within the E protein.

We first compared IgG dynamics against the E versus NS1 protein. Within the response against the infecting serotype, in the post-1° group, NS1-IgG exhibited a larger positive fold-change than E-IgG during the early Cv–3 M phase (Fig. 4b), indicating a delayed initiation of NS1- relative to E-IgG responses. In contrast, during the 6–18 M phase, NS1-IgG showed a more negative fold-change than E-IgG (Fig. 4c), consistent with greater long-term waning of NS1-IgG. Assessment of the corresponding pattern within the DENV4 XR IgG subset in post-1° was not possible, as XR NS1-IgG were below the detection threshold. In post-2° groups, XR NS1-IgG declined more rapidly than XR E-IgG during both the Cv–3 M and 6–18 M phases (Fig. 4e, f), indicating distinct durability of XR antibody responses targeting structural versus non-structural dengue antigens.

We next compared IgG dynamics against the E versus EDIII protein. Within the response to the infecting serotype (Fig. 4a–c), EDIII- and E-IgG exhibited similar early fold-changes from Cv–3 M in post-1° (Fig. 4b). However, during the late phase (6–18 M), EDIII-IgG showed a more negative fold-change than E-IgG (Fig. 4c), indicating greater waning of antibodies targeting domain III relative to the overall anti-E response. In contrast, XR EDIII-IgG displayed a larger positive fold-change than XR E-IgG during the Cv–3 M phase (Fig. 4e), indicating a delayed initiation of XR EDIII-IgG relative to XR E-IgG response. Interestingly, during the 6–18 M phase, XR E-IgG showed a more positive fold-change than XR EDIII-binding IgG (Fig. 4f), suggesting that the peculiar post-1° rise of IgG is primarily attributable to antibodies targeting E domains outside domain III. In the post-2° infection group, divergence between E- and EDIII-IgG was already evident during the Cv–3 M phase for both homologous and DENV4 XR responses, with EDIII-IgG exhibiting a more negative fold-change than E-IgG (Fig. 4b, e). These differences persisted from 6 to 18 months (Fig. 4c, f), indicating consistent, more pronounced waning of EDIII-IgG compared to antibodies recognizing the full E protein. Thus, IgG antibodies targeting domain III of the E protein can exhibit distinct initiation and durable kinetics compared to antibodies recognizing the full E protein.

### Distinct kinetics of total versus flavivirus-cross-reactive IgG subsets post-primary dengue

**Fig. 5:**
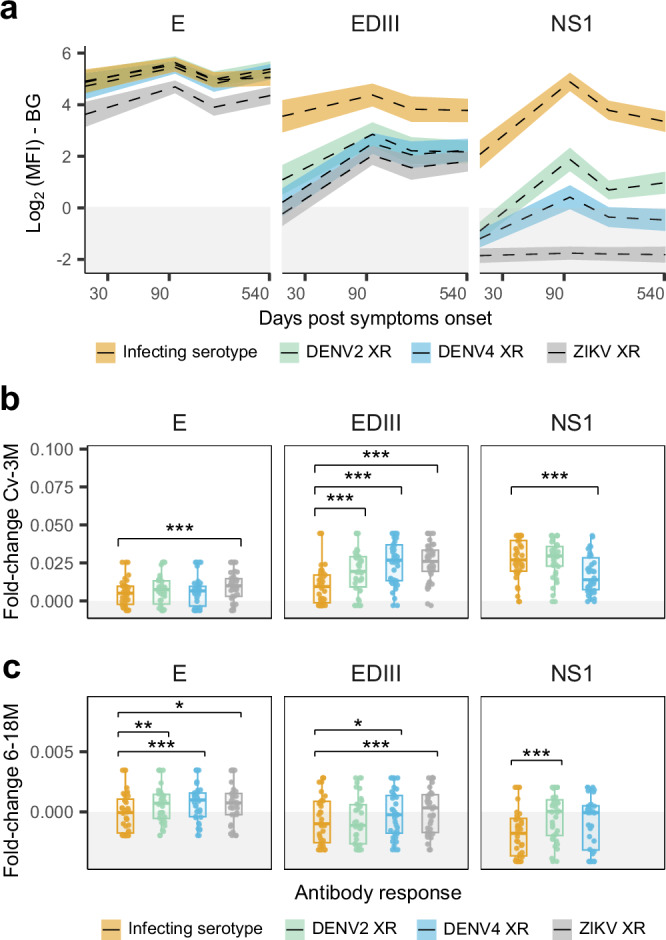
Antigenic hierarchy in the levels and kinetics of cross-reactive antibody responses post-primary dengue. The magnitude of flavivirus E-, EDIII-, and NS1-binding IgG was measured as Log_2_ of mean fluorescence intensity (MFI). A linear mixed-effects model with bootstrapping was applied to estimate a linear rate of decay for three time-period segments (Cv–3, 3–6, 6–18 M; see “Methods”) (*n* = 39 post-primary dengue cases). **a** In the primary dengue group (dotted line), total DENV-reactive IgG against the infecting serotype of DENV1 or DENV3 (yellow) was compared to the magnitude of DENV2 cross-reactive (DENV2 XR; green), DENV4 cross-reactive (DENV4 XR; blue), and ZIKV cross-reactive (ZIKV XR; grey) antibodies over time. Lines represent the model-estimated mean, and shaded bands indicate the 95% bootstrap confidence interval. Antibody levels are represented as Log_2_ fold-change over background (Log_2_[MFI]–Log_2_[Background]). **b**, **c** Annualized fold-change in binding IgG levels for each antibody response between Cv–3 M (**b**) and 6–18 M (**c**). In (**b** and **c**), box plots show the median (center line), 25th–75th percentile (box), and 1.5 × IQR (whiskers); each dot represents one participant. For each antigen, fold-change in cross-reactive responses was compared to the infecting serotype response using paired, two-sided, Bonferroni-corrected Wilcoxon signed-rank tests. Asterisks denote significant differences derived from adjusted *p*-values (*p < 0.05; **p < 0.01; ***p < 0.001); exact adjusted *p*-values are provided in the Source Data file. When the modelled trajectory in (**a**) remained at or below zero over the entire time segment, the corresponding fold-change values were omitted from (**b** and **c**). Source data are provided as a Source Data file.

Given that XR antibodies from a 1° immune response pose a risk for development of severe 2° dengue, we directly compared differences in the post-1° response against the infecting serotype (i.e., total) versus XR subsets (Fig. 5, Source Data, Supplementary Fig. S1). We defined three XR subsets: two within the DENV1–4 serocomplex (DENV2-XR and DENV4-XR) and another more antigenically distant, outside the DENV1–4 serocomplex (ZIKV-XR; Fig. 5a and Supplementary Fig. S15). Across antigens, IgG response against the infecting serotype was generally higher in magnitude than XR IgG responses against DENV4 or ZIKV, although this difference was least pronounced for E compared to EDIII and NS1 (Fig. 5a).

During the early Cv–3 M phase, E-IgG responses exhibited similar fold-changes across total versus DENV XR subsets (Fig. 5b). In contrast, DENV XR EDIII-IgG subsets showed larger positive fold-changes than the total EDIII response, suggesting a delayed establishment of DENV XR EDIII-IgG (Fig. 5a). Importantly, the 1° immune response was against DENV1 or DENV3 infections. Within the DENV1–4 serocomplex, DENV2 is the most antigenically related to these infecting serotypes, while DENV4 is the most distant. ZIKV, as part of another serocomplex, is antigenically the most divergent among the XR targets analyzed. In alignment with these antigenic relationships, EDIII- and NS1-IgG responses showed stepwise decreases in magnitude with increasing antigenic distance, whereas E-IgG responses did not separate from those against ZIKV (Fig. 5a).

Unexpectedly, all long-term (6–18 M) post-1° DENV and ZIKV XR E-IgG subsets showed a significant positive fold-change, indicating a rising antibody level, while the total E-IgG against the infecting serotype remained stable (Fig. 5c). In contrast, over the same period, NS1-IgG against the infecting serotype showed significant negative fold-change compared to DENV2 XR NS1-IgG which remained low and stable (Fig. 5b). Thus, total versus DENV XR IgG subsets showed distinct kinetics, with XR responses rising (E) or stable (NS1) over time.

### Seropositivity at 18 months post-infection reveals isotype- and antigen-specific patterns

**Fig. 6:**
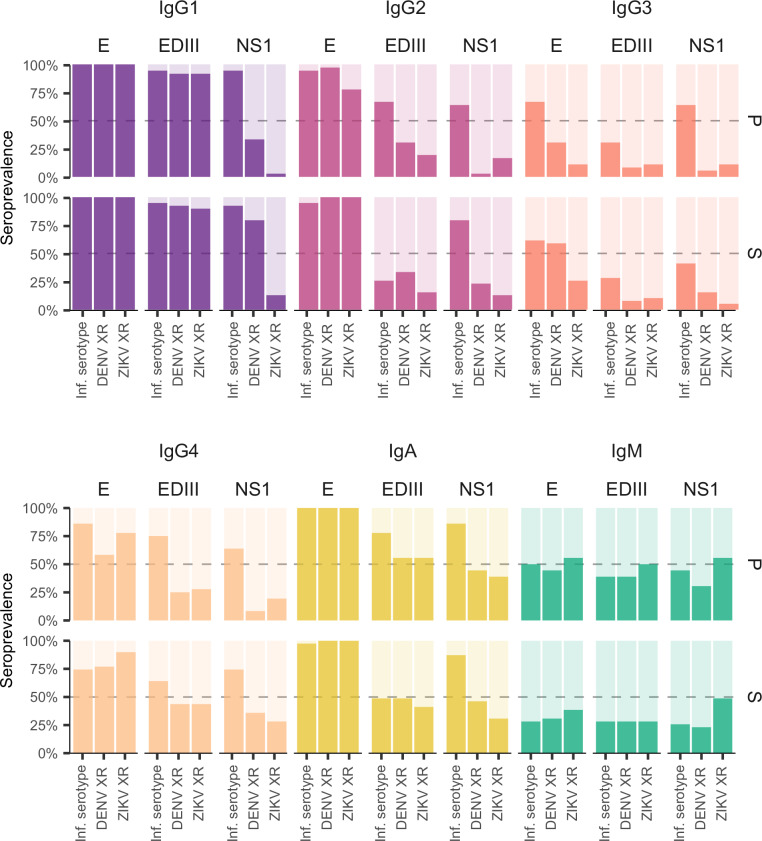
Seropositivity of antibody subsets at 18 months in post-primary versus post-secondary dengue groups. The proportion of the post-primary (P) and post-secondary (S) dengue groups with antibody levels at 18 months post-symptom onset above background was defined as percent seroprevalence for each group. In both groups, antibody subsets are shown by isotypes and IgG subclasses (colors) and stratified by viral antigen (E, EDIII, and NS1) and virus specificity (infecting serotype [Inf. Serotype], DENV4 cross-reactive [DENV XR], and ZIKV cross-reactive [ZIKV XR]). The dotted line marks 50% seroprevalence. Bars represent the percentage of participants seropositive in each group, calculated from observed 18-month values (*n* = 36 post-primary; *n* = 39 post-secondary dengue cases); individual data points are not overlaid because each bar is a binary-derived group-level proportion. Source data are provided as a Source Data file.

We evaluated the seropositivity of each DENV-binding antibody subset at 18 M after 1° versus 2° infections. Seropositivity was defined as the portion of each 1° or 2° infection group with an antibody response higher than the level of background. Surprisingly, even though IgA and IgM are considered short-lived, we found high seroprevalence of IgA and IgM antibodies against the E protein of the infecting serotype post-1° (IgA = 100%; IgM = 50%) and post-2° (IgA = 97%; IgM = 28%; Fig. 6) dengue. IgG1, IgG2, and IgA exhibited the highest seropositivity rates ( > 50%) for multiple specificities, but with greater seropositivity against viral structural proteins (E and EDIII) than NS1 (Fig. 6). Also, IgA, IgG2, and IgG3 demonstrated higher seropositivity for E than for the EDIII domain (Fig. 6 and Supplementary Fig. S8B). Moreover, seropositivity declined with greater antigenic distance from the infecting DENV serotype for many antibody subsets (NS1-IgG, NS1-IgA, EDIII-IgG2, E-IgG3), with highest responses towards the infecting serotype, followed by DENV4-XR, and then ZIKV-XR (Fig. 6). Taken together, these data indicate distinct antigenic biases by antibody type.

We then assessed which antibody subsets accumulate with exposure. Notably, seropositivity of XR responses was higher in 2° compared to 1° infections (Fig. 6 and Supplementary Fig. S16E). As expected, some antibody types (NS1-IgG1, E-IgG2, E-IgG3, and XR IgG4) showed higher seropositivity in 2° than 1° infection groups (Fig. 6 and Supplementary Fig. S8B). E-IgG1, and E-IgA did not differ in seropositivity, as they were maximal post-1° response, and IgG3 was low both post-1° and post-2° infections (Fig. 6). Other antibody subsets, including all IgM subsets, EDIII-IgA, and EDIII-IgG2 responses, were lower post-2° than post-1° infections, likely due to class-switching and distinct selection of 2° versus 1° immunity in these compartments (Fig. 6). In general, the seropositivity of XR responses accumulates with exposure, whereas EDIII seropositivity declines with exposure.

### Inter- and intra-individual variability in DENV antibody responses

**Fig. 7:**
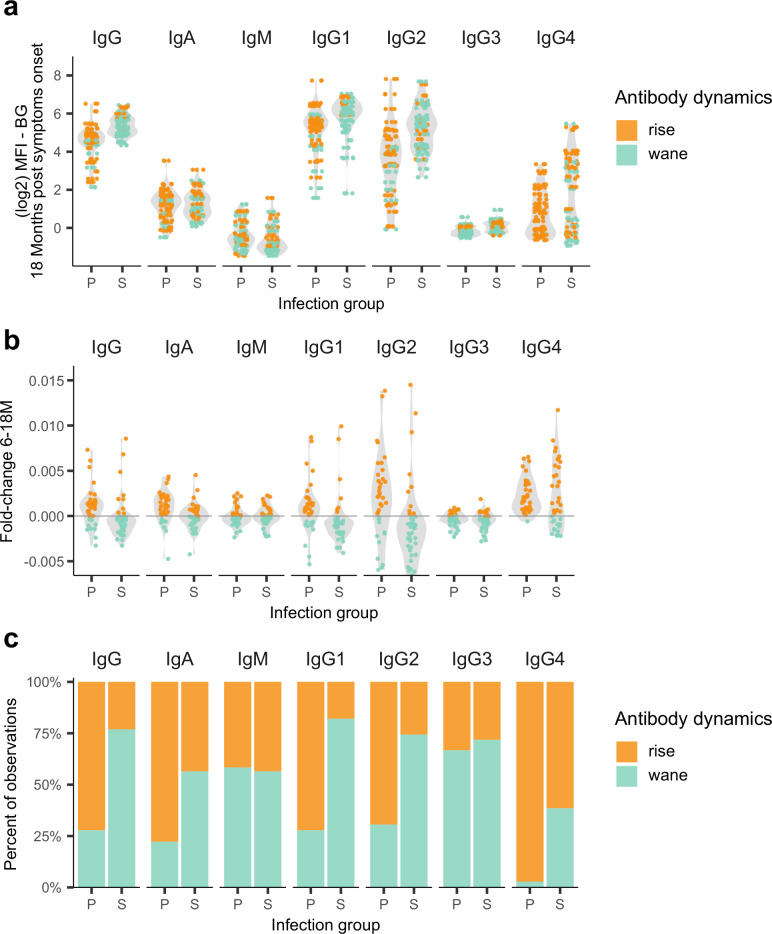
Interindividual variability in DENV4 envelope cross-reactive antibody magnitude and 6-to-18-month post-infection antibody kinetics. DENV4 E-binding antibodies were measured as Log_2_ of mean fluorescence intensity (MFI) after background subtraction (BG; shaded area) in both the post-primary (P) and post-secondary (S) dengue groups. **a** Distribution of the cross-reactive (XR) anti-E antibody magnitude across individuals at 18 months post-infection by antibody subgroup and primary versus secondary infection (*n* = 36 post-primary; *n* = 39 post-secondary). **b** Distribution of annualized fold-change in XR anti-E antibody binding between 6 and 18 M across individuals by antibody subgroup and primary versus secondary infection. Violin plots show the kernel density of antibody magnitudes (**a**) and annualized fold-change values (**b**); each dot represents one participant, colored by 6–18 M antibody kinetics (rising = orange; waning = green). **c** Proportion of the post-primary versus post-secondary infection group with either rising (orange) or waning (green) XR anti-E antibody kinetics from 6 to 18 M for each antibody isotype and IgG subclass. Source data are provided as a Source Data file.

Given that inter- and intra-individual differences in immune responses are indicative of host-specific immune set-points^46^, we sought to quantify this heterogeneity by antibody subsets. With regards to the post-1° 18-month DENV4 XR anti-E antibody levels, we found that IgG1 and IgG2 were the most variable across individuals, followed by IgG4 (Fig. 7a). Interestingly, IgG1 and IgG2 demonstrated more inter-individual variability after a 1° than 2° DENV infection (Fig. 7a). This is compatible with a bigger distribution of individual antibody set-points after 1° than 2° dengue. Next, we examined inter-individual differences in DENV4 XR anti-E antibody dynamics from 6 to 18 months post-infection and found that IgG2 kinetics were the most variable across individuals (Fig. 7b). Another aspect of inter-individual variation is the directionality of changes in antibody level over the 6–18 M period, where antibodies may wane for some individuals, but remain stable or rise for others. We found that 50–75% of the post-1° dengue group have XR anti-E antibodies that rise, whereas only 25% of the post-2° group have XR anti-E antibodies that rise (Fig. 7c). This reinforces that rising XR antibodies against E are a more common feature of the post-1° than post-2° dengue response. Finally, we inspected intra-individual variability in antibody levels over time. We found that a high early response was significantly correlated with a high lasting antibody level at 18 months post-1° and post-2° for IgG1 across E, EDIII, and NS1 antigens, and E-IgA (Supplementary Figs. S16 and S17). Anti-E IgG3 and IgG4 demonstrated a higher correlation coefficient after 2° than 1° DENV infection, whereas E-IgG2 demonstrated this relationship more after 1° than 2° DENV infection (Supplementary Fig. S17). Thus, we identified that DENV4 XR E-binding IgG2 and IgG4 levels and kinetics are a highly variable antibody subset across individuals.

## Discussion

In this study, we comprehensively evaluated longitudinal antibody kinetics up to 18 months following 1° and 2° DENV infections, detailing responses for 84 antibody subsets defined by viral antigen, isotype, subclass, and cross-reactivity. This work highlights several novel conceptual insights into flavivirus immunity. First, it challenges the traditional assumption that all cross-reactive antibody levels wane after 1° infection. Instead, we observed subsets (IgG1, IgG2, IgA) that rise from 6 to 18 months post-infection. Second, it demonstrates that antibody dynamics varies not only by infection history but also by antibody specificity (i.e., infecting serotype versus cross-reactive) and by epitope/domain. Furthermore, we detected substantial DENV-binding IgA, IgM, and IgG3 seropositivity at 18 months post-infection, indicating longer persistence than expected for these isotypes. Together, these observations suggest that the humoral response to DENV is not homogenous but comprises multiple differently regulated antibody subsets, each with unique kinetic profiles influenced by prior infection history and other intrinsic factors.

Numerous historical studies show short-term cross-protection against disease with other DENV serotypes that lasts up to ~2 years, suggesting that cross-protection is temporary and wanes^28,29,47–52^. While DENV-binding and neutralizing antibodies certainly wane from their peak post-1° response in early convalescence, as in the Cv-3M and 3–6 M periods from this and other studies^30–32^, the underlying assumption has been that antibodies continue to wane on the scale of years, into the period of subsequent risk for severe dengue, which occurs 3–6 years later. Yet we observed a significant rise in XR E-IgG, particularly the IgG1 and IgG2 subclasses, from 6 to 18 months post-1° DENV infection. This finding contrasts with prior reports on declining antibody levels^53^. Several factors may explain this discrepancy. First, differences in assays used and in statistical power, as well as the modeling approach, may have impacted kinetic changes detected in prior studies. Second, aggregation of antibody responses without distinction of DENV homologous versus XR antibodies might have masked divergent trends. Specifically, we observed stable post-1° long-term total anti-E IgG (i.e., against the infecting serotype) but a rising XR antibody subset; thus, we infer that the type-specific antibody response may wane. In line with our finding, several prior studies show that post-1° heterotypic DENV neutralizing antibodies, heterotypic DENV hemagglutination-inhibition antibodies, and DENV E and fusion loop binding antibodies rise post-1° and wane post-2° dengue^9,30,31,37,54^. Prior studies with longer term data suggest that this rise may stabilize on the scale of multiple years post-1° DENV infection^30,31^. Together, this indicates that rising post-1° antibodies is a reproducible finding across distinct measurements and specifically observed in the heterotypic antibody subset. Our study supports these prior findings and additionally contributes that E-binding cross-reactive IgG1, IgG2, and IgA specifically rise. Moreover, our data indicate that XR epitopes on E domains I/II, but not E domain III, contribute to the post-1° rise in XR E-IgG, as XR E-IgG rises post-1° infection but XR EDIII-IgG remains stable. While prior studies show that a low or intermediate titer of DENV-binding iELISA antibody is associated with severe disease in multiple cohorts, it remains unknown whether a 6–18 M rising antibody kinetic has the potential to contribute to this phenomenon^14,30^.

Interestingly, a similar rise in anti-DENV antibodies has been documented following 1° ZIKV infection^19,31^. Here, we show that this phenomenon also occurs in the reciprocal direction (i.e., DENV and ZIKV XR antibody rise after 1° DENV infection). Prior studies found that neutralizing antibody titers against a heterotypic DENV serotype continued to rise 2–3 years after 1° DENV infection in a pediatric hospital study and up to 7 years in a community-based cohort^9,31^. Thus, the rising kinetic of XR antibodies may be a broader feature of 1° flavivirus infections and may reflect a change in the antigenic composition over time.

We additionally tested whether antibody kinetics differed according to the infecting DENV serotype (DENV1 versus DENV3). In this cohort, inclusion of infecting serotype explained little additional variability in antibody decay rates and did not alter the primary contrasts between infection histories or antibody response types. This likely reflects both the close antigenic relationship between DENV1 and DENV3 and limited power to detect serotype-specific effects in the available sample set. We therefore cannot exclude the possibility that antibody kinetics differ by infection serotypes, including those not incorporated in this study (e.g., DENV2 or DENV4); this should be addressed in future studies with broader serotype coverage.

Another factor that might modulate the risk for disease severity is the significant rise of XR E-IgA and XR NS1-IgA antibodies from 6 to 18 months post-1° DENV infection. While E-IgA has previously been associated with neutralization and interference with IgG-mediated enhancement of viral replication in vitro, we recently linked elevated levels of NS1-IgA with increased severity of dengue disease^44,55,56^. Our data indicate a need to monitor XR E-IgG, XR E-IgA, and XR NS1-IgA over years to better understand how this may modulate the potential for antibody-mediated risk on the scale of years.

In contrast to a 1° DENV immune response, post-2° DENV antibodies are thought to be broadly protective against the DENV1–4 serocomplex^24^. Consistent with earlier findings, our data confirm distinct antibody trajectories across 1° versus 2° DENV infections, notably the earlier attainment of maximal observed levels of anti-E IgG, particularly IgG1 and IgG2, and the sustained higher levels of XR E-IgG (IgG1, IgG2, IgG4) at 18 months after 2° compared to 1° DENV infections^40^. In the post-2° DENV immune response, we and others have found that the difference in the magnitude between E-IgG and EDIII-IgG gets wider over time, unlike a 1° response, suggesting that the 2° immune response more efficiently selects for antibodies targeting epitopes on E outside of EDIII^53^. This is concordant with prior studies reporting the selection of potently neutralizing quaternary antibodies, which have footprints on E domains I and II^34,57,58^. Thus, in alignment with many studies, we find that the 2° DENV immune response selects a XR antibody repertoire more efficiently than a 1° response, which may contribute to subsequent protection from severe disease.

Antibodies against NS1 have been minimally studied longitudinally, but NS1-IgG may participate in protection by limiting viral dissemination and vascular leak during subsequent infection^38,59–61^. Alternatively, XR NS1-IgA can promote dengue pathogenesis by stimulating neutrophil activation in 2° infection^44^. We draw several important observations regarding NS1 antibodies. First, NS1-IgG against the infecting serotype wanes significantly after both 1° and 2° DENV infection, unlike E-IgG. Second, the magnitude of the 1° immune response was higher against the infecting serotype than against XR antigens, and this bias is greater for NS1 than for E protein, suggesting that NS1 antibodies are likely predominantly type-specific. Third, the XR NS1-IgG antibodies were stable even though overall NS1-IgG waned, suggesting that type-specific anti-NS1 antibodies waned over time. Finally, we observed a delayed initiation of antibodies to NS1 relative to E protein, consistent with a prior study^62^. This delayed onset, along with the differential durability and type-specificity of NS1 antibodies, highlights fundamental differences in the kinetics and composition of antibody responses against structural versus non-structural viral proteins and aligns with findings from HIV, influenza, and SARS-CoV-2^63–66^.

With regard to the early kinetic reflecting the initiation of the immune response, our data suggests a temporal hierarchy in the establishment of humoral immunity. For a given antigen, antibodies binding to the infecting serotype displayed different kinetics than XR antibodies, suggesting that type-specific and XR antibodies follow different dynamics. Further, following 1° infection, XR IgG responses measured against XR EDIII and XR NS1 increased more steeply between convalescence and 3 months post-infection than responses measured using the homologous antigen from the infecting serotype. This suggests a delay in the initiation of the cross-reactive response relative to homologous response. Surprisingly, kinetic differences were observed even among XR antibodies targeting distinct domains within the same antigen. For instance, following 1° infection, EDIII-specific XR antibodies arise later than those targeting EDI/II. During 2° infection, the pattern reversed: EDIII-reactive antibodies waned more rapidly than E-binding antibodies. These different kinetics suggest differential selection of epitope-specific populations over time.

Our approach of deconvoluting the antibody response by related viruses reveals important insights regarding sub-antigen epitopes. It is intriguing that upon 1° infection, the kinetics of antibodies targeting a given domain or protein (e.g., EDIII or NS1) differed depending on the virus used as antigen. Although these proteins share homologous structures, they present different epitope landscapes due to amino acid divergence. As a result, antigens from the infecting serotype versus DENV4/2, or even ZIKV, capture some overlapping and some non-overlapping subsets of antibodies, such that the difference in antibody dynamics reflect epitope-level differences within the cross-reactive antigen. Altogether, these findings indicate that antibodies targeting different epitopes may exhibit distinct kinetics. If epitope-specific antibodies have distinct dynamics, it is possible that protective and enhancing antibodies can have different dynamics. For example, the stoichiometry of virion occupancy by antibodies can shift in vitro phenotypes from virus neutralization to enhancement of viral replication^67^. Thus, the qualitative composition of cross-reactive antibody subsets (e.g., the epitopes they recognize in human blood) may have implications for disease protection and risk, and our findings suggest that the composition of the cross-reactive antibody pool is dynamic.

Finally, comprehensive evaluation of minor human plasma immunoglobulin constituents (e.g., IgA, IgM, IgG3, and IgG4) revealed surprising insights. First, we identified long-lasting antiviral IgA, IgM, and IgG3 antibodies, indicated by substantial seropositivity at 18 months post-1° and post-2° DENV infection. While these antibody types are often considered short-lived and a hallmark of recent infection, we find this is not the case when measured with sensitive and specific assays^68,69^. Others have reported such long-lasting flavivirus antibodies as well^61,70,71^. Previously, we found that long-lasting E-IgM and antibody-dependent complement deposition and virolysis were associated with protection from subsequent symptomatic DENV3 infection and severe dengue disease^43,72^. Second, we found that E-IgG4 continues to rise from 6 to 18 months after 1° and 2° DENV infection and demonstrated higher seropositivity after 2° than 1° DENV infection. Alternatively, the rise in DENV E-binding IgG4 antibodies may be due to long-term affinity maturation and production of IgG4 with greater affinity over time, resulting in higher MFI readouts. A rising trajectory can thus be a consequence of an increasing amount of DENV-binding immunoglobulin, as well as increasing affinity. Both possibilities are not mutually exclusive. Recent studies indicate that class-switch toward IgG4 regulates the immune response to lessen antibody-dependent phagocytosis and complement deposition, which may aid in minimizing pathology and inflammation in subsequent DENV infection^73^. In fact, we have consistently observed pre-infection IgG4 antibodies to both E and NS1 to be associated with protection from both symptomatic DENV3 infection and severe dengue disease^43,72^. Third, IgA, IgG2, IgG3, and IgG4 demonstrated higher seropositivity at 18 months post-infection for E than EDIII or NS1, indicating that antigenic biases exist by antibody type. This may result from differences in how each viral antigen is exposed and processed during infection, as well as from distinct cytokine environments elicited by structurally and spatially distinct antigens (virion versus NS1), which can differentially prime the immune response.

In addition, certain isotype–antigen combinations within XR antibody subsets, such as E-IgG2 and E-IgG4, show marked interindividual variability at 18 months post-1° infection. Also, a subset of individuals remain seropositive to cross-reactive antigens with the presence of minor isotypes like IgA, IgM, IgG3, and IgG4, while others had undetectable levels. It is possible that there may be more IgA and IgM than we observed, as some experimental signal may be lost due to the abundance of IgG in human plasma. Such heterogeneity may have important implications for long-term immunity by delineating the antibody population into distinct response groups.

A significant strength of our study is the detailed characterization of antibody kinetics across multiple dimensions of the DENV immune response in natural infections, including antibody isotype and subclass, antigen specificity, and virus cross-reactivity, using multiplex assays that offer higher resolution than traditional assays. Additionally, our longitudinal sampling approach provides rigorous estimates of antibody dynamics and durability. Bootstrap-based mixed models were used as the primary analytical approach, with robustness assessed through sensitivity analyses based on Bonferroni-corrected Wilcoxon tests and a unified Bayesian hierarchical model, showing that the results are consistent across inference frameworks and reflect underlying biology rather than model-specific artefacts.

However, our study also has limitations, such as a relatively small sample size, which may result in lower statistical power to detect differences in waning over time. Primary versus secondary immune status was inferred serologically as per widely applied and conventional standards in the field, though some level of misclassification is possible. Our convenience sampling design may limit generalizability. Also, we evaluated DENV1 and DENV3 serotype infections, and dynamics may differ somewhat for other DENV serotypes. Moreover, our analyses focused on antibody binding and did not directly assess functional or neutralizing activity, which may more closely reflect protective immunity. Some cohort effects exist that could impact these results. For the 2° cases, the serotype and timing of prior exposure may influence antibody dynamics, but this information was not available as cases were enrolled during illness. Infection histories from 2° individuals were unknown, and it is possible that antibody kinetics are further modulated by the exact number of flavivirus exposures or the serotype(s) of prior infection. Of note, our longitudinal sampling timepoint is up to 18 months post-infection, and longer-term dynamics on the scale of 3–4 years after dengue will be needed to evaluate antibody dynamics in the typical risk period for subsequent disease; such studies from our Nicaraguan cohort are being reported separately. This study did not include participant groups with differences in infection or disease outcome; thus, the relationship between antibody kinetics and clinical risk or protection must be further investigated. It is important to note that our samples are derived from an endemic region with sustained DENV transmission, without other flavivirus immunization. Importantly, although occasional boosting from unrecognized DENV exposures may contribute to stable or rising antibody levels in some subsets, the distinct long-term waning or rising patterns observed across specific antibody subsets suggest that boosting alone is unlikely to fully account for our main findings. However, boosting due to subclinical re-exposures cannot be ruled out^74^. Antibody dynamics following a single 1° exposure in travelers from non-endemic regions may follow different trajectories. This study included hospital pediatric dengue cases, which may differ from the antibody dynamics of community infections with asymptomatic or mild disease.

In conclusion, this study reveals complex antibody dynamics following 1° and 2° DENV infections. The rise of XR antibodies post-1° infection and the differences in antibody trajectories of E, EDIII, and NS1 add critical insights to our understanding of dengue immunity. These findings have important implications for vaccine design, diagnosis, epidemic surveillance, and modeling.

## Methods

### Study design

The Pediatric Dengue Hospital-based study is an ongoing longitudinal investigation at the Hospital Infantil Manuel de Jesús Rivera in Managua, Nicaragua^75,76^. Since 2005, children aged 0–14 years who present within 1–7 days of symptom onset are invited to participate if they meet case definitions for dengue. Following consent and, in children ≥6 years old, assent, participants undergo standard clinical evaluations and, if needed, hospitalization based on established criteria^77^. Sex of participants was collected by self-report at clinical evaluation. Both sexes were included; sex was included as a fixed-effect covariate in all linear mixed-effects and Bayesian models. Sex-disaggregated counts are reported in Supplementary Table 1. No formal sex-stratified analyses were performed.

Blood samples are collected at the time of hospital presentation (acute phase), daily for days 1–3 of illness, and again in early convalescence (14–28 days post-symptom onset)^76,77^. Laboratory testing confirms infections using RT-PCR, virus isolation, and/or serological assays for dengue virus (DENV). Clinical data, comprising >150 variables (including fluid intake/output), are recorded every 12 h in a case report form and digitized via a double-data–entry protocol with daily and weekly quality control checks. A standardized clinical history questionnaire captures demographic and epidemiological information, ensuring consistency and completeness. In addition, children may consent to a longitudinal follow-up of convalescent samples at 3, 6, 12, and 18 months after illness onset. These follow-up visits collect healthy plasma and PBMC samples. For this study, samples were collected from August 24, 2010, to November 26, 2013.

Laboratory confirmation of DENV infection specifically involves either seroconversion of DENV-specific IgM antibodies between acute- and convalescent-phase samples with MAC-ELISA, a fourfold or greater rise in total antibodies measured by inhibition ELISA, virus isolation in C6/36 *Aedes albopictus* cells, or RT-PCR amplification of viral RNA^16,78,79^. DENV serotypes (DENV1–4) are identified by virus isolation or RT-PCR^16,79^. A primary DENV infection is classified by an iELISA titer below 2560 in convalescent-phase samples; conversely, 2° infections are indicated by titers of 2560 or higher in convalescent-phase samples^80^. The term 2° refers to two or more DENV infections. Dengue hemorrhagic fever is defined as per the WHO 1997 criteria^81^.

This study was reviewed and approved by the Institutional Review Boards of the University of California Berkeley and the Ministry of Health of Nicaragua. Parents or legal guardians of all participants provided written informed consent, and participants 6 years of age and older provided assent.

### Multiplex assays

Recombinant envelope (E) and NS1 proteins of DENV1–4 and ZIKV were purchased from the Native Antigen and randomly biotinylated. Site-specifically biotinylated E domain III (EDIII) of DENV1–4 and ZIKV were provided by Lakshmanane Premkumar, University of North Carolina, Chapel Hill. Antigens were coupled to avidin-coated MagPlex beads and pooled for use in a multiplex assay. Briefly, diluted plasma samples (four consecutive fourfold dilutions beginning at 1:50) were incubated with the antigen-coupled microspheres in 384-well plates for 90 min at 37 °C under continuous shaking at 1200 rpm. Following incubation, the plates were washed with PBS containing 0.1% bovine serum albumin and 0.02% Tween 20 using a Tecan Hydrospeed automatic magnetic washer. Antigen-specific Ig antibodies were detected by adding phycoerythrin (PE)-conjugated mouse anti-human IgG, IgM, IgA, or IgG1–4 as indicated (SouthernBiotech). Median fluorescence intensity (MFI) was measured on an iQue3 cytometer (Intellicyt). Five DENV-naïve human serum samples collected during the acute phase of a non-DENV febrile-illness were included on each plate as negative controls. Background MFI values were calculated as the mean signal among naïve individuals plus three standard deviations for each antigen–antibody combination. Log₂ transformation was applied prior to background correction; thus, values represent log₂ signal relative to background (log₂(MFI)–log₂(background)). MFI were included when ≥50 beads were acquired per region. Each plasma sample was tested with one technical replicate per antibody isotype/subclass.

### Statistical analyses

To compare primary and secondary infections, as well as all isotypes and antigens at the same dilution, the 1:800 dilution was used. We conducted three primary comparisons: (1) primary versus secondary infection status (IR), (2) viral antigen (E, EDIII, and NS1), and (3) antibody response type (against incoming serotype, DENV cross-reactive, and ZIKV cross-reactive). Antibody kinetics were analyzed using three complementary statistical frameworks. To compare the antibody dynamics between primary and secondary infection groups, antibody decay rates were first estimated using LMMs, which constituted the *primary analytical framework* for this study. Second, a Bayesian hierarchical linear mixed model (BLMM) was implemented as a sensitivity analysis to jointly estimate decay parameters within a multilevel framework and assess robustness by model approach and multiple comparisons through partial pooling and shrinkage. In addition, non-parametric Wilcoxon tests were applied to individual-level fold-change data for the three primary comparisons (by IR, viral antigen, and antibody response type). Of 40 enrolled post-primary dengue participants, one with a missing early convalescent sample was excluded from individual-level analyses, yielding an analytical cohort of *n* = 39 post-primary participants. Within this analytical cohort, three additional missing values at the 18-month timepoint were median-imputed for the LMM and Wilcoxon analyses using the median of the corresponding group with matching infecting serotype, viral antigen, and antibody isotype/subclass. The 18-month magnitude and seropositivity panels report *n* = 36 observed values.

#### LMMs

Each factor was examined simultaneously, considering the other relevant factors by embedding analyses within multiple stratifications (e.g., primary versus secondary infection comparisons were analyzed separately for each antigen, antibody isotype, and subclass, and antibody response type). Analyses were conducted using LMMs with the general form:

1 $${\log }_{2}({{{\rm{MFI|}}}}B) =	 {\beta }_{0}+{\beta }_{1}{{\cdot }}A+{\beta }_{2}{\cdot} {{{\rm{DPSO}}}}+{\beta }_{3}{{\cdot }}({{{\rm{DPSO}}}}-100)+{\beta }_{4}{{\cdot }}({{{\rm{DPSO}}}}-200) \\ 	+{\beta }_{5}{{\cdot }}(A\times {{{\rm{DPSO}}}})+{\beta }_{6}{{\cdot }}(A\times ({{{\rm{DPSO}}}}-100)) \\ 	+{\beta }_{7}{{\cdot }}(A\times ({{{\rm{DPSO}}}}-200))+{\beta }_{8}{\cdot} {{{\rm{Age}}}}+{\beta }_{9}{\cdot} {{{\rm{Sex}}}}+{\mu }_{{{{\rm{participant}}}}}+\varepsilon$$

where *A* is the single primary factor of interest (IR, antigen, or antibody response type), and *B* represents the outcome level of antibody as log_2_(MFI). We incorporated piecewise linear splines for days post-symptom onset (DPSO) at knots of 100 and 200 days to capture immunologically known phases: an initial rapid decay in antibodies, followed by stabilization and then a long-term homeostasis at later times. Therefore, *β*_2_ captures the slope (rate of change in antibody levels) from day 0 to 100 ( ~ 3 months), *β*_3_ is the change in slope from 100 to 200 days (3 to 6 months), and *β*_4_ is the change in slope from 200 to 540 days (6 to 18 months). A random intercept (*μ*_participant_) was included for each participant to account for repeated measures over time from the same individual. Age and sex were included as covariates in all models.

This generalized formula varied across factors. For example, viral antigen has three outcomes/categories, including envelope (E protein), E protein domain III (EDIII), and nonstructural protein 1 (NS1), and this was evaluated using the following formula:

2 $${\log }_{2}({MFI|B})=	 {\beta }_{0}+{\beta }_{1}{\cdot} {{{\rm{EDIII}}}}+{\beta }_{2}{\cdot} {{{\rm{NS}}}}1+{\beta }_{3}{\cdot} {{{\rm{DPSO}}}}+{\beta }_{4}{{\cdot }}({{{\rm{DPSO}}}}-100) \\ 	+{\beta }_{5}{{\cdot }}({{{\rm{DPSO}}}}-200)+{\beta }_{6}{{\cdot }}({{{\rm{EDIII}}}}\times {{{\rm{DPSO}}}})+{\beta }_{7}{{\cdot }}({{{\rm{EDIII}}}}\\ 	 \times ({{{\rm{DPSO}}}}-100))+{\beta }_{8}{{\cdot }}({{{\rm{EDIII}}}}\times ({{{\rm{DPSO}}}}-200)) \\ 	+{\beta }_{9}{{\cdot }}({{{\rm{NS}}}}1\times {{{\rm{DPSO}}}})+{\beta }_{10}{{\cdot }}({{{\rm{NS}}}}1\times ({{{\rm{DPSO}}}}-100)) \\ 	+{\beta }_{11}{{\cdot }}({{{\rm{NS}}}}1\times ({{{\rm{DPSO}}}}-200))+{\beta }_{12}{\cdot} {{{\rm{Age}}}}+{\beta }_{13}{\cdot} {{{\rm{Sex}}}}+{\mu }_{{{{\rm{participant}}}}}+\varepsilon$$

The antibody response type variable, which has four categories (homologous/infecting virus, DENV2-cross-reactive, DENV4-cross-reactive, or ZIKV-cross-reactive), was analyzed similarly. Figures 1–3 were generated using models evaluating primary versus secondary infections as the main effect (i.e., infection history group). Figure 4 instead evaluates antigen as the primary factor in the mode (i.e., E, EDIII, and NS1), and Fig. 5 evaluates antibody response type (i.e., infecting serotype, DENV2 XR, DENV4 XR, and ZIKV XR) as the main effect.

To robustly quantify uncertainty, we performed case bootstrap resampling at the participant level, refitting each model 1000 times^82^. For each bootstrap replicate, marginal predictions of log₂(MFI) were computed at 20 days, 3 months ( ≈ 100 days), 6 months ( ≈ 200 days), and 18 months ( ≈ 550 days), enabling comparisons of antibody magnitude distributions across these timepoints. Additionally, we derived daily rates of MFI change within each piecewise segment (20–100 days, 100–200 days, and 200–550 days) and averaged these slopes across replicates, providing a quantitative basis for comparing antibody kinetics (slopes) across groups. These daily rates of change in log₂(MFI) were subsequently transformed into half-lives using the formula:

3 $${{{\rm{Half}}}}{\mbox{-}}{{{\rm{life}}}} \, ({{{\rm{years}}}})=(-1/[{\Delta \log }_{2}({MFI})/{day}])/365.25$$

Under this convention, a half-life of zero implies an infinite half-life (no net change in antibody concentration), negative values represent a rising antibody response (“negative half-life”), and positive values reflect waning antibody concentrations (“positive half-life”).

Differences between groups were assessed using contrasts in decay rates obtained from a non-parametric bootstrap of individual trajectories. For each antibody subset and time interval, a subset was classified as significantly waning or rising when the 95% confidence interval of its decay rate excluded zero (i.e., infinite half-life). In addition, within each stratum, differences between groups were evaluated using a confidence interval overlap criterion, whereby a group was considered to differ from the reference group if its 95% confidence interval for the decay rate did not overlap the reference group’s point estimate.

#### BLMM

To identify the most probable underlying distributions and to try to reduce the risk of false-positive findings in this high-dimensional setting, we fit a single unified BLMM using the same spline-based structure as the primary mixed-effects analysis. Antibody binding was modeled on the log_2_(MFI) scale using a Gaussian Bayesian multilevel model with an identity link. Days post-symptom onset (DPSO) was parameterized as a piecewise linear spline with three intervals (0–100, 100–200, and >200 days). These spline terms were interacted with infection history (IR), antigen, antibody response type (incoming virus vs cross-reactive), and antibody isotype/subclass, to estimate compartment-specific decay rates. Each antibody compartment is defined by the full combination of these factors (IR × antigen × antibody response type × isotype/subclass). To account for the hierarchical and repeated-measures structure of the data, we included random intercepts and random DPSO slopes at the antibody-compartment level, allowing both baseline antibody levels and decay rates to vary across compartments. In addition, we included nested random intercepts for individuals within antibody compartments and subject-level random intercepts, capturing within-individual correlation across repeated measurements while allowing partial pooling across compartments. Weakly informative priors were placed on all fixed and random effects. The model was fit using four Markov chain Monte Carlo chains with 1500 iterations each (500 warmup), yielding 4000 post-warmup posterior draws.

Our Bayesian model is the following:

4 $${\log }_{2}({{MFI}}_{{{{\rm{i}}}}})={\mu }_{{{{\rm{i}}}}}+{\varepsilon }_{{{{\rm{i}}}}},{\varepsilon }_{{{{\rm{i}}}}} \sim {{{\rm{Normal}}}}(0,{{{{\rm{\sigma }}}}}^{2})$$

*μ*_*i*_=*β*_0_+*f*(DPSO_0–100,i_, DPSO_100–200,i_, DPSO_200+,i_, IR_*i*_, Antigen_*i*_, Antibody response type_*i*_, Ab isotype/subclass_*i*_) + *β*_Age_·Age_*i*_ + *β*_Sex_·Sex_*i*_ + *u*_0_(Ab compartment_*i*_) + *u*_1_(Ab compartment_*i*_)·DPSO_0–100,i_ + *u*_2_(Ab compartment_*i*_)·DPSO_100–200,i_ + *u*_3_(Ab compartment_*i*_)·DPSO_200+,i_ + *u*_0,0_(Participant ID_*i*_) + *u*_0,1_(Participant ID_*i*_)·I(DPSO_0–100,i_) + *u*_0,2_(Participant ID_*i*_)·I(DPSO_100–200,i_) + *u*_0,3_(Participant ID_*i*_)·I(DPSO_200+,i_) where:

${\varepsilon }_{{{{\rm{i}}}}}$ is the residual error term, assumed *ε*_i_ ~ Normal(0, *σ*^2^).

*f*(·) represents the fixed-effect spline terms for the three DPSO intervals and their interactions with immune response (IR), antigen, and antibody response type.

*u*_0_(Participant ID_i_) is a random intercept for each individual (Participant ID).

*u*_0_(Ab compartment_i_), *u*_1_(Ab compartment_i_), *u*_2_(Ab compartment_i_), and *u*_3_(Ab compartment_i_) are the random intercept and random slopes for the three DPSO spline segments for each Ab compartment.

*u*_0,0_(Participant ID_i_), *u*_0,1_(Participant ID_i_), *u*_0,2_(Participant ID_i_), and *u*_0,3_(Participant ID_i_) are participant-specific random intercept deviations for the overall baseline and for each of the three DPSO time segments, allowing individual-level shifts in antibody level within each segment.

Ab compartments represent the strata defined by the unique combination of infection response (primary/secondary), antigen, antibody response type pattern, and antibody isotype/subclass.

Priors:

All fixed-effect coefficients were assigned Normal(0, 1) priors. The standard deviations of the Ab compartment’s random effects, the participant’s random effects, and the residual error term were assigned Student-t distribution with 3 degrees of freedom, centered at zero, with unit scale (Student-t(3, 0, 1)) priors, providing a weakly informative distribution centered at zero with moderate scale and heavy tails that allow larger variance values when supported by the data. All parameters were estimated within a Bayesian hierarchical framework, with population-level effects, group-level effects, and variance components jointly inferred from the posterior distribution.

Group differences were assessed using the same decay-rate contrast and decision criteria as for the LMMs, but with inference based on posterior distributions and 95% credible intervals.

#### Wilcoxon analyses

As neither the bootstrap-based LMMs nor the BLMM explicitly estimate individual-level random slopes at each antibody compartment level, we performed Wilcoxon tests on individual-level data. For each participant, the annualized fold-change was defined as the difference in log_2_(MFI) between two timepoints within each time segment (Cv–3 and 6–18 M). All tests were two-sided.

For infection-group comparisons (1), fold-change values from secondary dengue cases were compared to those from primary dengue cases using unpaired Wilcoxon rank-sum tests, stratified by antigen, antibody response type, antibody isotype or subclass, and time period.

For antigen-specific comparisons (2), fold-change in EDIII- and NS1-binding antibodies was compared to the corresponding E-binding fold-change within the same individuals using paired Wilcoxon signed-rank tests, stratified by infection group, antibody response type, isotype or subclass, and time segments.

For antibody response type (3), fold-change values for each cross-reactive response group (DENV2-XR, DENV4-XR, ZIKV-XR) were compared to the incoming-virus response within individuals using paired Wilcoxon signed-rank tests, stratified by antigen, isotype, or subclass, and time period.

To control for multiple testing within each analysis set, p-values were adjusted using the Bonferroni correction. Effect sizes were calculated as rank-biserial correlations, with 95% confidence intervals estimated for each comparison. Test statistics, raw and adjusted p-values, effect sizes, and confidence intervals are reported in Source Data.

#### Seroprevalence analyses

Finally, seroprevalence, defined as the proportion of samples exceeding three standard deviations above the naïve mean value, was calculated at 18 months post-infection for each factor level (infection status, antigen type, antibody response type, and antibody isotype/subclass).

All statistical analyses were conducted in R (version 4.3.1). Data processing and visualization were performed using the tidyverse package suite, including dplyr, tidyr, and ggplot2. Frequentist LMMs with case bootstrap resampling were fitted using lme4 and lmeresampler. BLMMs were implemented using brms with a Stan backend (rstan). Non-parametric Wilcoxon tests, including Bonferroni correction and effect-size estimation, were performed using rstatix. Additional analyses used ggeffects, lspline, emmeans, and foreach.

### Reporting summary

Further information on research design is available in the Nature Portfolio Reporting Summary linked to this article.

## Supplementary information

Supplementary Information

Reporting summary

Transparent Peer Review file

## Source data

Source Data

## Data Availability

All data associated with this study are present in the main text or Supplementary Information. Simulated datasets for the statistical analyses are available via Zenodo^83^. After obtaining approval from the UC Berkeley Committee for the Protection of Human Subjects, individual data, which are subject to human-subjects protections, can be shared with external researchers for figure reproduction. For access arrangements, please contact E.H. at eharris@berkeley.edu. Standard data and material transfer agreements govern all data used in this study. Source data are provided with this paper.

## Code availability

Simulated datasets and codes to reproduce the statistical analyses presented in this study are publicly available at Zenodo^83^.
