# Supplementary material for: Longitudinal antibody profiling after dengue reveals distinct dynamics by antibody specificity over 18 months
Source: Nat Commun. 2026 Jun 21;17:7808. doi: 10.1038/s41467-026-74406-0 (PMC13438135; doi:10.1038/s41467-026-74406-0)
Supplement: Supplementary file 1 [file 41467_2026_74406_MOESM1_ESM.pdf]

Supplementary Materials

Longitudinal antibody profiling after dengue reveals distinct dynamics by antibody specificity over 18 months

Sandra Bos[1,2*], Tulika Singh[1*], José Victor Zambrana[3,4*], Elias Duarte[1], Reinaldo Mercado-Hernandez[1], Julia Huffaker[1], Aaron Graber[1], Angel Balmaseda[5,6], Eva Harris[1†]

[1] Division of Infectious Diseases and Vaccinology, School of Public Health, University of California, Berkeley, Berkeley, CA

[2] Department of Infectious Diseases and Microbiology, School of Public Health, University of Pittsburgh, Pittsburgh, PA, USA

[3] Sustainable Sciences Institute, Oakland, CA

[4] Department of Epidemiology, School of Public Health, University of Michigan, Ann Arbor, MI

[5] Laboratorio Nacional de Virología, Centro Nacional de Diagnóstico y Referencia, Ministerio de Salud, Managua, Nicaragua

[6] Sustainable Sciences Institute, Managua, Nicaragua

*Contributed equally

† Corresponding author: Eva Harris eharris@berkeley.edu

Table of contents

Supplementary Note 1 **4**

Bayesian hierarchical linear mixed model diagnostics *4*

Concordance of primary versus secondary antibody dynamics across models and Bonferroni-corrected Wilcoxon tests *4*

Supplementary Tables **5**

Supplementary Table 1. Study participant characteristics by post-primary versus post-secondary dengue groups. *5*

Supplementary Table 2. Concordance of primary versus secondary antibody dynamics across linear mixed models, Bayesian linear mixed models, and Bonferroni-corrected Wilcoxon tests. *6*

*Supplementary Table 3. Summary of Bayesian random-effect variance components and convergence diagnostics.** *7*

*Supplementary Table 4. Bayesian random-effect variance and correlation parameters at the antibody-compartment level.** *7*

Supplementary Table 5. Population-level fixed-effect estimates from the Bayesian hierarchical model. *8*

Supplementary Figures **9**

Supplementary Figure 1. Schematic representation of key concepts regarding cross-reactive antibody sub-populations during a primary versus a secondary anti-DENV immune response. *9*

Supplementary Figure 2. Schematic of experimental and analysis approach. *10*

Supplementary Figure 3. Bayesian linear mixed-model analysis of homologous E-binding antibody responses after primary and secondary DENV infection. *11*

Supplementary Figure 4. Model fit atop individual antibody trajectories for homologous antibody responses against the infecting serotype. *12*

Supplementary Figure 5. Bayesian linear mixed-model analysis of homologous NS1-binding antibody responses after primary and secondary dengue. *13*

Supplementary Figure 6. Bayesian linear mixed-model analysis of DENV serocomplex cross-reactive anti-E antibody responses after primary and secondary dengue. *14*

Supplementary Figure 7. Model fit atop individual antibody trajectories for antibody responses against the cross-reactive DENV4 serotype. *15*

Supplementary Figure 8. Cross-reactive antibody responses against DENV4 E protein. *16*

Supplementary Figure 9. Bayesian linear mixed-model analysis of DENV serocomplex cross-reactive anti-NS1 antibody responses after primary and secondary dengue. *17*

Supplementary Figure 10. DENV serocomplex cross-reactive anti-NS1 antibodies are stable after primary dengue but wane post-secondary dengue. *18*

Supplementary Figure 11. Bayesian linear mixed-model analysis of homologous EDIII-binding antibody responses after primary and secondary dengue. *20*

Supplementary Figure 12. Bayesian linear mixed-model analysis of DENV serocomplex cross-reactive anti-EDIII antibody responses after primary and secondary dengue. *21*

Supplementary Figure 13. Antibody responses against homologous EDIII protein after primary and secondary dengue. *22*

Supplementary Figure 14. Antibody responses against cross-reactive EDIII protein after primary and secondary dengue. 23

Supplementary Figure 15. Model fit atop individual antibody trajectories for cross-reactive antibody responses against ZIKV. 24

Supplementary Figure 16. Relationship between maximal observed early IgG1 antibody response and the level remaining at 18 months post-infection by antigen. 25

Supplementary Figure 17. High early antibody responses against E protein correlates with high antibody levels at 18 months post-infection for multiple antibody subsets. 26

Supplementary Figure 18. Posterior predictive check of the Bayesian model. 27

Supplementary Figure 19. Bayesian model convergence diagnostics for fixed-effect parameters. 29

Supplementary Note 1

Bayesian hierarchical linear mixed model diagnostics

Model fit and convergence were evaluated using posterior predictive checks and standard Markov chain Monte Carlo (MCMC) diagnostics. Posterior predictive distributions closely matched the observed data, and residuals exhibited an approximately linear relationship with fitted values, indicating that the spline-based structure adequately captured the underlying antibody kinetics (Supplementary Fig S18). All model parameters showed good convergence, with $\hat{R}$ values ≤ 1.05 and effective sample sizes sufficient for stable posterior inference (Supplementary Tables 3–5). Trace plots demonstrated good chain mixing without evidence of non-convergence, and posterior distributions of both fixed and random effects were approximately unimodal and symmetric (Supplementary Fig S19). Overall model explanatory power was high, with a median Bayesian R^2 of 0.92 (IQR = 0.69–0.92).

Concordance of primary versus secondary antibody dynamics across models and Bonferroni-corrected Wilcoxon tests

We compared three independent inference frameworks: non-parametric bootstrap linear mixed models (LMM), Bayesian hierarchical linear mixed models (BLMM) and Bonferroni-corrected Wilcoxon tests applied to individual-level fold-changes (Supplementary Figures S3, S5, S6, Supplementary Table 2). Across incoming-virus (i.e., against homologous antigen) responses to both E and NS1 during the early Cv–3M interval, all three methods consistently detected significantly different decay dynamics between primary and secondary infections for IgG, IgA, IgM, IgG1, and IgG2, demonstrating strong, method-independent, divergence in early antibody kinetics in primary versus secondary infection. In contrast, results regarding IgG3 were less consistent. IgG4 dynamics was not assessed as the modeled antibody trajectory remained at or below the limit of quantitation over the entire time segment.

For late-phase (6–18M) homologous responses, BLMM detected residual differences between primary and secondary infections, whereas LMM and Wilcoxon tests were less sensitive, with Wilcoxon test showing no difference in antibody dynamics across all isotype/subclass.

Importantly, for cross-reactive DENV4-XR responses against E protein, primary versus secondary differences were again strongly concordant across all three frameworks during Cv–3M for IgG, IgA, IgM, IgG1, and IgG2 (Supplementary Figure S6, Supplementary Table 2). At 6–18M, cross-reactive IgG1 and IgG2 remained significantly different between primary and secondary infections across LMM, BLMM, and Wilcoxon tests, demonstrating a sustained rise of these subclasses following primary infection. In contrast, differences in cross-reactive IgA dynamics between primary and secondary infection, characterized by a rise after primary infection, were detected by both modeling approaches but not by Wilcoxon testing (Supplementary Table 2).

Taken together, this cross-framework concordance demonstrates that the principal biological conclusions, namely, that primary and secondary DENV infections generate distinct antibody kinetics and that cross-reactive IgG1 and IgG2, the main components of the IgG response, rise following primary infection, are robust to statistical methodology and do not depend on any single inference framework.

Supplementary Tables

Supplementary Table 1. Study participant characteristics by post-primary versus post-secondary dengue groups.

		Primary		Secondary	
	**All**	**DENV1**	**DENV3**	**DENV1**	**DENV3**
**Participants**	79	20	20	16	23
**Age (mean (SD))**	8.6	7.6	7.0	10.4	9.7
**Female**	32	10	10	5	8
**Male**	47	10	10	11	15
**DHF**	13	0	0	3	10
**Days from symptom onset (convalescent)** ***(median [IQR])***		16.00 *[13.50, 17.00]*		16.00 *[15.00, 18.50]*	
**Days from symptom onset (3 months)** ***(median [IQR])***		107.50 *[101.00, 114.25]*		111.00 *[103.50, 122.00]*	
**Days from symptom onset (6 months)** ***(median [IQR])***		188.00 *[186.75, 194.25]*		189.00 *[186.00, 196.50]*	
**Days from symptom onset (18 months)** ***(median [IQR])***		562.50 *[551.25, 578.75]*		557.00 *[553.00, 568.00]*	

Supplementary Table 2. Concordance of primary versus secondary dengue antibody dynamics across linear mixed models, Bayesian linear mixed models, and Bonferroni-corrected Wilcoxon tests.

Antibody response	Antigen	Time period	Isotype	Group 1*	Group 2*	Fig.**	LMM signif[#]	BLMM signif[#]	WB p adj[##]
Infecting virus	E	Cv-3M	IgG	P	S	1B	*	*	****
Infecting virus	E	Cv-3M	IgA	P	S	1B	*	*	****
Infecting virus	E	Cv-3M	IgM	P	S	1B	*	*	****
Infecting virus	E	Cv-3M	IgG1	P	S	1B	*	*	****
Infecting virus	E	Cv-3M	IgG2	P	S	1B	*	*	****
Infecting virus	E	Cv-3M	IgG3	P	S	1B	ns	*	ns
Infecting virus	E	Cv-3M	IgG4	P	S	1B	-[†]	-	-
Infecting virus	E	6-18M	IgG	P	S	1C	ns	*	ns
**Infecting virus[††]**	**E**	**6-18M**	**IgA**	**P**	**S**	**1C**	*****	*****	**ns**
Infecting virus	E	6-18M	IgM	P	S	1C	-	-	-
Infecting virus	E	6-18M	IgG1	P	S	1C	ns	*	ns
Infecting virus	E	6-18M	IgG2	P	S	1C	ns	*	ns
Infecting virus	E	6-18M	IgG3	P	S	1C	ns	ns	ns
Infecting virus	E	6-18M	IgG4	P	S	1C	ns	*	ns
Infecting virus	NS1	Cv-3M	IgG	P	S	2B	*	*	****
Infecting virus	NS1	Cv-3M	IgA	P	S	2B	*	*	****
Infecting virus	NS1	Cv-3M	IgM	P	S	2B	*	*	****
Infecting virus	NS1	Cv-3M	IgG1	P	S	2B	*	*	****
Infecting virus	NS1	Cv-3M	IgG2	P	S	2B	*	*	****
**Infecting virus**	**NS1**	**Cv-3M**	**IgG3**	**P**	**S**	**2B**	*****	*****	**ns**
Infecting virus	NS1	Cv-3M	IgG4	P	S	2B	-	-	-
Infecting virus	NS1	6-18M	IgG	P	S	2C	ns	ns	ns
Infecting virus	NS1	6-18M	IgA	P	S	2C	*	ns	ns
Infecting virus	NS1	6-18M	IgM	P	S	2C	-	-	-
Infecting virus	NS1	6-18M	IgG1	P	S	2C	ns	ns	ns
Infecting virus	NS1	6-18M	IgG2	P	S	2C	*	*	ns
Infecting virus	NS1	6-18M	IgG3	P	S	2C	*	*	ns
Infecting virus	NS1	6-18M	IgG4	P	S	2C	-	-	-
DV4 XR	E	Cv-3M	IgG	P	S	3B	*	*	**
DV4 XR	E	Cv-3M	IgA	P	S	3B	*	*	****
DV4 XR	E	Cv-3M	IgM	P	S	3B	*	*	****
DV4 XR	E	Cv-3M	IgG1	P	S	3B	*	*	***
DV4 XR	E	Cv-3M	IgG2	P	S	3B	*	*	****
DV4 XR	E	Cv-3M	IgG3	P	S	3B	*	*	ns
DV4 XR	E	Cv-3M	IgG4	P	S	3B	-	-	-
**DV4 XR**	**E**	**6-18M**	**IgG**	**P**	**S**	**3C**	*****	*****	**ns**
DV4 XR	E	6-18M	IgA	P	S	3C	*	*	ns
DV4 XR	E	6-18M	IgM	P	S	3C	-	-	-
**DV4 XR**	**E**	**6-18M**	**IgG1**	**P**	**S**	**3C**	*****	*****	*****
**DV4 XR**	**E**	**6-18M**	**IgG2**	**P**	**S**	**3C**	*****	*****	*****
DV4 XR	E	6-18M	IgG3	P	S	3C	-	-	-
DV4 XR	E	6-18M	IgG4	P	S	3C	ns	ns	ns

**Groups 1 and 2 indicate the groups being compared (P, primary; S, secondary).*

***The corresponding main-text figure reporting the LMM results for the same comparison is indicated in the Fig. column.*

[#]*Asterisks in LMM signif. and BLMM signif. columns denote significant differences between primary and secondary infection decay rates based on a confidence-interval overlap criterion.*

[##]*Stars in the WB p adj column denote Bonferroni-adjusted p-values from paired Wilcoxon tests (*p < 0.05; **p < 0.01; *p < 0.001).*

[†] *(-) indicates that differences in decay-rate or fold-change were not computed for that condition because the modeled antibody trajectory remained at or below zero over the entire time segment.*

[††]*Bolding shows the divergent main conclusions commented in the Results section.*

Supplementary Table 3. Summary of Bayesian random-effect variance components and convergence diagnostics.*

Random effect group	Levels	Parameter	Estimate	SE	95% CI (Lower)	95% CI (Upper)	$\hat{R}$	Bulk ESS	Tail ESS
Participant ID	79	SD (Intercept)	0.40	0.03	0.34	0.48	1.01	683	1,303
Participant ID x Ab compartments	6,636	SD (Intercept)	0.80	0.01	0.78	0.82	1.00	1,404	2,550

The Bayesian hierarchical model included random intercepts at the participant level (79 participants) and at the participant × antibody-compartment × time-segment level (6,636 levels). Shown are posterior estimates of standard deviations (SD), standard errors (SE), 95% credible intervals (CI), and convergence diagnostics ($\hat{R}$, bulk and tail effective sample sizes).

Supplementary Table 4. Bayesian random-effect variance and correlation parameters at the antibody-compartment level.*

Random effect group	Estimate	SE	95% CI (Lower)	95% CI (Upper)	$\hat{R}$	Bulk ESS	Tail ESS
SD (Intercept)	1.71	0.10	1.52	1.92	1.00	510	988
SD (DPSO 0–100)	0.01	0.00	0.01	0.02	1.01	486	795
SD (DPSO 100–200)	0.00	0.00	0.00	0.00	1.00	1,747	1,944
SD (DPSO ≥200)	0.00	0.00	0.00	0.00	1.00	1,936	2,879
Cor (Intercept, DPSO 0–100)	−0.62	0.05	−0.72	−0.51	1.01	382	857
Cor (Intercept, DPSO 100–200)	−0.44	0.09	−0.61	−0.25	1.00	1,241	1,931
Cor (DPSO 0–100, DPSO 100–200)	0.04	0.11	−0.17	0.25	1.00	1,240	1,797
Cor (Intercept, DPSO ≥200)	−0.50	0.10	−0.69	−0.28	1.00	1,855	2,759
Cor (DPSO 0–100, DPSO ≥200)	0.25	0.11	0.02	0.46	1.00	1,618	2,651
Cor (DPSO 100–200, DPSO ≥200)	0.92	0.05	0.79	0.99	1.00	1,779	2,936

Shown are posterior estimates of standard deviations (SD) and correlations for random intercepts and time-segment–specific slopes across antibody compartments (168 levels). Days post-symptom onset (DPSO) was modeled using three spline segments (0–100, 100–200, ≥200 days). $\hat{R}$ and effective sample sizes (ESS) are reported as convergence diagnostics. Correlation between two different random effects such as intercept and slope, or two slopes [Cor (random effect #1, random effect #2)].

Supplementary Table 5. Population-level fixed-effect estimates from the Bayesian hierarchical model.

A. Main effects*

Parameter	Estimate	SE	95% CI (Lower)	95% CI (Upper)	R̂	Bulk ESS	Tail ESS
Intercept	15.76	0.43	14.86	16.61	1.01	346	483
DPSO 0–100	−0.01	0.00	−0.02	−0.01	1.02	532	551
DPSO 100–200	−0.00	0.00	−0.00	0.00	1.00	1,418	2,516
DPSO ≥200	0.00	0.00	0.00	0.00	1.00	1,866	3,077
IRS	1.41	0.28	0.85	1.96	1.00	350	519
Antigen: EDIII	−1.42	0.31	−2.02	−0.79	1.01	370	654
Antigen: NS1	−2.59	0.33	−3.24	−1.92	1.01	291	506
Virus: DV2-XR	−0.29	0.34	−0.95	0.40	1.01	430	696
Virus: DV4-XR	−0.99	0.34	−1.69	−0.33	1.01	383	712
Virus: ZV-XR	−1.76	0.35	−2.43	−1.02	1.01	435	626
Ab isotype/subclass: IgG	2.57	0.43	1.71	3.37	1.01	441	559
Ab isotype/subclass: IgG1	2.07	0.42	1.22	2.87	1.01	390	827
Ab isotype/subclass: IgG2	−0.78	0.40	−1.57	0.02	1.01	412	953
Ab isotype/subclass: IgG3	−1.18	0.42	−2.02	−0.39	1.02	337	646
Ab isotype/subclass: IgG4	−3.09	0.41	−3.87	−2.29	1.02	298	708
Ab isotype/subclass: IgM	0.65	0.42	−0.17	1.49	1.01	405	798
Age	0.02	0.02	−0.01	0.06	1.01	528	1,032
Sex (Male)	−0.20	0.09	−0.38	−0.02	1.00	432	996

**Posterior mean estimates, standard errors (SE), and 95% credible intervals (CI) are shown for population-level fixed effects in the Bayesian model of $\log_2$ MFI. Days post-symptom onset (DPSO) was modeled using three piecewise linear spline segments (0–100, 100–200, and ≥200 days). Fixed effects represent average effects across antibody compartments, with uncertainty propagated through the multilevel structure. Convergence diagnostics ($\hat{R}$, bulk and tail effective sample sizes) are reported for all parameters.*

B. Time segment × covariate interaction effects**

Interaction Term	Estimate	SE	95% CI (Lower)	95% CI (Upper)	R̂	Bulk ESS	Tail ESS
DPSO 0–100 × IRS	−0.01	0.00	−0.01	−0.00	1.01	402	766
DPSO 0–100 × EDIII	−0.00	0.00	−0.01	0.00	1.01	440	579
DPSO 0–100 × NS1	0.01	0.00	0.00	0.01	1.01	346	513
DPSO 0–100 × IgG	0.02	0.00	0.01	0.03	1.01	410	706
DPSO 0–100 × IgG1	0.02	0.00	0.01	0.03	1.00	474	775
DPSO 0–100 × IgG2	0.01	0.00	0.00	0.02	1.01	444	972
DPSO 100–200 × IgG	−0.01	0.00	−0.01	−0.00	1.00	1,285	2,691
DPSO 100–200 × IgG1	−0.01	0.00	−0.01	−0.01	1.00	1,406	2,456
DPSO 100–200 × IgG2	−0.01	0.00	−0.01	−0.01	1.00	1,255	2,359
DPSO ≥200 × IgG	−0.00	0.00	−0.00	0.00	1.00	1,729	3,028
DPSO ≥200 × IgG1	−0.00	0.00	−0.00	0.00	1.00	1,903	3,158
DPSO ≥200 × IgG3	−0.00	0.00	−0.00	−0.00	1.00	1,594	2,594
DPSO ≥200 × IgM	−0.00	0.00	−0.00	0.00	1.00	1,693	2,494

**Time segment × covariate interaction terms quantify differential antibody kinetics across Days post-symptom onset (DPSO) spline intervals. Estimates are reported on the $\log_2$ MFI scale with 95% credible intervals. These parameters support the spline-based structure of the model and are provided for completeness and transparency.*

C. Residual variance parameter*

Parameter	Estimate	SE	95% CI (Lower)	95% CI (Upper)	R̂	Bulk ESS	Tail ESS
Residual SD (σ)*	0.91	0.00	0.90	0.92	1.00	4,012	3,127

*The residual standard deviation (σ) represents unexplained within-compartment variability on the $\log_2$ MFI scale.

Supplementary Figures

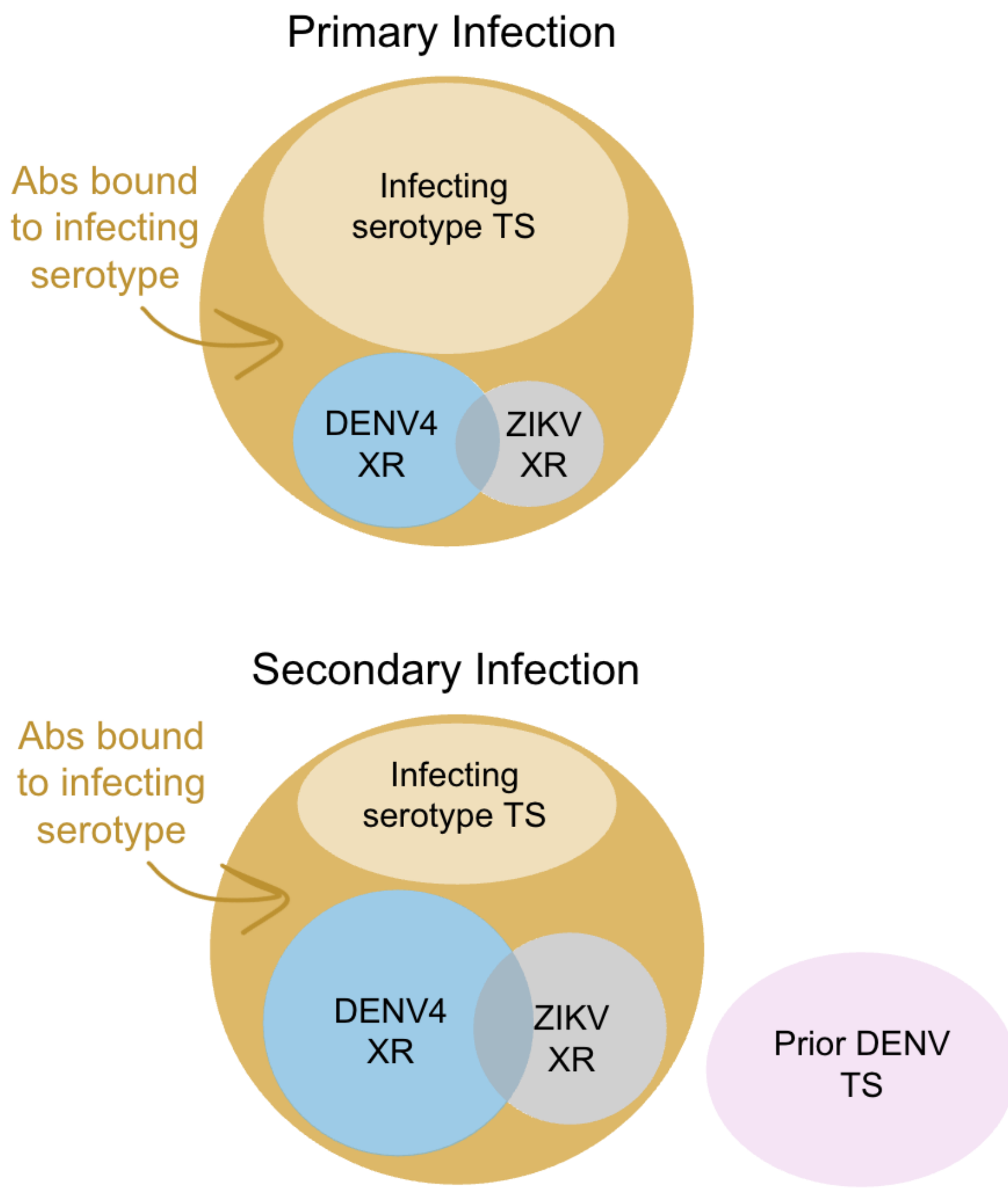

Supplementary Figure 1. Schematic representation of key concepts regarding cross-reactive antibody sub-populations during a primary versus a secondary anti-DENV immune response. Notably, antibodies (Abs) against the infecting serotype (i.e., homologous) do not represent only type-specific (TS) antibodies, which are not possible to measure with our approach. The size of the circles represents the magnitude of each cross-reactive (XR) subset as per our current understanding.

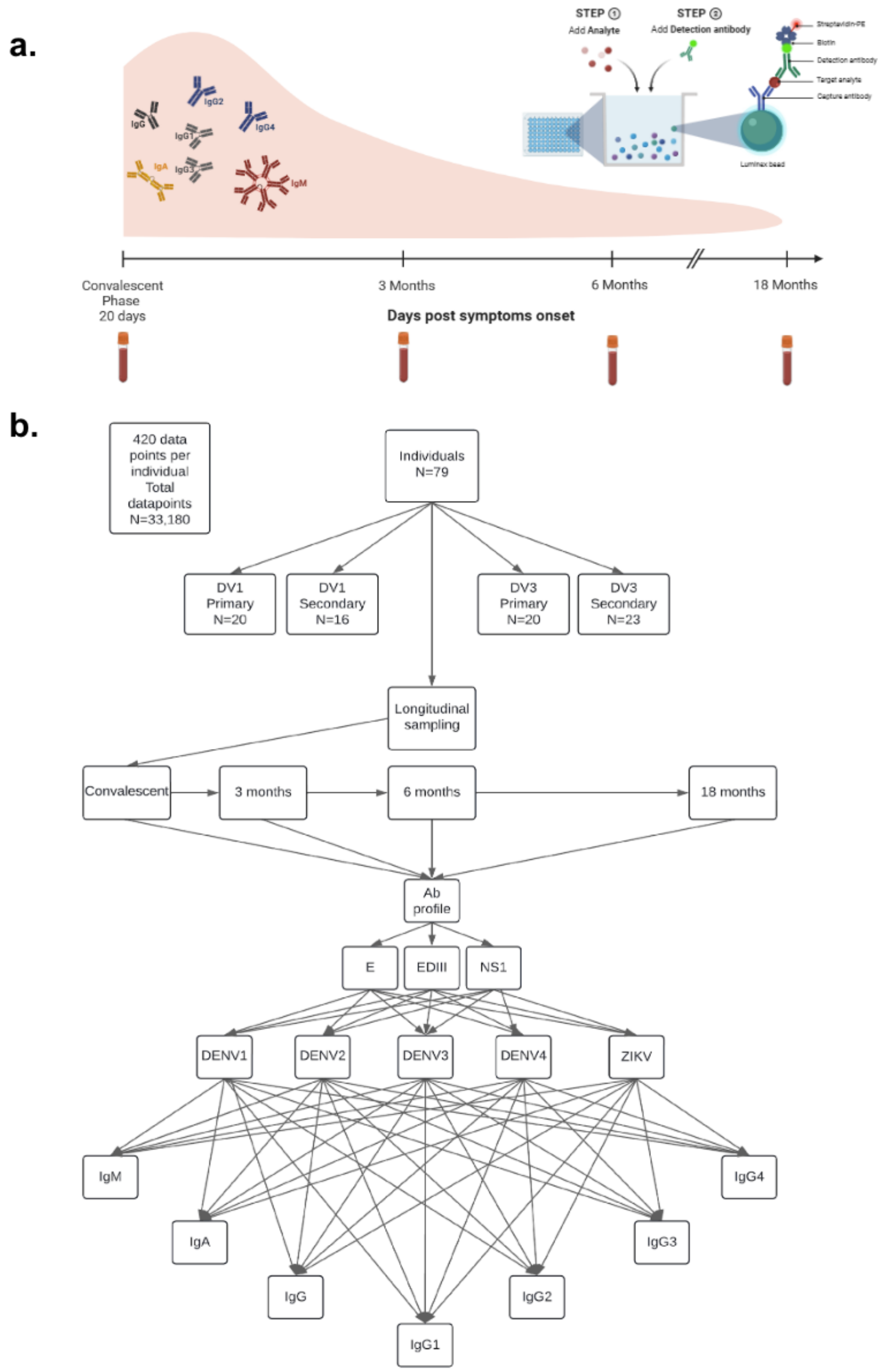

Supplementary Figure 2. Schematic of experimental and analysis approach.
a. The experimental approach utilized longitudinal plasma samples collected at early convalescence (14-28 days), 3 months, 6 months and 18 months post-onset of dengue symptoms. Antibody levels were measured using a multiplexed bead-based assay where the antigens were conjugated to magnetic fluorescent beads, co-incubated with plasma, and the isotype or IgG subclass or antigen-bound antibodies detected with a fluorescent secondary antibody. **b.** In total, 420 data points were collected for each of the 79 individuals, yielding 33,180 data points in total. Given the longitudinal sampling, there are multiple relationships between the key variables, which are indicated with arrows.

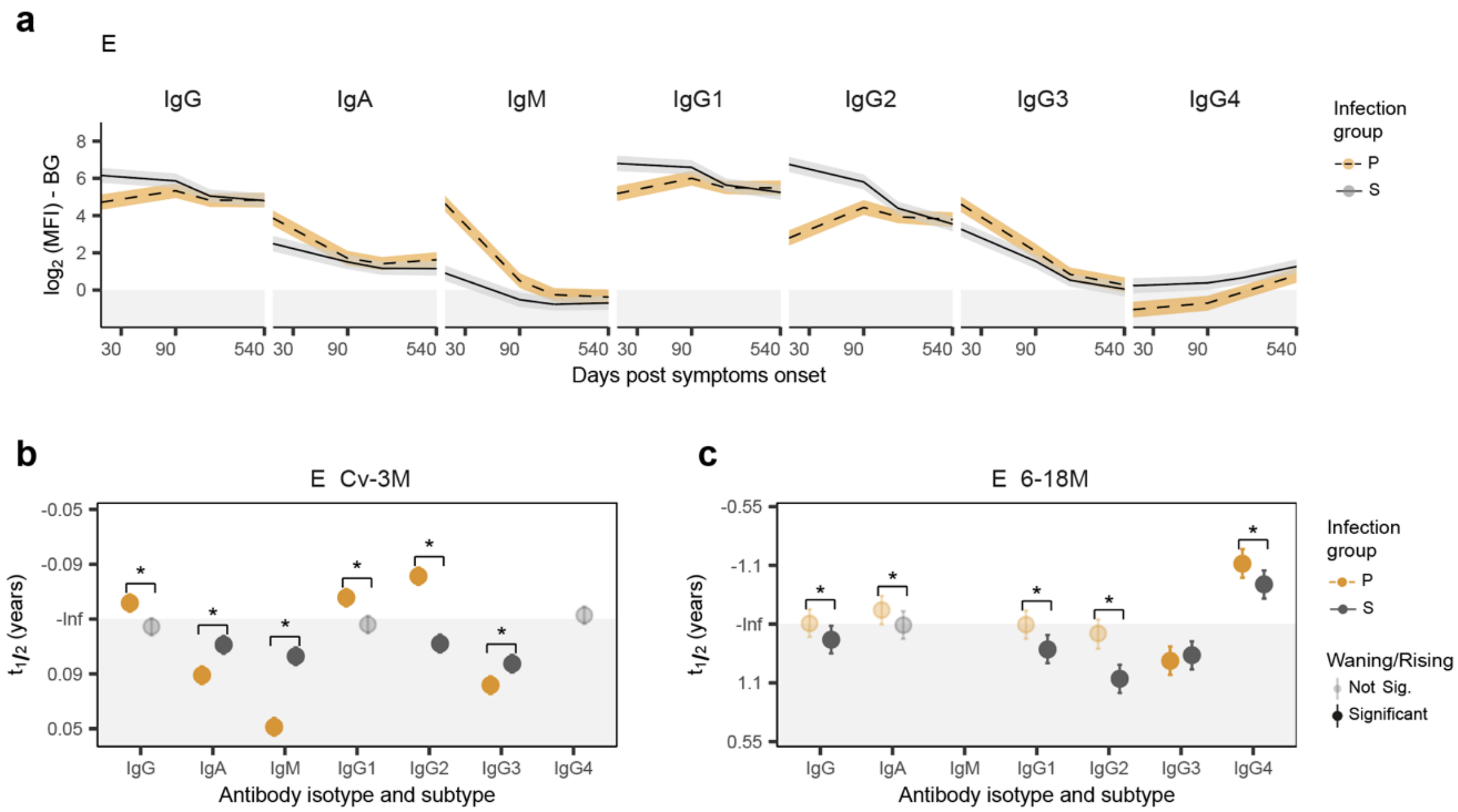

Supplementary Figure 3. Bayesian linear mixed-model analysis of homologous E-binding antibody responses after primary and secondary DENV infection.

E-binding antibodies against the homologous infecting serotype were measured as Log_2 of mean fluorescence intensity (MFI) after background subtraction (BG; shaded area). A Bayesian linear mixed model was applied to estimate decay for three time-period segments (Cv–3M, 3–6M, 6–18M; see Methods) (n = 39 post-primary; n = 39 post-secondary dengue cases). **a.** Homologous E-binding antibody levels over time of primary (P; dotted line, yellow) versus secondary (S; solid line, grey) dengue cases, for each isotype and IgG subclass. Lines represent the posterior mean and shaded bands the 95% credible interval (CI). **b, c.** Short-term antibody half-lives ($t_{1/2}$) estimated from Cv–3M antibody decay rates (**b**) and long-term $t_{1/2}$ estimated from 6–18M antibody decay rates (**c**). Points and error bars represent the posterior median and 95% credible interval. Transparent colors indicate stable decay rates (Not Sig.); solid colors are significantly rising or waning antibody kinetics. Asterisks denote significant differences between primary and secondary decay rates based on a credible-interval overlap criterion. For time segments in which the modelled trajectory remained at or below zero over the entire interval (a), the corresponding half-life estimates and CI were omitted from b and c. Source data are provided as a Source Data file.

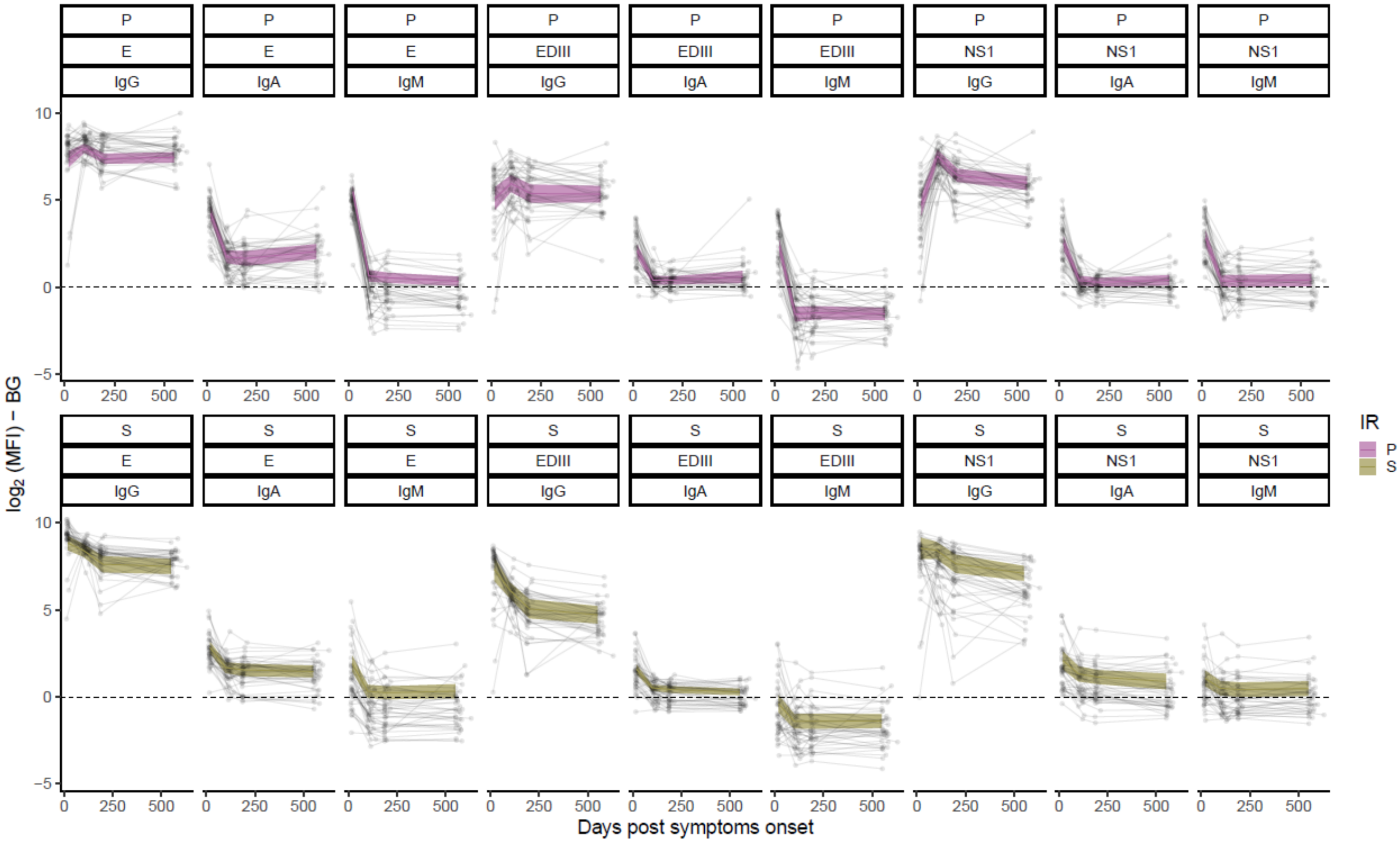

Supplementary Figure 4. Model fit atop individual antibody trajectories for homologous antibody responses against the infecting serotype.

The magnitude of binding antibodies was measured as Log_2 of mean fluorescence intensity (MFI) after background subtraction (BG; shaded area). A model was applied to estimate a linear rate of decay for three time-period segments: 30-90, >90-180, and >180-540 days post-symptom onset. Raw data of IgG, IgA, and IgM antibody levels against E, EDIII and NS1 over time for each individual with respect to antibody responses against the infecting serotype are shown. (n = 39 post-primary; n = 39 post-secondary dengue cases). Each thin grey line represents the antibody trajectory of one individual across timepoints; thick coloured lines represent the model-estimated mean fit. Model fit of the primary group is shown in pink (P) and the secondary group in green (S), with 95% confidence intervals. The dashed lines represents the background derived from DENV-naïve plasma samples.

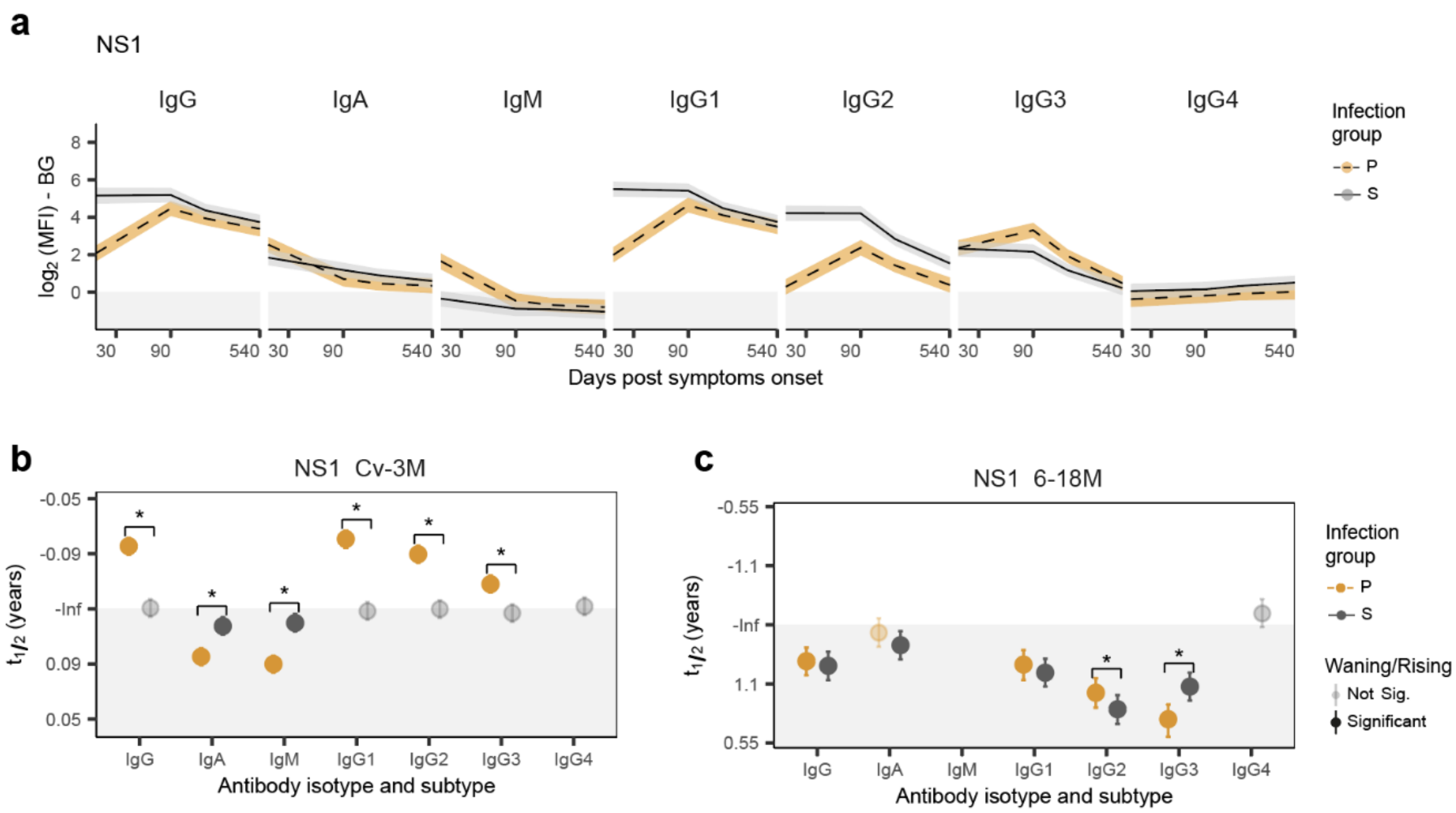

Supplementary Figure 5. Bayesian linear mixed-model analysis of homologous NS1-binding antibody responses after primary and secondary dengue.
NS1-binding antibodies against the homologous infecting serotype (DENV1 or DENV3) were measured as in Supplementary Figure 3 (n = 39 post-primary; n = 39 post-secondary dengue cases). **a.** Homologous DENV NS1-binding antibody levels over time of primary (P; dotted line, yellow) versus secondary (S; solid line, grey) dengue cases, for each isotype and IgG subclass. Lines represent the posterior mean and shaded bands the 95% credible interval (CI). **b, c.** Short-term antibody half-lives ($t_{1/2}$) estimated from Cv–3M antibody decay rates (**b**) and long-term $t_{1/2}$ estimated from 6–18M antibody decay rates (**c**). Points and error bars represent the posterior median and 95% credible interval. Transparent colors indicate stable decay rates (Not Sig.); solid colors are significantly rising or waning antibody kinetics. Asterisks denote significant differences between primary and secondary decay rates based on a credible-interval overlap criterion. For time segments in which the modelled trajectory remained at or below zero over the entire interval (a), the corresponding half-life estimates and CI were omitted from b and c. Source data are provided as a Source Data file.

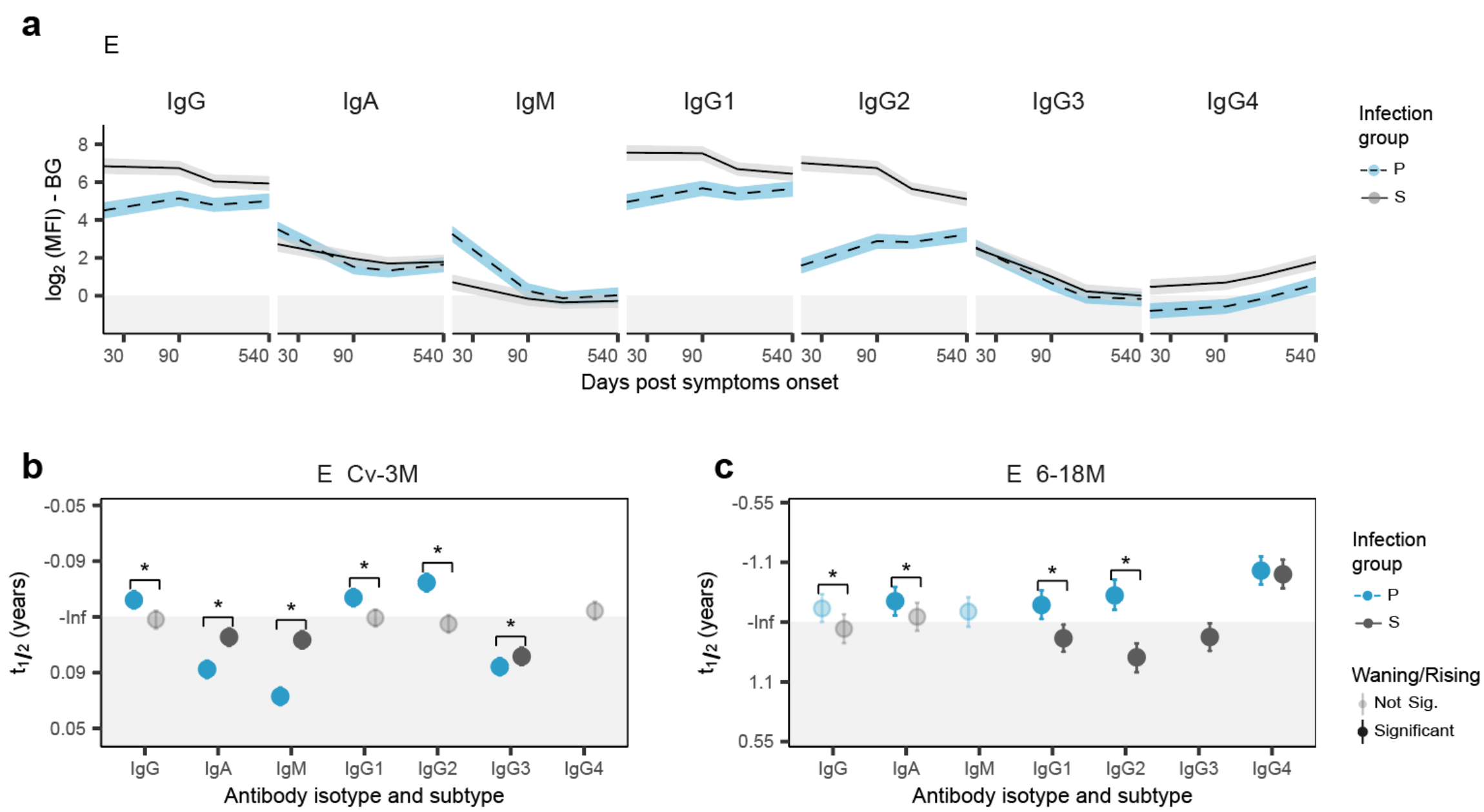

Supplementary Figure 6. Bayesian linear mixed-model analysis of DENV serocomplex cross-reactive anti-E antibody responses after primary and secondary dengue.
Cross-reactive (XR) E-binding antibodies were assessed as in Supplementary Figure 3, against the DENV4 serotype, which was infrequent in our cohort during the time-period of infections sampled here(n = 39 post-primary; n = 39 post-secondary dengue cases. **a.** XR E-binding antibody levels over time of primary (P; dotted line, blue) versus secondary (S; solid line, grey) dengue cases, for each isotype and IgG subclass. Lines represent the posterior mean and shaded bands the 95% credible interval (CI). **b, c.** Short-term antibody half-lives ($t_{1/2}$) estimated from Cv–3M antibody decay rates (**b**) and long-term $t_{1/2}$ estimated from 6–18M antibody decay rates (**c**). Points and error bars represent the posterior median and 95% credible interval. Transparent colors indicate stable decay rates (Not Sig.); solid colors are significantly rising or waning antibody kinetics. Asterisks denote significant differences between primary and secondary decay rates based on a credible-interval overlap criterion. For time segments in which the modelled trajectory remained at or below zero over the entire interval (a), the corresponding half-life estimates and CI were omitted from b and c. Source data are provided as a Source Data file.

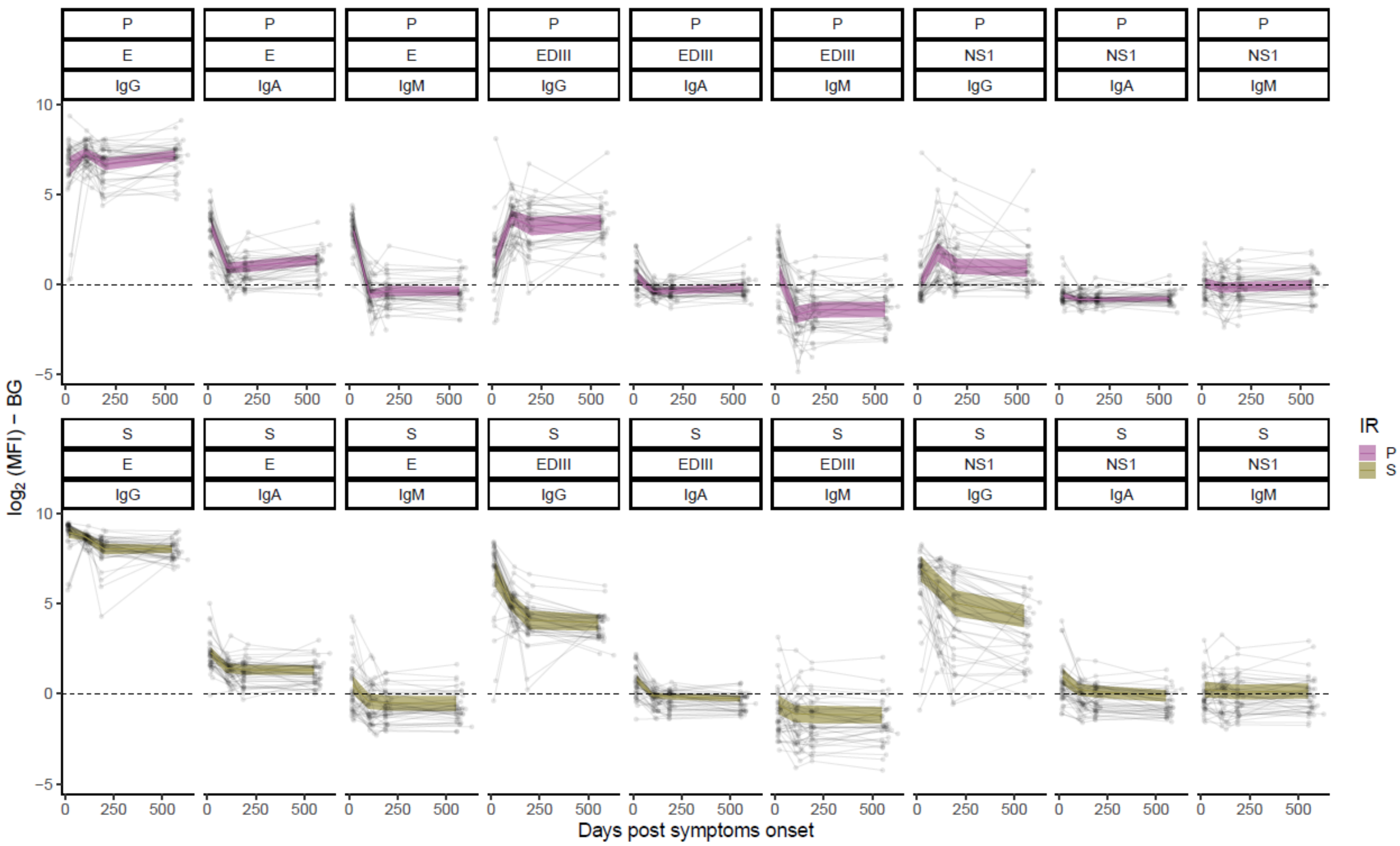

Supplementary Figure 7. Model fit atop individual antibody trajectories for antibody responses against the cross-reactive DENV4 serotype.

The magnitude of binding antibodies was measured as Log_2 of mean fluorescence intensity (MFI) after background subtraction (BG; shaded area). A model was applied to estimate a linear rate of decay for three time-period segments: 30-90, >90-180, and >180-540 days post-symptom onset. Raw data on IgG, IgA, and IgM antibody levels against E, EDIII and NS1 over time for each individual with respect to antibody responses against the DENV4 serotype are shown. (n = 39 post-primary; n = 39 post-secondary dengue cases). Each thin grey line represents the antibody trajectory of one individual across timepoints; thick coloured lines represent the model-estimated mean fit. Model fit of the primary group is shown in pink (P) and the secondary group in green (S), with 95% confidence intervals. The dashed lines represent the background derived from DENV-naïve plasma samples.

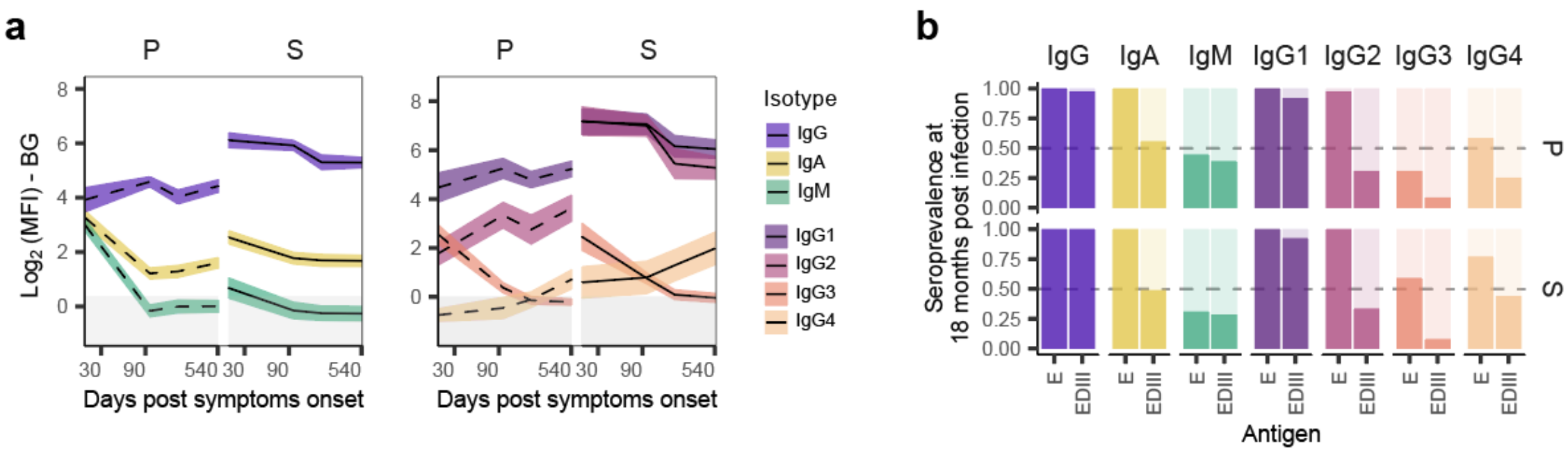

Supplementary Figure 8. Cross-reactive antibody responses against DENV4 E protein.
Plasma antibodies against the cross-reactive DENV4 E protein were measured as Log_2 of mean fluorescence intensity (MFI) after background subtraction (BG; shaded area) (n = 39 post-primary; n = 39 post-secondary dengue cases for the LMM in panel a; n = 36 post-primary; n = 39 post-secondary for the 18-month observed seroprevalence in panel b). **a.** Comparison of DENV4 E-binding antibody trajectories of primary (P; dotted line) versus secondary (S; solid line) dengue cases, with isotypes (IgG, IgA, IgM) and IgG subclasses (IgG1–4) overlaid. Lines represent the model-estimated mean and shaded bands the 95% bootstrap confidence interval; colors indicate antibody isotype/subclass (legend). **b.** Seroprevalence of DENV4 E-binding antibodies in the primary versus secondary dengue group at 18 months post-symptom onset, with dashed line at 50%. Bars represent the percentage of participants seropositive in each group, calculated from observed 18-month values. Source data are provided as a Source Data file.

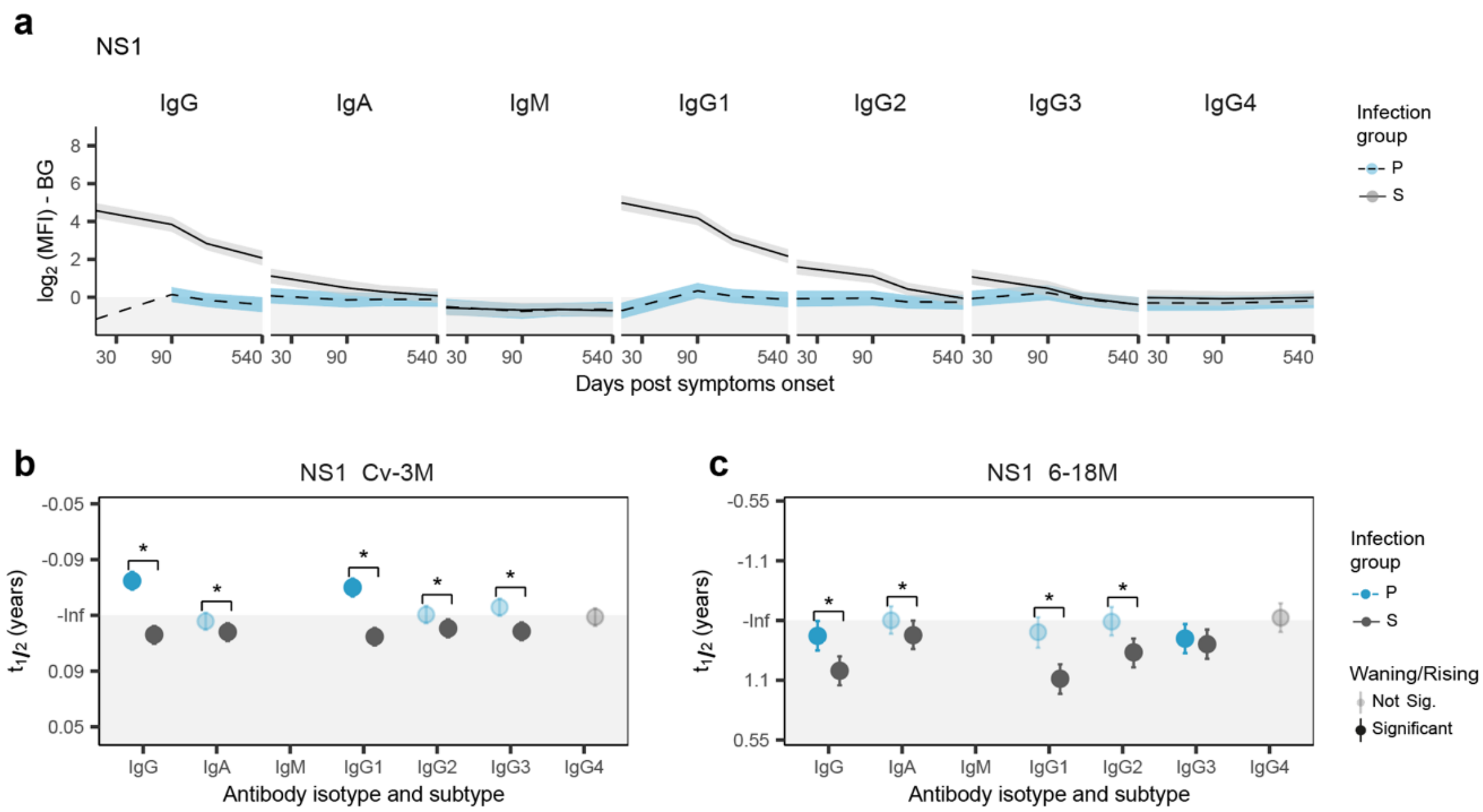

Supplementary Figure 9. Bayesian linear mixed-model analysis of DENV serocomplex cross-reactive anti-NS1 antibody responses after primary and secondary dengue.

NS1-binding plasma antibodies against the DENV4 serotype were measured as Log_2 of mean fluorescence intensity (MFI) after background subtraction (BG; shaded area). A Bayesian linear mixed model was applied to estimate decay for three time-period segments (Cv–3M, 3–6M, 6–18M; see Methods) (n = 39 post-primary; n = 39 post-secondary dengue cases). **a.** XR NS1-binding antibody levels over time of primary (P; dotted line, blue) versus secondary (S; solid line, grey) dengue cases, for each isotype and IgG subclass. Lines represent the posterior mean and shaded bands the 95% credible interval (CI). **b, c.** Short-term antibody half-lives ($t_{1/2}$) estimated from Cv–3M antibody decay rates (**b**) and long-term $t_{1/2}$ estimated from 6–18M antibody decay rates (**c**). Points and error bars represent the posterior median and 95% credible interval. Transparent colors indicate stable decay rates (Not Sig.); solid colors are significantly rising or waning antibody kinetics. Asterisks denote significant differences between primary and secondary decay rates based on a credible-interval overlap criterion. For time segments in which the modelled trajectory remained at or below zero over the entire interval (a), the corresponding half-life estimates and CI were omitted from b and c. Source data are provided as a Source Data file.

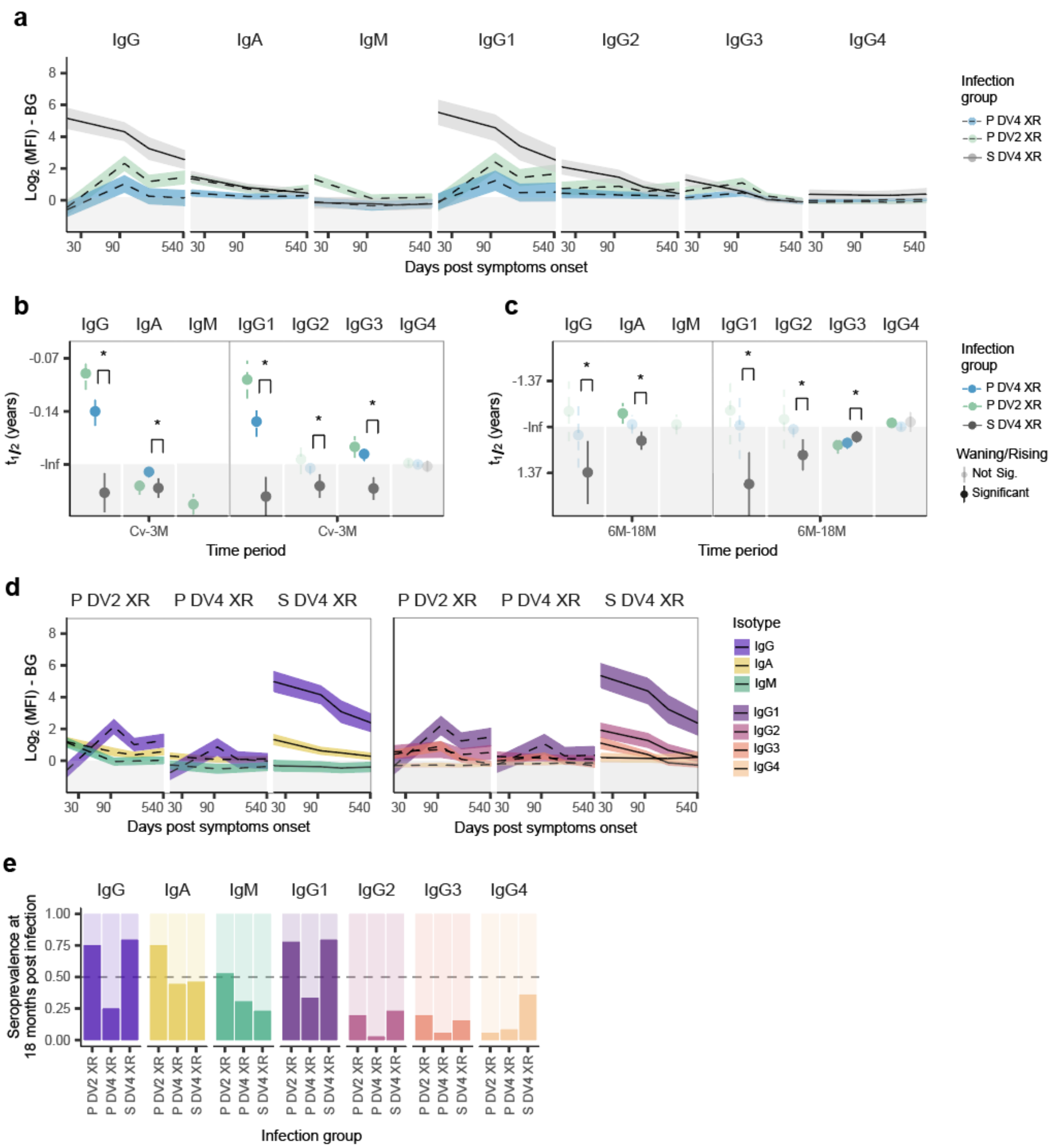

Supplementary Figure 10. DENV serocomplex cross-reactive anti-NS1 antibodies are stable after primary dengue but wane post-secondary dengue.

Cross-reactive (XR) NS1-binding plasma antibodies from DENV1 and DENV3 infections were measured against the DENV2 and DENV4 serotypes as Log_2 of mean fluorescence intensity (MFI) after background subtraction (BG; shaded area). A linear mixed-effects model with bootstrapping was applied to estimate a linear rate of decay for three time-period segments (Cv–3M, 3–6M, 6–18M; see Methods) (n = 39 post-primary; n = 39 post-secondary dengue cases for a–d; n = 36 post-primary; n = 39 post-secondary for panel e). **a.** DENV2 and DENV4 XR NS1-binding antibody levels of primary (P; dotted line, blue and green) versus DENV4 XR NS1-binding antibody levels of secondary (S; solid line, grey) dengue cases over time, for each isotype and IgG subclass. Lines represent the model-estimated mean and shaded bands the 95% bootstrap confidence interval. **b, c.** Short-term antibody half-lives (**b**) and long-term half-lives (**c**). Points and error bars represent the bootstrap mean and 95% confidence interval. Transparent colors indicate stable decay rates (Not Sig.); solid colors are significantly rising or waning antibody kinetics. Asterisks denote significant differences based on a confidence-interval overlap criterion. For time segments in which the modelled trajectory remained at or below zero over the entire interval (a), the corresponding half-life estimates and CI were omitted from b and c. **d.** Comparison of DENV2 and DENV4 XR NS1-binding antibody trajectories, with isotypes (IgG, IgA, IgM) and IgG subclasses (IgG1–4) overlaid; three columns

correspond to P DENV2 XR, P DENV4 XR, and S DENV4 XR. Lines represent the model-estimated mean and shaded bands the 95% bootstrap confidence interval; colors indicate antibody isotype/subclass (legend). **e.** Seroprevalence of XR NS1-binding antibodies in the primary versus secondary dengue group at 18 months post-symptom onset, with dashed line at 50%. Bars represent the percentage of participants seropositive, calculated from observed 18-month values. Source data are provided as a Source Data file.

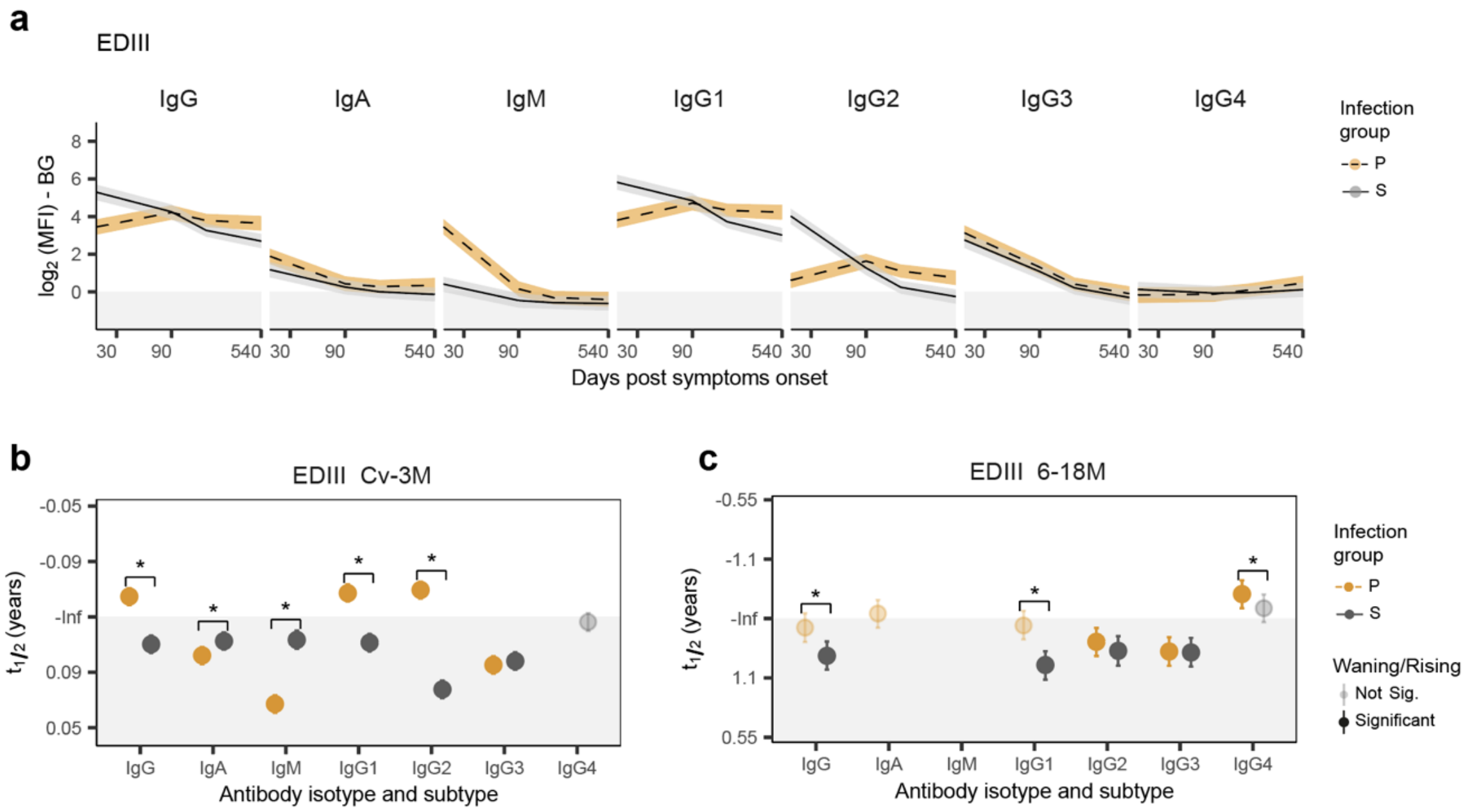

Supplementary Figure 11. Bayesian linear mixed-model analysis of homologous EDIII-binding antibody responses after primary and secondary dengue.

EDIII-binding plasma antibodies against the homologous infecting serotype were measured as Log_2 of mean fluorescence intensity (MFI) after background subtraction (BG; shaded area). A Bayesian linear mixed model was applied to estimate decay for three time-period segments (Cv–3M, 3–6M, 6–18M; see Methods) (n = 39 post-primary; n = 39 post-secondary dengue cases). **a.** Homologous EDIII-binding antibody levels over time of primary (P; dotted line, yellow) versus secondary (S; solid line, grey) dengue cases, for each isotype and IgG subclass. Lines represent the posterior mean and shaded bands the 95% credible interval (CI). **b, c.** Short-term antibody half-lives ($t_{1/2}$) estimated from Cv–3M antibody decay rates (**b**) and long-term $t_{1/2}$ estimated from 6–18M antibody decay rates (**c**). Points and error bars represent the posterior median and 95% credible interval. Transparent colors indicate stable decay rates (Not Sig.); solid colors are significantly rising or waning antibody kinetics. Asterisks denote significant differences between primary and secondary decay rates based on a credible-interval overlap criterion. For time segments in which the modelled trajectory remained at or below zero over the entire interval (a), the corresponding half-life estimates and CI were omitted from b and c. Source data are provided as a Source Data file.

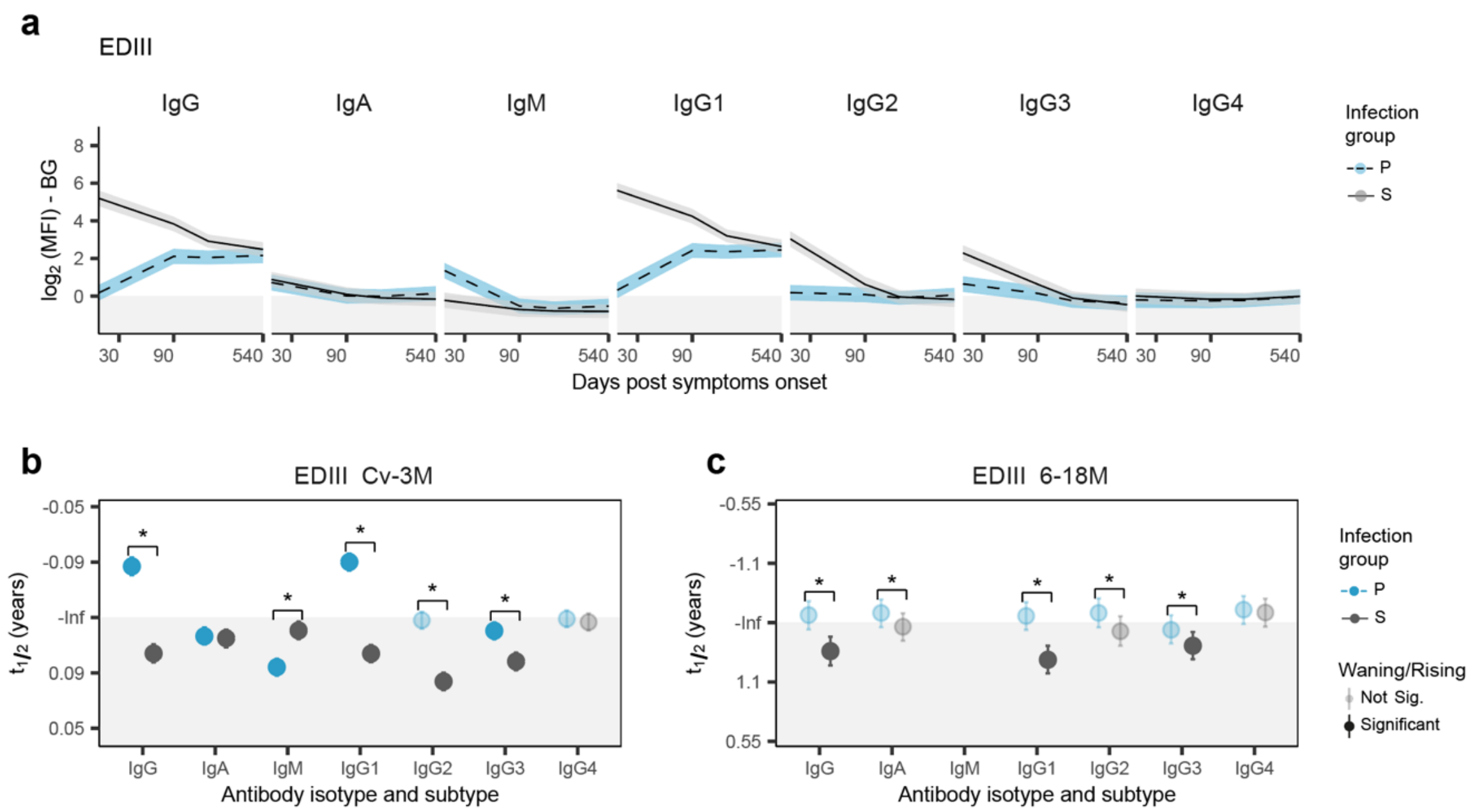

Supplementary Figure 12. Bayesian linear mixed-model analysis of DENV serocomplex cross-reactive anti-EDIII antibody responses after primary and secondary dengue.
EDIII-binding plasma antibodies against the DENV4 serotype were measured as Log_2 of mean fluorescence intensity (MFI) after background subtraction (BG; shaded area). A Bayesian linear mixed model was applied to estimate decay for three time-period segments (Cv–3M, 3–6M, 6–18M; see Methods) (n = 39 post-primary; n = 39 post-secondary dengue cases). **a.** XR EDIII-binding antibody levels over time of primary (P; dotted line, blue) versus secondary (S; solid line, grey) dengue cases, for each isotype and IgG subclass. Lines represent the posterior mean and shaded bands the 95% credible interval (CI**). b, c.** Short-term antibody half-lives ($t_{1/2}$) estimated from Cv–3M antibody decay rates (**b**) and long-term $t_{1/2}$ estimated from 6–18M antibody decay rates (**c**). Points and error bars represent the posterior median and 95% credible interval. Transparent colors indicate stable decay rates (Not Sig.); solid colors are significantly rising or waning antibody kinetics. Asterisks denote significant differences between primary and secondary decay rates based on a credible-interval overlap criterion. For time segments in which the modelled trajectory remained at or below zero over the entire interval (a), the corresponding half-life estimates and CI were omitted from b and c. Source data are provided as a Source Data file.

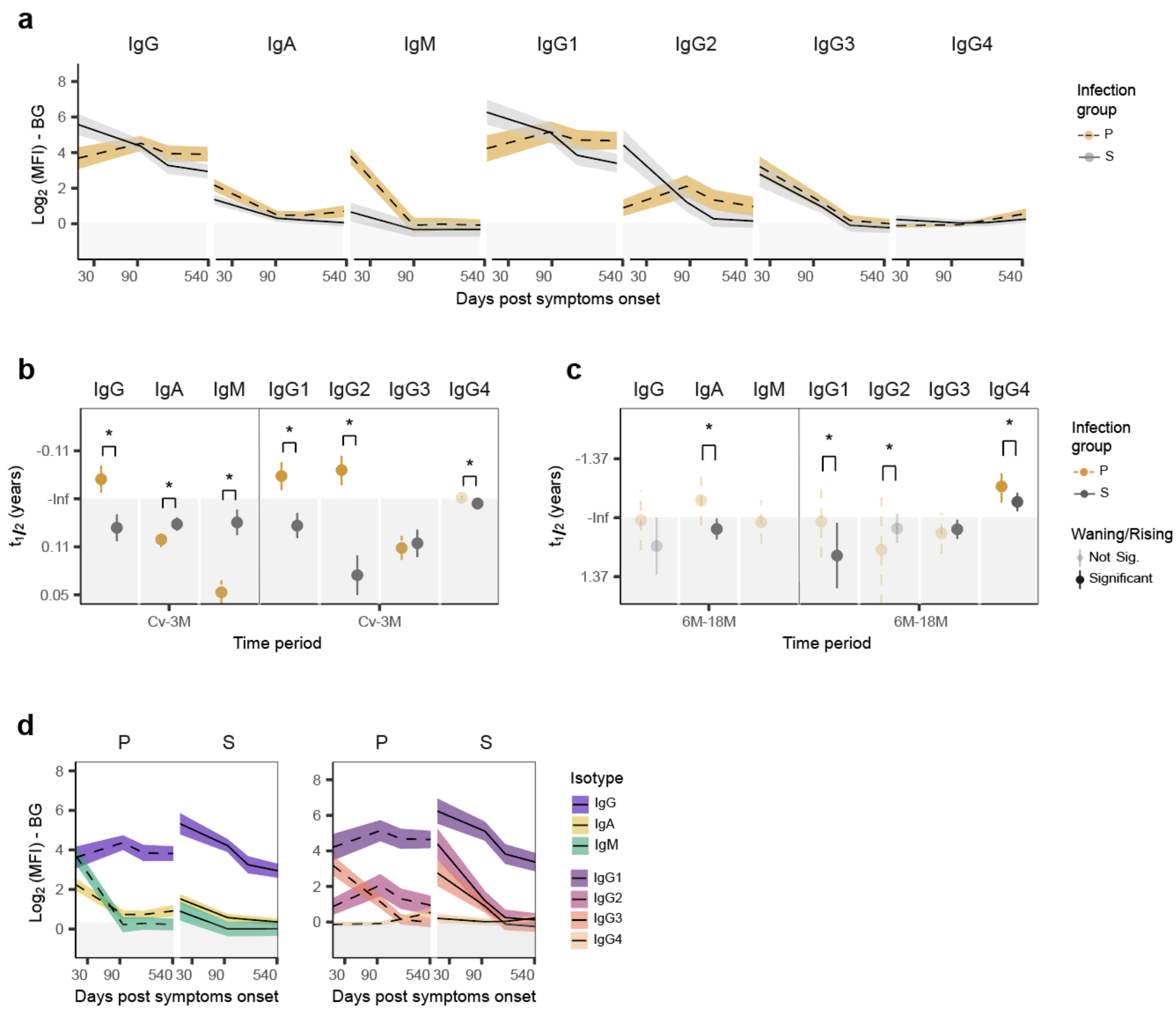

Supplementary Figure 13. Antibody responses against homologous EDIII protein after primary and secondary dengue.

EDIII-binding plasma antibodies against the homologous infecting serotype of either DENV1 or DENV3 were measured as Log_2 of mean fluorescence intensity (MFI) after background subtraction (BG; shaded area). A linear mixed-effects model with bootstrapping was applied to estimate a linear rate of decay for three time-period segments (Cv–3M, 3–6M, 6–18M; see Methods) (n = 39 post-primary; n = 39 post-secondary dengue cases). **a.** Homologous EDIII-binding antibody levels over time of primary (P; dotted line, yellow) versus secondary (S; solid line, grey) dengue cases, for each isotype and IgG subclass. Lines represent the model-estimated mean and shaded bands the 95% bootstrap confidence interval. **b, c.** Short-term antibody half-lives ($t_{1/2}$) estimated from Cv–3M antibody decay rates (**b**) and long-term $t_{1/2}$ estimated from 6–18M antibody decay rates (**c**). Points and error bars represent the bootstrap mean and 95% confidence interval. Transparent colors indicate stable decay rates (Not Sig.); solid colors are significantly rising or waning antibody kinetics. Asterisks denote significant differences between primary and secondary decay rates based on a confidence-interval overlap criterion. For time segments in which the modelled trajectory remained at or below zero over the entire interval (a), the corresponding half-life estimates and CI were omitted from b and c. **d.** Comparison of homologous EDIII-binding antibody trajectories of primary (P; dotted line) versus secondary (S; solid line) dengue cases, with isotypes (IgG, IgA, IgM) and IgG subclasses (IgG1–4) overlaid. Lines represent the model-estimated mean and shaded bands the 95% bootstrap confidence interval; colors indicate antibody isotype/subclass (legend). Source data are provided as a Source Data file.

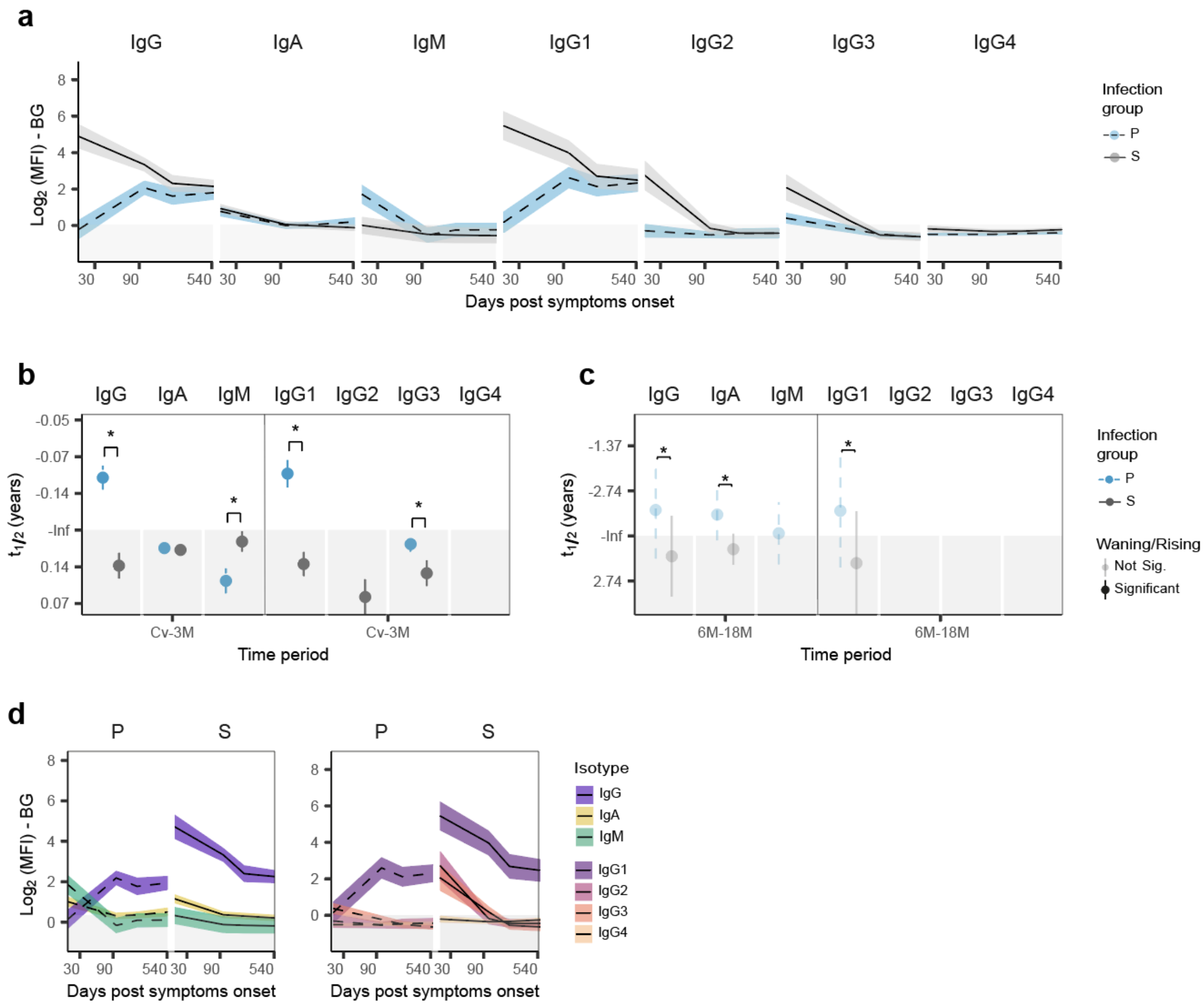

Supplementary Figure 14. Antibody responses against cross-reactive EDIII protein after primary and secondary dengue.

Cross-reactive EDIII-binding plasma antibodies against the DENV4 serotype were measured as Log_2 of mean fluorescence intensity (MFI) after background subtraction (BG; shaded area). A linear mixed-effects model with bootstrapping was applied to estimate a linear rate of decay for three time-period segments (Cv–3M, 3–6M, 6–18M; see Methods) (n = 39 post-primary; n = 39 post-secondary dengue cases). **a.** Cross-reactive EDIII-binding antibody levels over time of primary (P; dotted line, blue) versus secondary (S; solid line, grey) dengue cases, for each isotype and IgG subclass. Lines represent the model-estimated mean and shaded bands the 95% bootstrap confidence interval. **b, c.** Short-term antibody half-lives ($t_{1/2}$) estimated from Cv–3M antibody decay rates (**b**) and long-term $t_{1/2}$ estimated from 6–18M antibody decay rates (**c**). Points and error bars represent the bootstrap mean and 95% confidence interval**.** Transparent colors indicate stable decay rates (Not Sig.); solid colors are significantly rising or waning antibody kinetics. Asterisks denote significant differences between primary and secondary decay rates based on a confidence-interval overlap criterion. For time segments in which the modelled trajectory remained at or below zero over the entire interval (a), the corresponding half-life estimates and CI were omitted from b and c. **d.** Comparison of cross-reactive EDIII-binding antibody trajectories of primary (P; dotted line) versus secondary (S; solid line) dengue cases, with isotypes (IgG, IgA, IgM) and IgG subclasses (IgG1–4) overlaid. Lines represent the model-estimated mean and shaded bands the 95% bootstrap confidence interval; colors indicate antibody isotype/subclass (legend). Source data are provided as a Source Data file.

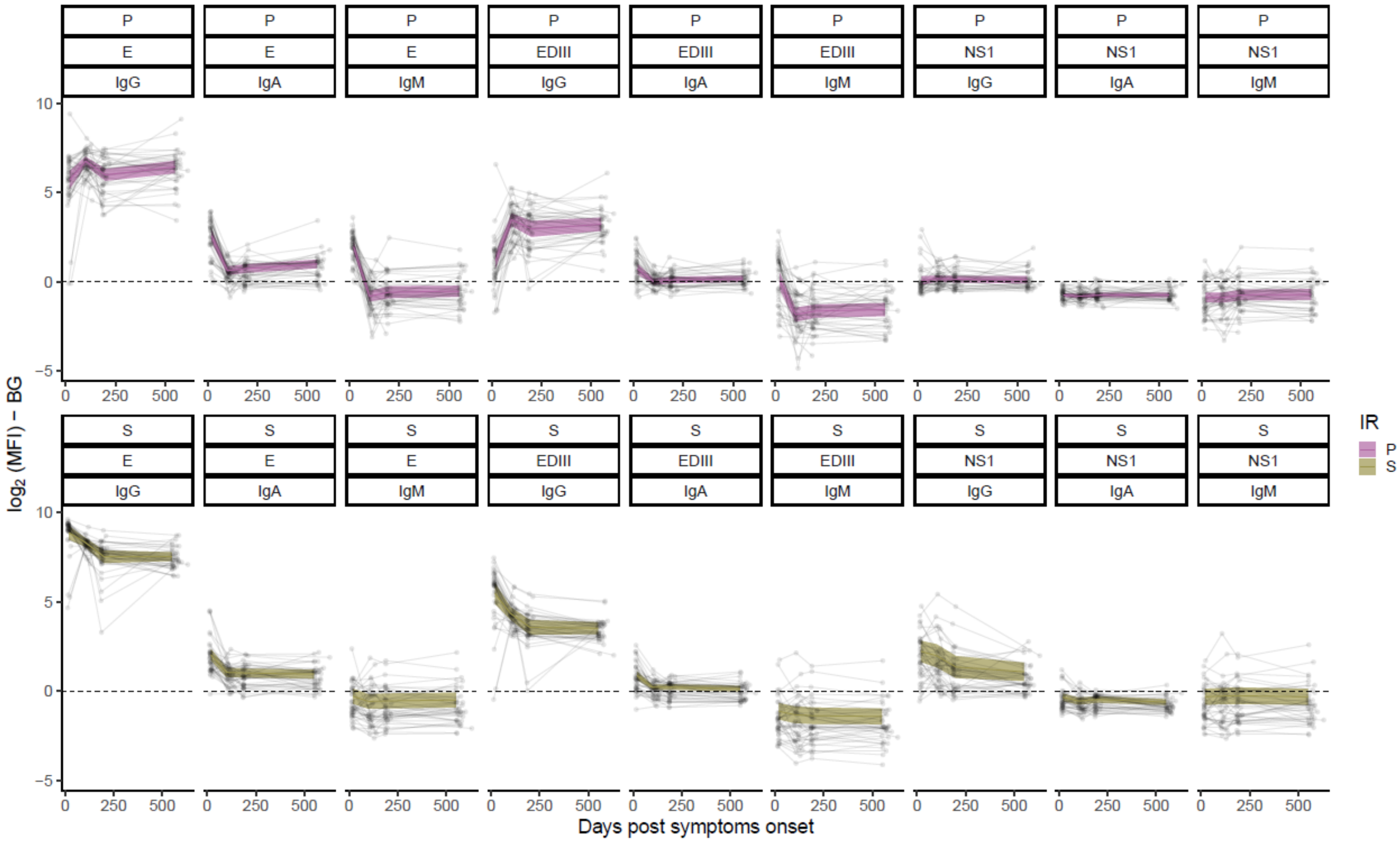

Supplementary Figure 15. Model fit atop individual antibody trajectories for cross-reactive antibody responses against ZIKV.

The magnitude of binding antibodies was measured as Log_2 of mean fluorescence intensity (MFI) after background subtraction (BG; shaded area). A model was applied to estimate a linear rate of decay for three time-period segments: 30-90, >90-180, and >180-540 days post-symptom onset. Raw data on IgG, IgA, and IgM antibody levels against E, EDIII and NS1 over time for each individual with respect to antibody responses against ZIKV are shown (n = 39 post-primary; n = 39 post-secondary dengue cases). Each thin grey line represents the antibody trajectory of one individual across timepoints; thick colored lines represent the model-estimated mean fit for the primary group (pink, P) and the secondary group (green, S), with shaded bands showing the 95% bootstrap confidence interval. Dashed lines represent the background derived from DENV-naïve plasma samples. Source data are provided as a Source Data file

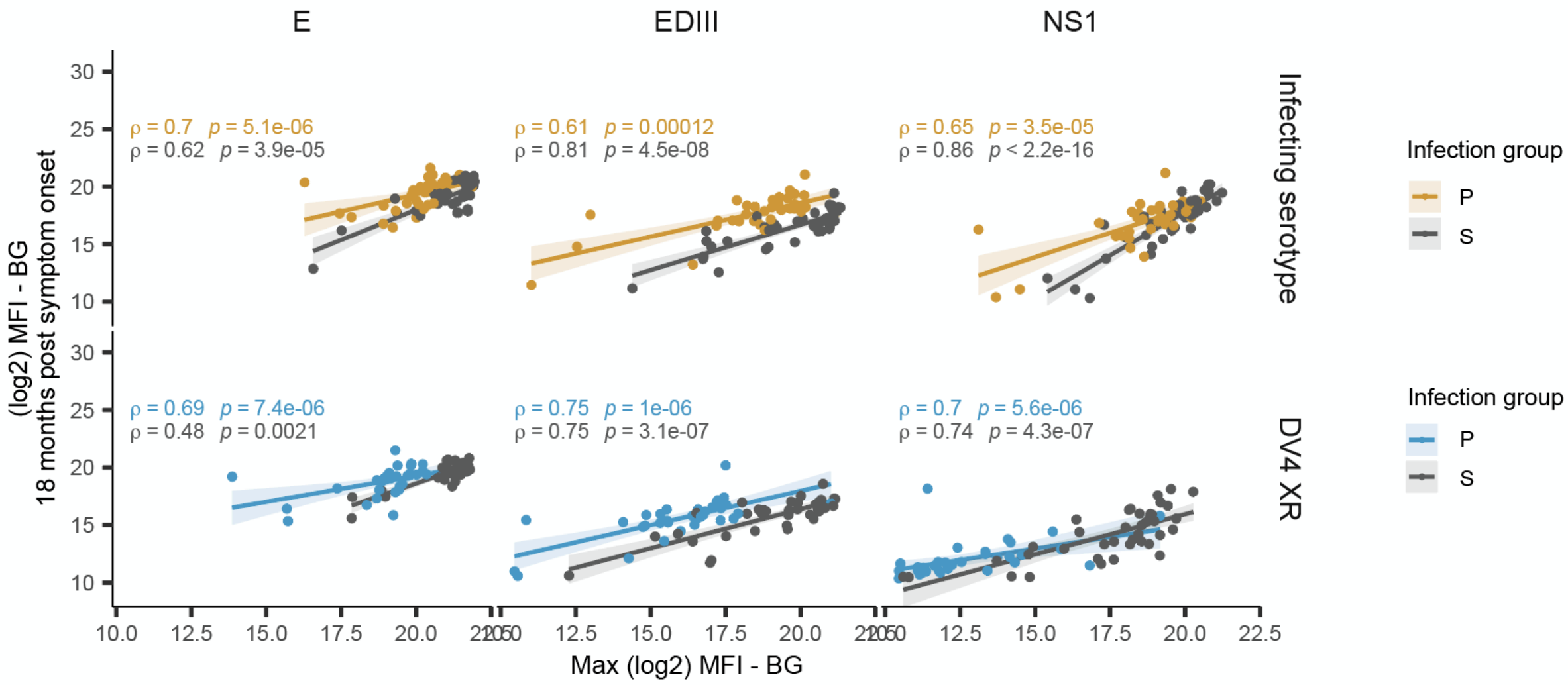

Supplementary Figure 16. Relationship between maximal observed early IgG1 antibody response and the level remaining at 18 months post-infection by antigen.

E-binding plasma IgG1 antibodies were measured as $\log_2$ of mean fluorescence intensity (MFI) after background subtraction (BG). Post-primary responses against the infecting serotype are in yellow, and post-primary responses against cross-reactive DENV4 serotype are in blue (DV4 XR). All post-secondary antibody responses are in grey. The maximal observed level of DENV-binding IgG1 (Max) was defined for each individual from samples collected up to 3 months post-symptom onset (n = 36 post-primary; n = 39 post-secondary dengue cases, observed values on both axes). The magnitude of this maximal early DENV-binding IgG1 was correlated with the magnitude of the same antibody subset at 18 months post-symptom onset. Spearman ρ and associated p-values are shown for each infection group. Each dot represents an individual. Axes were scaled to data for each antibody subset.

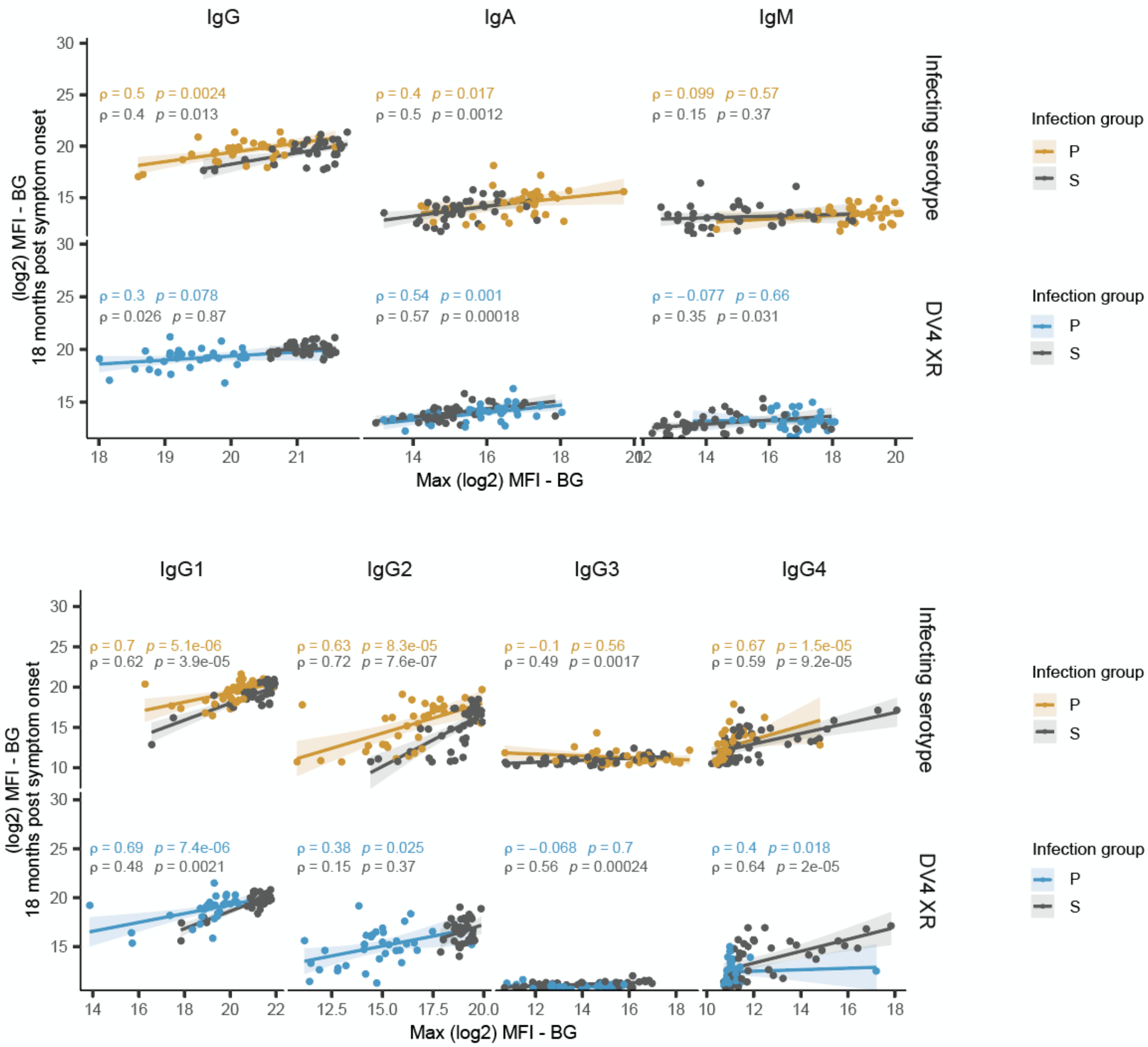

Supplementary Figure 17. High early antibody responses against E protein correlates with high antibody levels at 18 months post-infection for multiple antibody subsets.

E-binding plasma antibodies were measured as $\log_2$ of mean fluorescence intensity (MFI) after background subtraction (BG). Post-primary responses against the infecting serotype are in yellow, and post-primary responses against cross-reactive DENV4 serotype are in blue (DV4 XR). All post-secondary antibody responses are in grey. The maximal observed level of DENV-binding antibody (Max) was defined for each individual from samples collected up to 3 months post-symptom onset (n = 36 post-primary; n = 39 post-secondary dengue cases, observed values on both axes). The magnitude of this maximal early DENV-binding antibody was correlated with the magnitude of the same antibody subset at 18 months post-symptom onset (Spearman ρ and p-values shown for each infection group). Each dot represents an individual. Axes were scaled to data for each antibody subset.

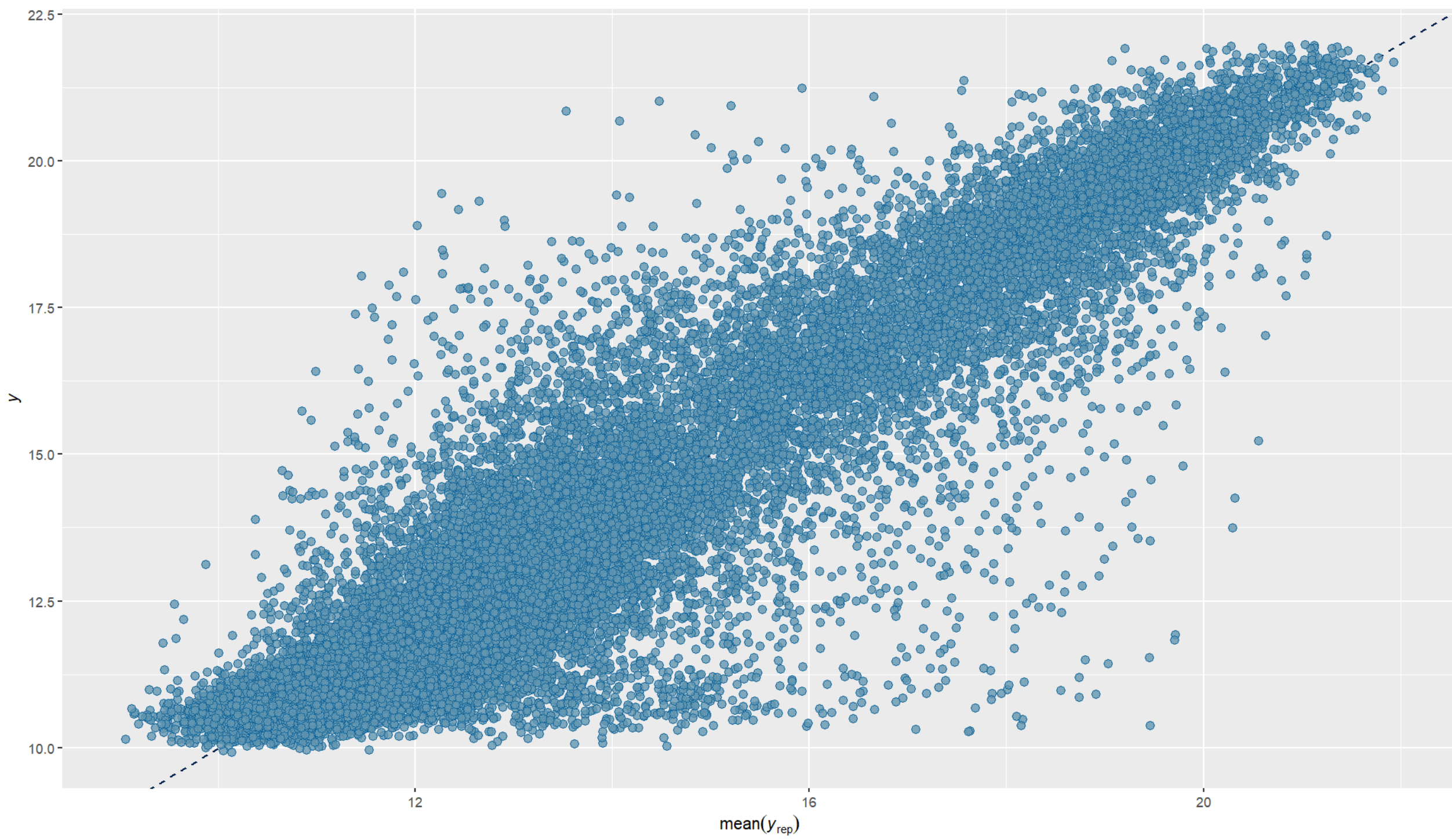

Supplementary Figure 18. Posterior predictive check of the Bayesian model.
Posterior predictive check comparing observed Log_2-transformed MFI values (y-axis) to posterior predictive means (mean(y_{rep}), x-axis) from the Bayesian hierarchical linear mixed model. Each point represents an individual observation. The dashed line indicates the identity line (y = x).

A. Baseline and time-spline fixed effects (intercept and DPSO segments)

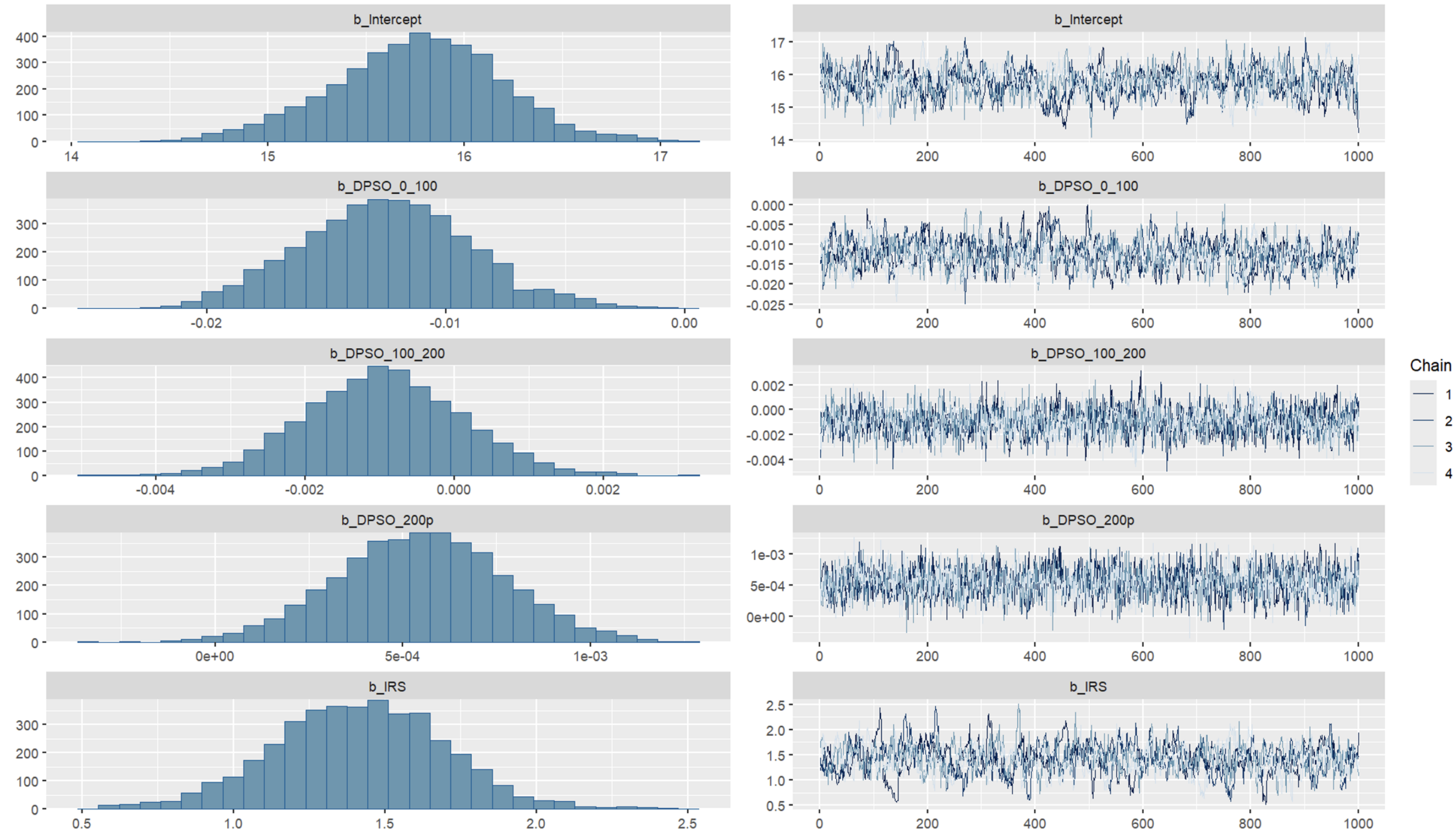

B. Antigen and antibody response type fixed effects

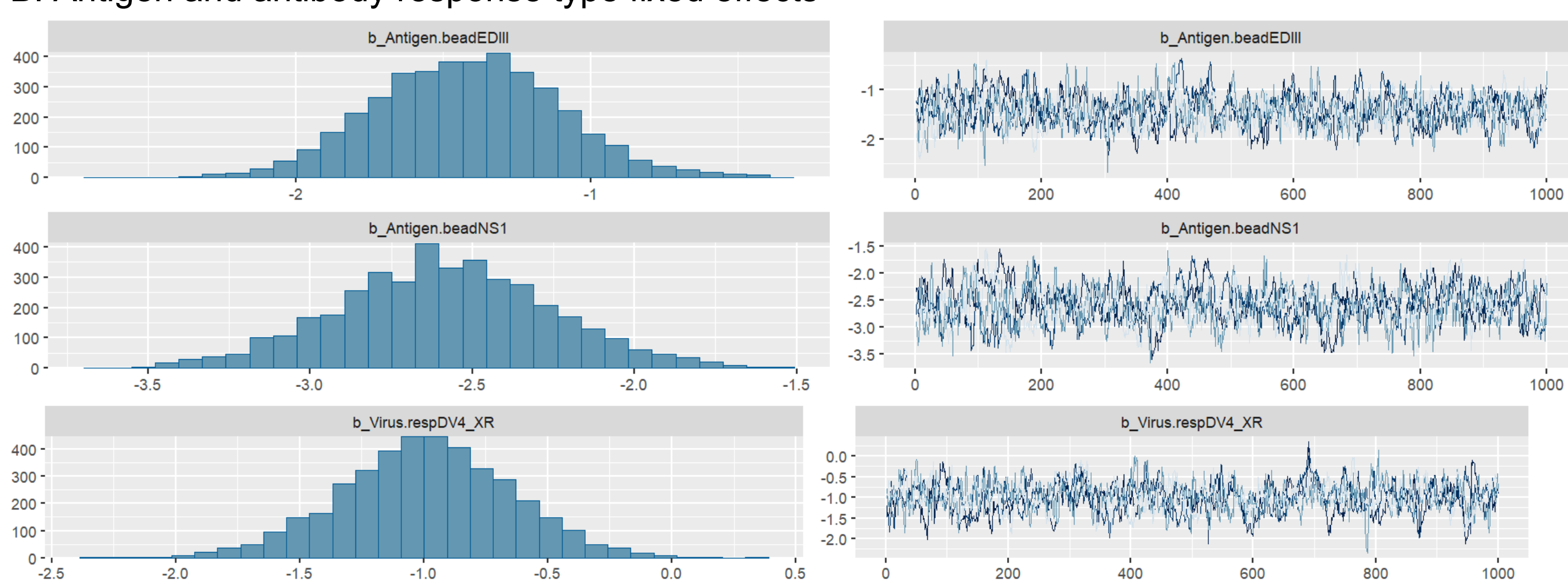

C. Infection group and isotype/subclass fixed effects

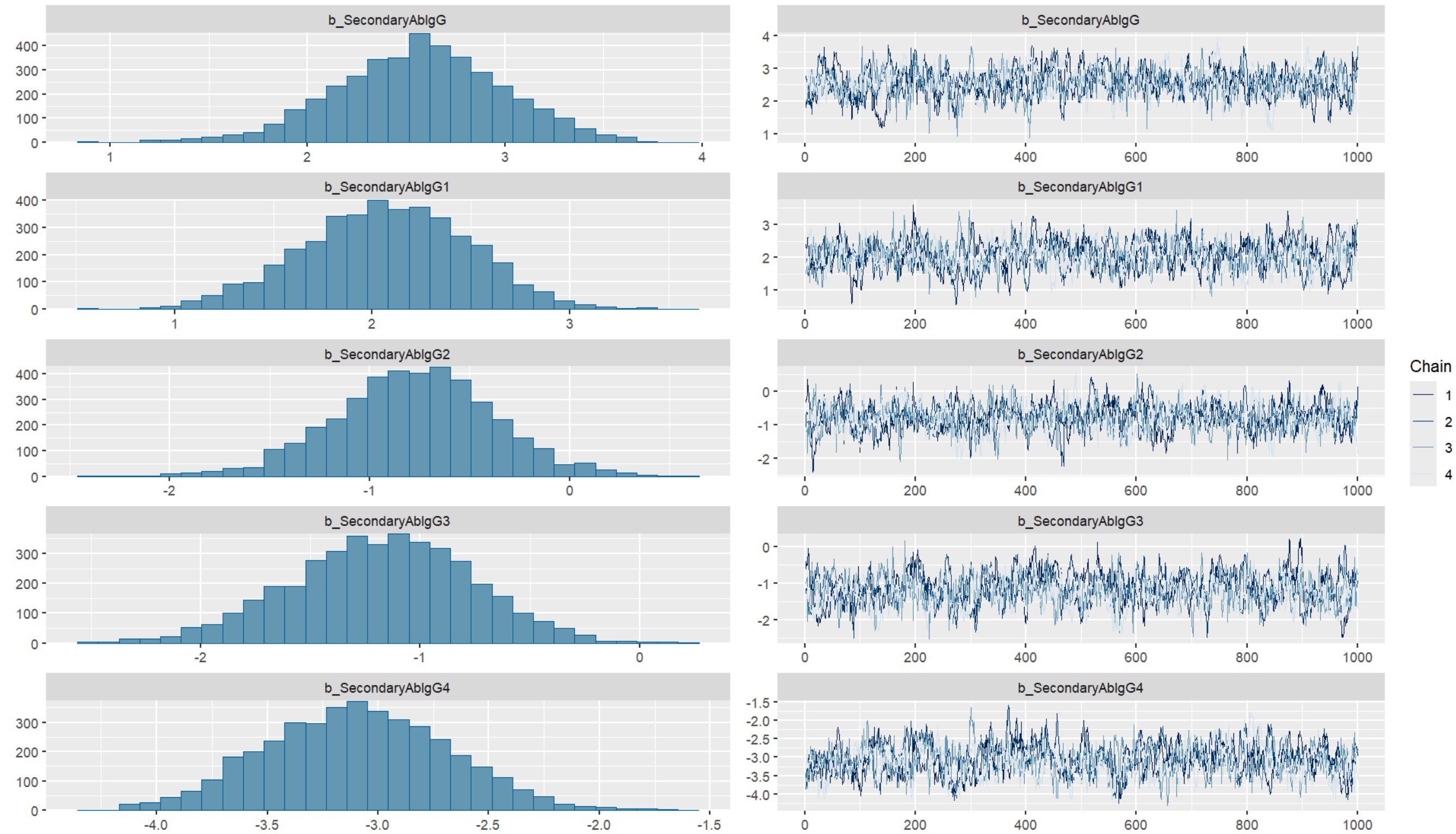

Supplementary Figure 19. Bayesian model convergence diagnostics for fixed-effect parameters. Posterior distributions (left panels) and corresponding Markov chain Monte Carlo (MCMC) trace plots (right panels) are shown for representative fixed-effect parameters. (A) Baseline and time-spline effects (intercept and DPSO segments). (B) Antigen and antibody response type effects. (C) Infection group and antibody isotype/subclass effects. Quantitative convergence diagnostics ($\hat{R}$ and effective sample sizes) are reported in Supplementary Tables 3–5.
