# Supplementary material for: Longitudinal antibody profiling after dengue reveals distinct dynamics by antibody specificity over 18 months
Source: Nat Commun. 2026 Jun 21;17:7808. doi: 10.1038/s41467-026-74406-0 (PMC13438135; doi:10.1038/s41467-026-74406-0)
Supplement: Supplementary file 2 [file 41467_2026_74406_MOESM2_ESM.pdf]

nature portfolio

Corresponding author(s): Eva Harris

Last updated by author(s): 2026/01/15

Reporting Summary

Nature Portfolio wishes to improve the reproducibility of the work that we publish. This form provides structure for consistency and transparency in reporting. For further information on Nature Portfolio policies, see our Editorial Policies and the Editorial Policy Checklist.

Statistics

For all statistical analyses, confirm that the following items are present in the figure legend, table legend, main text, or Methods section.

n/a	Confirmed	
☐	☒	The exact sample size (*n*) for each experimental group/condition, given as a discrete number and unit of measurement
☐	☒	A statement on whether measurements were taken from distinct samples or whether the same sample was measured repeatedly
☐	☒	The statistical test(s) used AND whether they are one- or two-sided<br>*Only common tests should be described solely by name; describe more complex techniques in the Methods section.*
☐	☒	A description of all covariates tested
☐	☒	A description of any assumptions or corrections, such as tests of normality and adjustment for multiple comparisons
☐	☒	A full description of the statistical parameters including central tendency (e.g. means) or other basic estimates (e.g. regression coefficient) AND variation (e.g. standard deviation) or associated estimates of uncertainty (e.g. confidence intervals)
☐	☒	For null hypothesis testing, the test statistic (e.g. *F*, *t*, *r*) with confidence intervals, effect sizes, degrees of freedom and *P* value noted<br>*Give P values as exact values whenever suitable.*
☐	☒	For Bayesian analysis, information on the choice of priors and Markov chain Monte Carlo settings
☐	☒	For hierarchical and complex designs, identification of the appropriate level for tests and full reporting of outcomes
☐	☒	Estimates of effect sizes (e.g. Cohen's *d*, Pearson's *r*), indicating how they were calculated

Our web collection on statistics for biologists contains articles on many of the points above.

Software and code

Policy information about availability of computer code

Data collection	Fluorescence data from multiplex bead–based immunoassays were acquired using iQue Forecyt® software (Sartorius iQue3 platform). The software was used to export raw bead-level fluorescence data for downstream analysis.
Data analysis	All statistical analyses were conducted in R (version 4.3.1). Data processing and visualization were performed using the tidyverse package suite, including dplyr, tidyr, and ggplot2. Frequentist linear mixed-effects models with case bootstrap resampling were fitted using lme4 and lmeresampler. Bayesian hierarchical linear mixed models were implemented using brms with a Stan backend (rstan). Non-parametric Wilcoxon tests, including Bonferroni correction and effect-size estimation, were performed using rstatix. Additional analyses used ggeffects, lspline, emmeans, and foreach. All analyses were implemented using standard functions and workflows in these packages.

For manuscripts utilizing custom algorithms or software that are central to the research but not yet described in published literature, software must be made available to editors and reviewers. We strongly encourage code deposition in a community repository (e.g. GitHub). See the Nature Portfolio guidelines for submitting code & software for further information.

Data

Policy information about availability of data

All manuscripts must include a data availability statement. This statement should provide the following information, where applicable:

- Accession codes, unique identifiers, or web links for publicly available datasets
- A description of any restrictions on data availability
- For clinical datasets or third party data, please ensure that the statement adheres to our policy

All data associated with this study are present in the main text or Supplementary Information. Simulated datasets for the statistical analyses are available via Zenodo (https://doi.org/10.5281/zenodo.19155076). After obtaining approval from the UC Berkeley Committee for the Protection of Human Subjects, individual data, which are subject to human-subjects protections, can be shared with external researchers for figure reproduction. For access arrangements, please contact E.H. at eharris@berkeley.edu. Standard data and material transfer agreements govern all data used in this study. Agregated source data are provided with this paper.

Research involving human participants, their data, or biological material

Policy information about studies with human participants or human data. See also policy information about sex, gender (identity/presentation), and sexual orientation and race, ethnicity and racism.

Reporting on sex and gender	Information about sex was collected based on self-reporting.
Reporting on race, ethnicity, or other socially relevant groupings	N/A
Population characteristics	Participant characteristics and covariate relevant to the study (age, sex distribution, infecting serotype, disease severity) are summarised in Supplementary Table 1.
Recruitment	Participants were recruited from an ongoing pediatric dengue hospital-based study at the Hospital Infantil Manuel de Jesús Rivera in Managua, Nicaragua. Children aged 6 months–14 years presenting to the hospital within 1–7 days of symptom onset and meeting the clinical case definition for dengue from August 24, 2010, to November 26, 2013, were invited to participate. Enrollment was based on prospective presentation to care and was independent of disease severity, sex, or serotype. Written informed consent was obtained from a parent or guardian, and assent was obtained from children aged 6 and older. Participants with laboratory-confirmed dengue virus (DENV) infection and known infecting serotype (DENV1 or DENV3) who consented to longitudinal follow-up were eligible for inclusion in this study. Convalescent and follow-up plasma samples were collected at approximately 14–28 days, 3 months, 6 months, and 18 months post-symptom onset. Potential bias : Participants were enrolled prospectively at presentation to a single hospital (HIMJR, Managua) within 7 days of symptom onset, which may introduce a severity bias toward more clinically apparent cases — milder dengue infections that did not prompt hospital evaluation are not represented. Longitudinal follow-up requires sustained participation, which may bias the analytical cohort toward more compliant or geographically stable families. Catchment is urban and peri-urban Managua, and findings may not generalise to rural or non-endemic settings. These biases are expected to shift the cohort toward more clinically apparent and more compliant participants but are unlikely to differentially affect post-primary versus post-secondary comparisons, which are matched within the same cohort.
Ethics oversight	The Pediatric Dengue Hospital-based Study was reviewed and approved by the institutional review boards of the University of California, Berkeley (2010-06-1649) and the Nicaraguan Ministry of Health (NIC-MINSA/CNDR CIRE-01/10/06-13). Parents or legal guardians of all subjects provide written informed consent, and subjects ≥6 years old provide assent.

Note that full information on the approval of the study protocol must also be provided in the manuscript.

Field-specific reporting

Please select the one below that is the best fit for your research. If you are not sure, read the appropriate sections before making your selection.

☒ Life sciences ☐ Behavioural & social sciences ☐ Ecological, evolutionary & environmental sciences

For a reference copy of the document with all sections, see nature.com/documents/nr-reporting-summary-flat.pdf

Life sciences study design

All studies must disclose on these points even when the disclosure is negative.

Sample size	Sample sizes were determined by the availability of well-characterized longitudinal samples in the PDHS that met all inclusion criteria (laboratory-confirmed dengue, known infecting serotype, classification as primary or secondary infection, and availability of plasma at early convalescent, 3-month, 6-month, and 18-month timepoints). No formal power calculation was performed. Primary DENV1 and DENV3 groups were fixed at 20 participants each to allow matched comparisons across serotypes, while secondary DENV1 and DENV3 groups were defined by sample availability. The sufficiency of this sample size is supported empirically by the observed effect sizes: across 118 paired Wilcoxon signed-rank tests, 60 (51%) reached statistical significance after Bonferroni correction for multiple comparisons. Among these significant

comparisons, the median effect size (Wilcoxon r) was 0.61, classified as "large" by the conventional Cohen thresholds ($r \geq 0.5$), and the interquartile range was 0.49–0.69. No comparisons with small effect sizes ($r < 0.3$) survived correction, indicating that the design had adequate power to detect biologically meaningful differences. The longitudinal repeated-measures structure (four timepoints per participant) further increases statistical efficiency by leveraging within-participant correlation through the linear mixed-effects framework.

Data exclusions

No samples were excluded from the analyses after applying the predefined inclusion criteria.

Replication

Median fluorescence intensity of each isotype, at different timepoint, was acquired with an iQue3 instrument (Sartorius). Assay performance was first validated using a pilot set of representative plasma samples from the study to confirm the robustness and reproducibility of the multiplex bead–based measurements across antibody isotypes and subclasses. Pilot and study runs replication was successful, and inter-plate comparisons of reference samples confirmed reproducibility across experimental runs. All plasma samples were subsequently measured using multiplex bead–based immunoassays. Each sample was tested in four consecutive four-fold serial dilutions, with one technical replicate per antibody isotype or subclass. Each assay plate included negative controls and a set of reference (“batch-effect”) samples that were run across plates to ensure comparability between plates and experimental runs.

Randomization

Participants were assigned to infection groups based on biological status (primary vs secondary infection and infecting serotype). Primary DENV1 and DENV3 cases were randomly selected from eligible participants to generate balanced and matched groups. For laboratory measurements, plasma samples were randomized across assay plates by infection group to minimize batch effects.

Blinding

Blinding was not possible because the same investigator selected the samples and performed the multiplex assays. However, samples were randomly distributed across plates with equal numbers of each infection group per plate to avoid batch effects.

Reporting for specific materials, systems and methods

We require information from authors about some types of materials, experimental systems and methods used in many studies. Here, indicate whether each material, system or method listed is relevant to your study. If you are not sure if a list item applies to your research, read the appropriate section before selecting a response.

Materials & experimental systems

n/a	Involved in the study
☐	☒ Antibodies
☒	☐ Eukaryotic cell lines
☒	☐ Palaeontology and archaeology
☒	☐ Animals and other organisms
☒	☐ Clinical data
☒	☐ Dual use research of concern
☒	☐ Plants

Methods

n/a	Involved in the study
☒	☐ ChIP-seq
☒	☐ Flow cytometry
☒	☐ MRI-based neuroimaging

Antibodies

Antibodies used

Antibody subclass/isotype binding was detected using PE-coupled mouse anti-human detection antibodies against IgA (SouthernBiotech #2050-09), IgM (SouthernBiotech #9020-09), total IgG (SouthernBiotech #9040-09), IgG1 (Southern-Biotech #9052-09), IgG2 (SouthernBiotech #9060-09), IgG3 (Southern-Biotech #9210-09), and IgG4 (SouthernBiotech #9200-09). All antibody were used at 1:1500.

Validation

All the antibodies used were validated for the species and application by the manufacturer and in previous studies (DOI: 10.1126/scitranslmed.abm3151; doi: 10.1016/S1473-3099(24)00566-8)

Plants

Seed stocks

Report on the source of all seed stocks or other plant material used. If applicable, state the seed stock centre and catalogue number. If plant specimens were collected from the field, describe the collection location, date and sampling procedures.

Novel plant genotypes

Describe the methods by which all novel plant genotypes were produced. This includes those generated by transgenic approaches, gene editing, chemical/radiation-based mutagenesis and hybridization. For transgenic lines, describe the transformation method, the number of independent lines analyzed and the generation upon which experiments were performed. For gene-edited lines, describe the editor used, the endogenous sequence targeted for editing, the targeting guide RNA sequence (if applicable) and how the editor was applied.

Authentication

Describe any authentication procedures for each seed stock used or novel genotype generated. Describe any experiments used to assess the effect of a mutation and, where applicable, how potential secondary effects (e.g. second site T-DNA insertions, mosiacism, off-target gene editing) were examined.
