# Supplementary material for: Longitudinal antibody profiling after dengue reveals distinct dynamics by antibody specificity over 18 months
Source: Nat Commun. 2026 Jun 21;17:7808. doi: 10.1038/s41467-026-74406-0 (PMC13438135; doi:10.1038/s41467-026-74406-0)
Supplement: Supplementary file 3 [file 41467_2026_74406_MOESM3_ESM.pdf]

nature portfolio

Peer Review File

Longitudinal antibody profiling after dengue reveals distinct dynamics by antibody specificity over 18 months

Corresponding Author: Dr Eva Harris

This file contains all reviewer reports in order by version, followed by all author rebuttals in order by version.

Version 0:

Reviewer comments:

Reviewer #1

(Remarks to the Author)
This is a very interesting study that characterises how different dengue antibody subsets (by antigen, isotype, subclass, and various cross-reactivities) rise, wane, or persist over 18 months following primary or secondary dengue infections. The authors show that dengue antibody responses differ by antigen, isotype, and infection history. In contrast to other literature, the study highlights the rising prevalence of cross-reactive antibodies following primary infections and the unexpected persistence of IgA and IgM. These findings have significant implications for our understanding of dengue immunity, mechanistic correlates of protection, and the risks of severe disease; however I have some clarifications/concerns about the choice of methods.

1. It would be helpful to understand why antibody titres were modelled using linear mixed effects, which describes trends in the data, rather than simpler but more mechanistic approaches like a biphasic model (e.g., https://doi.org/10.1093/infdis/jiu219) , which would provide greater insights into the dynamics of different antibody subsets.
2. While the authors provide a rationale for their chosen knots at 100 and 200 days, it would be helpful to understand how sensitive their findings are to the number and positioning of the knots, especially given that they assume linear antibody kinetics within these time periods.
3. It would be helpful to disentangle further the role of boosting in some of the long-term kinetics when stable or rising titres are observed, especially given the authors' suggestion that rising cross-reactive IgG titres could be linked to ADE.
4. It's not clear to me from the methods how the authors tested for significant differences in titre levels between different comparator groups at each time point, and whether any corrections were made to account for the multiple comparisons (84 antibody subsets, primary and secondary infections, at multiple time points).
5. It would be helpful to include some discussion of any potential cohort effects—for instance, the timing or serotypes of prior exposures.
6. As far as I can tell, the homogeneous and cross-reactive compartments measured in the study will both capture cross-reactive antibodies, so I am not sure how relevant these comparisons are. I am also not sure about the use of DENV4 and especially DENV2 as proxies for cross-reactive antibodies. While DENV4 circulation is lower than the other serotypes, previous work by the authors has identified its asymptomatic circulation in Nicaragua.
7. Did all participants contribute to all sample time points? Why were samples collected during the acute phase not included to capture early changes in immune dynamics?
8. For traditional neutralisation assays, the reference virus will determine the magnitude of serotype-specific responses. Is this true of the proteins used for multiplex assays? If so, then this could cloud the homotypic versus cross-reactive inferences being made. Either way, it would be helpful to comment on this in the discussion.
9. By design, this study included only clinical dengue, limiting inference that can be made about the relationship between antibody kinetics and clinical risk or protection. This should at least be commented on.

(Remarks on code availability)
I was not able to find the code using the link provided.

Reviewer #2

(Remarks to the Author)

Bos et al studied antibody responses to DENV and ZIKV E, EDIII, and NS1 antigens in serial blood samples collected over 18 months following symptomatic dengue in 79 children using a multiplexed bead-based immunoassay (105 measurements per blood sample). Antibody responses were compared over time, between the infecting virus and heterologous viruses (DENV and ZIKV), and by antigen, isotype, and IgG subclass based on linear mixed-effects modeling. The main findings were a) some DENV serotype-cross-reactive antibody responses increased rather than decreased from 6 to 18 months post-infection, b) responses to different viruses and antigens did not all follow the same trends over time, c) a significant fraction of subjects were positive for IgA, IgM, and IgG3 at 18 months post-infection. The authors conclude that these data offer novel insights into flavivirus immunity and disease risk and have implications for vaccine design and serodiagnosis.

The large multiparameter dataset, longitudinal nature of the data, and well-characterized cohort of acute dengue cases all constitute substantial strengths of the work. There are, however, significant issues regarding the statistical methods and interpretation of differences. While the questions addressed in this analysis are of significant import, and the results of interest, the conclusions as currently stated are not adequately supported.

Major comments:

1. Throughout the manuscript, the authors present mean values derived from statistical modeling. They calculated slopes (decay rates) for each interval from the derived mean values and transformed the slopes into estimated half-lives. Confidence intervals for mean values and slopes were estimated from 1000 bootstrap replicates of each model. Statistical inferences were mostly drawn from the comparisons of the calculated slopes although they are presented with the calculated half-life values. According to the figure legends, differences were considered significant at the $p<0.05$ level. The manuscript does not clearly state how p values were determined. In any event, it is not clear that this analytic approach is useful or justified, particularly since modeling was done separately for each response considered and the model allowed independent slopes for each interval between actual data points. A direct comparison of actual slopes for each subject would have more accurately accounted for the variances in the data.

2. Multiple statistical comparisons were performed, e.g., "separately for each antigen, viral response category, and antibody isotype and subclass" (lines 544-545), corresponding to at least 63 (3x3x7) different comparisons for each interval considered (C-3M, 3-6M, and 6-18M). The potential for type I error needs to be more carefully considered in the Discussion.

3. The general form of the statistical model provided (lines 548-550) incorporates a single primary factor A along with days post symptom onset, age, and sex as predictor variables. In this model, treatment of a binary variable, e.g., primary versus secondary infection, is straightforward. It is not clear how this formula was applied for comparisons across factors that were not binary, e.g., viral antigen or viral response, where 3 (or 4) values were possible, as is presented in several of the figures.

4. The authors should include the serotype of the current infection (DENV1 versus DENV3) as a potential predictor variable in their analysis, given published evidence of significant differences in immune responses. In the Discussion, the authors should comment on any differences according to the serotype of infection.

5. The study design indicates (line 498) that blood samples were also collected at 12 months after symptom onset. Where these samples tested? Inclusion of these data would strengthen the statistical modeling between months 6 and 18.

6. In much of the manuscript, the authors equate MFI values to antibody levels, and several of the novel insights offered are based on this assumption. The authors acknowledge (e.g., lines 167-168) that MFI values may be affected by interactions between antibody subsets. However, this and other factors such as antibody affinity are not adequately considered as alternative explanations for observed response kinetics, e.g., a "rise" in IgG4 (line 433).

7. It is evident from individual response data (Figures S2, S4, and S9) that a subpopulation of subjects showed large increases in some antibody responses between months 6 and 18. While the authors acknowledge (line 336) the possibility of re-exposure to DENV in their study population, this is not adequately considered as an alternative explanation for several of their novel conclusions.

8. For several of the responses, MFI values below background are included in the statistical modeling. Conclusions drawn from these values are problematic.

9. The authors highlight "long-lasting antiviral IgA, IgM, and IgG3 antibodies" and suggest that these findings have implications for the serological diagnosis of dengue. In their analysis, seropositivity at 18 M was defined as responses exceeding the mean + 3 SD MFI of five DENV-naïve samples included on each plate. In at least several cases, these positive responses are very low in magnitude. More extensive testing of the assay with a larger number of control samples would be needed to establish the practicality of this cutoff for clinical diagnosis. This limitation should be considered in the Discussion.

Minor comments:

10. Primary versus secondary infections were defined based on the serological response to infection, in keeping with normal practice. However, this should be considered in interpreting the differences in antibody kinetics, particularly for the first interval (C-3M).

11. Figures showing the half-life of antibody responses (e.g., Figure 1B) are confusing and difficult to interpret since the y

axis scale is uninformative and crosses -inf. It appears that the graphs are actually decay rates with values for y axis labels transformed to half-lives. Labeling these as decay rates instead would more accurately depict the information.

12. The investigators tested sera at 4 dilutions, but present the analysis of data form the 1:800 dilution only. For their most novel findings, did other serum dilutions show similar results?

13. Line 207- Figure 4E is incorrect cited in the text. The figure for the interval 6-18M does not show a statistically significant difference.

14. Figure 7- y axis values presented in panel (a) do not appear to match corresponding results in Figure 3. Panel (b) is incorrectly described in the legend as resulting from modeling; this figure shows individual subject values. For panel (c) none of the subjects is classified as "stable" (noted in the legend).

15. Line 574- "half-life of zero" is incorrect.

(Remarks on code availability)

Reviewer #3

(Remarks to the Author)
What are the noteworthy results?
- The authors describe the kinetics and breadth of different antibody responses to different dengue virus proteins and domains within proteins following primary and secondary D1 and D3 infections. There is specific attention to defining the rise and fall of homotypic and cross reactive antibody profiles. Rise in cross reactive IgG antibodies over time (not waning) was a unique finding as was the persistence of IgM and IgA antibody populations.

Will the work be of significance to the field and related fields? How does it compare to the established literature? If the work is not original, please provide relevant references.
- The work is relevant to the dengue immunology field and describes specific immunologic profiles over time which few other groups have described. The concept of longitudinal profiling is not new but the measured endpoints and breadth of the same are unique.

Does the work support the conclusions and claims, or is additional evidence needed?
- The authors lay the foundation of the significance of their work on the theory of ADE and it being the reason why people develop severe disease. ADE is a theory and should not be taken as a proven or definitive immunopathologic mechanism for severe dengue. Building off of the ADE theory as fact and using this as a bridge between the authors findings and speculations of mechanisms of severe disease weakens the conclusions. It has been a significant challenge for the field to relate in time and space a baseline immunologic profile, dengue virus infection, and subsequent virologic and immunologic endpoint which are significantly associated with particular clinical outcomes as described in the ADE theory. The manuscript would be strengthen with less speculation and more attention paid to historic data using different methods (neut ab) and how their data differs (rise and fall of cross reactive abs in particular).

Are there any flaws in the data analysis, interpretation and conclusions? Do these prohibit publication or require revision?
- The findings are interesting and hypothesis generating but less informative as it relates to immunopathologic mechanisms or risk of severe disease outcomes. A revision with an expanded focus on historic data (they do discuss some) and the differences between those data and the authors data would be welcome.

Is the methodology sound? Does the work meet the expected standards in your field?
- Methodology is sound and meets standards.

Is there enough detail provided in the methods for the work to be reproduced?
- Many of the assays have been described before but there was at least one reference which did not seem to track with the text (REF 46). Multiplex assay is described well. Stats review is required.

Abstract:
“can enhance disease”
- More accurate to say “have been associated with…”
Introduction:
“lack of therapies”
- Evidenced based and highly effective therapeutic algorithms have been available for decades. Perhaps “DENV-specific” or “anti-viral” therapies is more accurate.
“Understanding immune responses is critical”
- Perhaps an overstatement. Safe and effective vaccines have been licensed and deployed into use without detailed understanding of immunogenicity and its association with clinical outcomes. Authors should make the case for why dengue is different (immunopathology of disease for example).
“which can exacerbate infection”
- Unclear what this means? Are the authors making a virologic, immunologic, or clinical observation? The references

provided use in vitro and small animal studies to explain a complex immunopathologic processes which occurs in humans and result in various clinical phenotypes. Qualifying the statement to be less conclusive seems appropriate.
“stable long-term antibody levels”
- What type of antibodies? The remainder of that paragraph requires clarification as to what type of antibodies the authors are referring to.
Results:
“post-primary”
- How was primary and secondary defined?
“Since DENV E-binding XR antibodies play”
- Are believed to play or are associated with may be more accurate phrasing.
“enhancement or protection”
- Enhancement or protection of what? Virologic, immunologic, clinical outcomes? Recommend clarifying what is specifically being described.
“outside the DENV1-4 serocomplex (ZIKV-XR”
- What was the dengue immune profile of the individuals who contributed ZIKV XR samples? Were they dengue naïve? If not, was the infecting DENV serotype(s) known? Could the timing of DENV infection in relation to ZIKV infection in these folks impact assay measurements?
“seropositivity impacts protection on the population level”
- I think this requires explanation. We have seen seropositivity NOT associated with protection in several vaccine trials. A short description and references would strengthen the manuscript.
“variability in antibody responses shape protection”
- Again, this concept requires increased clarity. What is population level protection? Are the authors proposing herd immunity to a specific DENV type or types due to high levels of type specific and cross reactive immunity in a population? A short description and references would strengthen the manuscript.
Discussion
“assumption that cross-reactive antibody levels”
- Is it more accurate to state ALL cross reactive… There is plenty of antibody data measured using other assay platforms (classic PRNT or HAI) which clearly demonstrate waning over time after infection.
“documented following 1° ZIKV infection”
- In dengue immune individuals? If so, I would state the same.
“or in vaccinees”
- Differences would be expected I would imagine considering the simultaneous exposure to 4 DENV types.
“facilitate DENV ADE”
- Again, the authors should be more precise in the language they use (ADE, enhancement, etc.). The accessibility of the manuscript to the non-dengue expert clinician or scientist will improve if terminology and descriptions of concepts are clearer.
Methods:
“Blood samples are collected at the time of hospital presentation “
- The study methods focus on collection of samples and characterization of immune responses from people who are infected, ill, and ill enough to present to the hospital for care. This would seem to eliminate the largest proportion of both primary and secondary infections (asymptomatic or subclinical) making the generalization of these data highly problematic and calls into question the mechanistic relevance. Please explain why you disagree.
“longitudinal follow-up of convalescent samples at 3, 6, 12, 18…”
- How would flavivirus exposures which were subclinical be captured during the prolonged convalescent period? Would undetected homotypic or heterotypic or non-dengue flavi exposure impact outputs and if so how?
- The premise of the manuscript is that these antibody profiles provide new insight into mechanisms of severe disease, but cohort and vaccine studies demonstrate the period of risk for severe disease is after 18 months have passed from a first infection. In this study the last collection is at 18 months. Do the authors think additional timepoints would yield more informative results as it relates to understanding what happens with clinical outcomes following second, sequential, heterotypic infections?
“DENV infection is classified by an iELISA titer below 20”
- How primary and secondary infections are defined is extremely important. Is the ref 46 the correct reference to define how these cutoffs were derived?
- Are the authors using secondary infections to mean 2nd sequential infection with different dengue virus type or simply 2 or more infections? If the former, how is it confirmed a second infection was captured?
Supp Table 1
- How was DHF defined? WHO 1997 criteria?
Supple Figure 1
- Nice schematic, may belong in main manuscript.

(Remarks on code availability)

Version 1:

Reviewer comments:

Reviewer #1

(Remarks to the Author)
I am satisfied that the authors have addressed all my comments. The paper's statistical methods have been strengthened, especially regarding the choice of models and the calculation of significance. However, the code provided is still insufficient to reproduce the main findings.

(Remarks on code availability)
The authors have provided the main R file containing the methods (Models.R). However, I was unable to run the code as the main data input was not provided. If the authors cannot provide the data, they should provide simulated data instead. Additionally, there is insufficient documentation to reproduce the model code, including the absence of a README file and limited guidance within the code.

Reviewer #2

(Remarks to the Author)
In this revised manuscript, the authors have substantially clarified the basis for their inferences and have supplemented their initial analysis based on linear mixed models with alternative analyses including a more direct non-parametric comparison. The authors have also made substantial efforts to address criticisms and questions regarding the framing of their conclusions.

As noted in the previous review, this dataset has high overall significance. Therefore, the comments below focus on remaining issues in the presentation and interpretation of the data.

Major comments:

1. The authors clarify that they define "significant" differences when the 95% CI of the comparison group did not overlap with the corresponding point estimate for the reference group (or zero in the case of defining rising or waning). While they removed textual references to "statistically significant" and no longer cite p values, the use of asterisks in figures implies statistical significance that is not supported by this analysis. This will be misleading to most readers and should be deleted.

2. As pointed out in the prior review, primary versus secondary infections were defined based on the antibody response to infection, specifically, the antibody response measured at the convalescent time point. Whether iELISA or multiplex assay is used to make this determination is irrelevant to the potential implications, particularly for analyses of antibody trajectories between early convalescence and 3 months. The potential misclassification of cases is an important limitation of their analysis that needs to be acknowledged in the Discussion.

3. In numerous places in the manuscript, the authors draw conclusions regarding the timing of peak responses during the C-3M window (e.g., "delayed peak", "delayed initiation"). Since antibody responses were only measured at the convalescent and 3M time points, peak responses likely were not captured. These statements should be revised accordingly.

4. The authors provide a formula for the linear effects model using factors with 3 or more levels (lines 565-571). However, it is not clear that these models were actually used in the manuscript, as it appears that all of the curves presented in the manuscript were based on the linear models of primary versus secondary. If so, the Methods should be revised accordingly. If this is not the case, the authors should make clear which model was used to generate data presented in each figure.

5. In the prior review, it was suggested that the authors comment on whether data from the other serum dilutions supported their most novel findings. The authors' response justifies use of the 1:800 dilution for the quantitative analyses presented in the manuscript. However, it would be expected that other dilutions would fall within the interpretable range for specific comparisons of interest that they consider to provide "novel conceptual insights". The reporting of such supportive information (or lack thereof) would be very helpful to the scientific community given that the data already exist.

6. Abstract- Statements of conclusions in the Abstract should be as accurate as possible. The following changes are recommended- "IgG antibodies against the envelope protein rise" (line 27) to "IgG antibody responses against the envelope protein rise" and "substantial seropositivity of IgA, IgM, and IgG3 at 18 months post-infection was observed" (line 29) to "a substantial fraction of subjects still had IgA, IgM, and IgG3 responses above the assay background at 18 months post-infection".

Minor comments:

7. Figure 1-5- "remained at or below infinite" in the legends should read "remained at or below zero".

8. Figures 4b/c, 5b/c, 7b- y axis values (all <0.05) do not appear to comport with the definition of fold change provided on lines 655-656. (Perhaps the authors divided by the number of days between samples?)

9. Figures S10, S11- The distribution of values on both x and y axes is inconsistent with the data presented in other figures. Correlation (r) values would be more informative than p values. Shared areas should be defined. The legends should be revised- maximum values were not WITHIN 3 months of infection according to the information in Table S1.

10. Line 133- Incorrect reference is made to a non-existent Figure 1F.

11. Lines 136-140- Reference to "levels" in this paragraph are particularly problematic; "responses" would be a more accurate description of the findings.

12. Lines 251-252- The statement "higher population seropositivity is associated with longer interepidemic periods and lower force of infection" is not an accurate description of the paper cited nor is it particularly relevant to the analysis described in this section.

13. Lines 286-287- The authors should clarify that there are referring to the interval of 6-18 M.

14. Lines 292-293- "demonstrated this relationship more" should be revised to clarify whether the authors are referring to the slope or correlation coefficient.

15. Line 342- In-text citations are incorrectly formatted; "change in the antigenic composition over time" is confusing and should be revised for clarity.

16. Line 527- The following sentence needs further clarification- "Background-subtracted MFI values were calculated using average of naïve individuals + 3SD". The sentence should be clarified to state that "Background MFI values were calculated using average of naïve individuals + 3SD". Also, the authors should clarify whether these background values were log2 transformed before substraction (this is implied by line 526). It would be helpful to provide the range of background values, recognizing that there may be substantial variation between subsets.

17. Lines 588-589- "classified as significantly waning or rising when its 95% confidence interval excluded zero (i.e., infinite)" should be revised to "classified as significantly waning or rising when the 95% confidence interval of its decay rate excluded zero (i.e., infinite half-life)".

(Remarks on code availability)

Reviewer #3

(Remarks to the Author)
The authors provide an extensive rebuttal to the reviewers comments and have made significant changes to the manuscript. Statistical methodology and computational questions aside, the reviewer believes the authors have adequately addressed the comments and critiques.

The modifications in interpretation and representation of results have strengthened the paper and its accessibility to the non-dengue expert. The data are presented as novel but with a properly measured assessment of their generalizability and their limitations as it relates to understanding dengue pathogenesis.

The reviewer appreciates the long list of papers supporting the associations between first and second infections and immunologic profiles and dengue infection clinical outcomes. The reviewer's point is that association is not causation. Association can be a correlate if it meets statistical thresholds but even a correlate is not causation (unless a mechanistic correlate). The point was the importance of nuance in these discussions and not leaving the impression ADE is THE ONLY proposal. The modified language is very good and will appeal to a more general audience.

(Remarks on code availability)

Open Access This Peer Review File is licensed under a Creative Commons Attribution 4.0 International License, which permits use, sharing, adaptation, distribution and reproduction in any medium or format, as long as you give appropriate credit to the original author(s) and the source, provide a link to the Creative Commons license, and indicate if changes were made.
In cases where reviewers are anonymous, credit should be given to 'Anonymous Referee' and the source.
The images or other third party material in this Peer Review File are included in the article's Creative Commons license, unless indicated otherwise in a credit line to the material. If material is not included in the article's Creative Commons license and your intended use is not permitted by statutory regulation or exceeds the permitted use, you will need to obtain permission directly from the copyright holder.
To view a copy of this license, visit https://creativecommons.org/licenses/by/4.0/

REVIEWER COMMENTS

Reviewer #1 (Remarks to the Author):

This is a very interesting study that characterizes how different dengue antibody subsets (by antigen, isotype, subclass, and various cross-reactivities) rise, wane, or persist over 18 months following primary or secondary dengue infections. The authors show that dengue antibody responses differ by antigen, isotype, and infection history. In contrast to other literature, the study highlights the rising prevalence of cross-reactive antibodies following primary infections and the unexpected persistence of IgA and IgM. These findings have significant implications for our understanding of dengue immunity, mechanistic correlates of protection, and the risks of severe disease; however, I have some clarifications/concerns about the choice of methods.

1. **It would be helpful to understand why antibody titres were modelled using linear mixed effects, which describes trends in the data, rather than simpler but more mechanistic approaches like a biphasic model (e.g., https://doi.org/10.1093/infdis/jiu219), which would provide greater insights into the dynamics of different antibody subsets.**

A: We appreciate the suggestion and agree that biphasic (two-exponential) models can be informative when parameters are well-identified. In our study, we analyzed 84 antibody subsets (isotypes/subclasses × antigens × immune response categories) with a fixed sampling schedule (early convalescent, 3, 6, and 18 months). To balance biological interpretability and comparability across dozens of subsets, we modeled log-titers using segmented generalized linear mixed-effects models (GLMM) with *a priori* breakpoints at 100 and 200 days (0–100 d, 100–200 d, >200 d) with $\log_2$ titers, thus modeling an exponential decay model with three phases. Critically, the GLMM scales to our 84 antibody subsets, whereas 84 mechanistic models would require target-specific starts, tight constraints, and convergence checks/refits, which adds computational and interpretive burden. Our approach accommodates participant-level heterogeneity and permits covariate adjustment (age and sex) and explicit handling of antibody boosts that violate a single global two-exponential decay.

Importantly, our segmented linear model already approximates a biphasic process: within each phase, the dominant exponential renders log-titer approximately linear in time, while fixed breakpoints at 100/200 days anchor the early/late regimes uniformly across subsets, making comparability easier.

2. **While the authors provide a rationale for their chosen knots at 100 and 200 days, it would be helpful to understand how sensitive their findings are to the number and positioning of the knots, especially given that they assume linear antibody kinetics within these time periods.**

A: We acknowledge the importance of testing the assumptions of the segmented linear model. Our proposed model uses two *a priori* knots at 100 and 200 days, describing a rapid early decline followed by slower decay phases. To evaluate robustness, we systematically compared this full model (two knots/three segments) with three simpler alternatives: a single-knot model at 100 days (partial model 1), a single-knot model at 200 days (partial model 2), and a no-knot linear model (zero model). All models were fit using identical covariate structure (age, sex), primary versus secondary immune response (IR) × time interactions, and individual-level random intercepts, enabling direct comparison of fit while holding all other assumptions constant.

We tested the interaction of IR and time segments (on full and alternative models) adjusting by age and sex and with random intercepts for each individual. Across all 84 antibody subsets, the full model provided the lowest AIC (i.e., best fit) for 46.4% of subsets, followed closely by the 100-day single-knot model (38.1%). The AIC differences between the full model and the best-performing simpler model were small (mean $\Delta AIC = -2.39$; median = –0.933), indicating that the two-knot structure provides a modest but consistent improvement in fit across subsets (Tables R1-6). These findings support our decision to adopt a unified two-knot model for all subsets, as this structure captures the expected phases while maintaining comparability across the 84 antibody

categories. For the following tables "N" reflects the number of models out of 84 total models where that approach demonstrated the lowest AIC value.

Table R1: Model comparisons of antibody dynamics by primary and secondary immune response.

Best model	N	Percent when this model fits best (%)
Full model	39	46.4
Partial model 1	32	38.1
Partial model 2	7	8.33
Zero model	6	7.14

Table R2: AIC delta comparisons between full and partial 1 model by primary and secondary immune response.

Subsets	Mean Delta	Median Delta	SD Delta	Percent when model full is better (%)	Percent when model partial 1 is better (%)
84	-2.39	-0.933	6.31	51.2	48.8

Table R3: Model comparisons of antibody dynamics by antigen.

Best model	N	Percent when this model fits best (%)
Full model	22	39.3
Partial model 1	17	30.4
Partial model 2	9	16.1
Zero model	8	14.3

Table R4: AIC delta comparisons between full and partial 1 models by antigen.

Subsets	Mean Delta	Median Delta	SD Delta	Percent when model full is better (%)	Percent when model partial 1 is better (%)
56	-2.88	-1.03	9.49	57.1	42.9

Table R5: Model comparisons of antibody dynamics by antibody response (infecting virus vs cross-reactive).

Best model	N	Percent when this model fits best (%)
Full model	16	38.1
Partial model 1	15	35.7
Partial model 2	8	19.0
Zero model	3	7.14

Table R6: AIC delta comparisons between full and partial 1 model by antibody response.

Subsets	Mean Delta	Median Delta	SD Delta	Percent when model full is better (%)	Percent when model partial 1 is better (%)
42	-6.95	-3.05	15.2	52.4	47.6

3. **It would be helpful to disentangle further the role of boosting in some of the long-term kinetics when stable or rising titres are observed, especially given the authors' suggestion that rising cross-reactive IgG titres could be linked to ADE.**

A: We agree that distinguishing true long-term increases in antibody levels from subclinical boosting events is an important challenge when interpreting rising or stable antibody levels, particularly for cross-reactive IgG

responses. In our study, we lacked virological data during the long-term follow-up period to definitively attribute these increases to endogenous kinetics versus unrecognized flavivirus exposures. We discuss this further as a potential limitation in lines 457-475:

> *"Importantly, although occasional boosting from unrecognized DENV exposures may contribute to stable or rising antibody levels in some subsets, the distinct long-term waning or rising patterns observed across specific antibody subsets suggest that boosting alone is unlikely to fully account for our main findings. However, boosting due to subclinical re-exposures cannot be ruled out.78 Antibody dynamics following a single 1° exposure in travelers from non-endemic regions may follow different trajectories."*

4. **It's not clear to me from the methods how the authors tested for significant differences in titre levels between different comparator groups at each time point, and whether any corrections were made to account for the multiple comparisons (84 antibody subsets, primary and secondary infections, at multiple time points).**

A: The Reviewer makes a good point. We tested significant differences in antibody *decay rate* across groups (i.e., infection history, antigen, and antibody isotype/subclass), not titers, throughout this study. Because p-value calculations for contrasts in non-parametric case-bootstrapped linear mixed models (LMM) can be unreliable and complex to calculate (*Davison AC, Hinkley DV. 1997. Bootstrap methods and their application. Cambridge, UK: Cambridge University Press*), we instead defined significance using the non-parametric bootstrap distribution of antibody decay rates. For each antibody subset and time interval, we summarized the bootstrap distribution of the decay rate with a point estimate and 95% confidence interval. A subset was classified as significantly waning or rising when its 95% confidence interval excluded zero. Differences between groups (e.g., primary vs secondary) were assessed using a confidence-interval overlap criterion, whereby a group was considered to differ from the reference if its 95% confidence interval did not overlap the reference group's point estimate. All figure legends have been revised to reflect this approach, and references to "$p < 0.05$" have been removed throughout the manuscript.

The following statement was added to the Methods to clarify our approach (lines 587-593):

> *"Differences between groups were assessed using contrasts in decay rates obtained from a non-parametric bootstrap of individual trajectories. For each antibody subset and time interval, a subset was classified as significantly waning or rising when its 95% confidence interval excluded zero (i.e., infinite). In addition, within each stratum, differences between groups were evaluated using a confidence interval-overlap criterion, whereby a group was considered to differ from the reference group if its 95% confidence interval for the decay rate did not overlap the reference group's point estimate."*

We acknowledge the concern regarding multiple comparisons. As described above, our inference framework does not rely on asymptotic p-values but instead on non-parametric case (individual-level) bootstrap of antibody decay trajectories. This approach explicitly captures between-individual variability and uncertainty arising from the relatively small number of participants contributing to each antibody subset and time interval. Given the heterogeneity of immune responses and the limited effective sample size within strata, this case-bootstrap–based confidence interval approach is usually more conservative and robust than standard distribution-based regression inference.

To further assess robustness and limit the risk of type I error in this high-dimensional setting, we performed two complementary sensitivity analyses: (i) we fit a single unified Bayesian hierarchical linear mixed model (BLMM) with the same spline-based structure and weakly informative priors on fixed and random effects. This model jointly estimates all decay parameters within a multilevel framework, inducing partial pooling and shrinkage across antibody subsets and thereby reducing the risk of false positives due to overfitting or multiple testing. (ii) we implemented a model-independent non-parametric Wilcoxon framework to test group differences in antibody kinetics. For each participant, fold-change was defined as the difference in $\log_2$(MFI) between two timepoints

within each time segment (Cv–3M and 6–18M). For each comparison, Wilcoxon rank-sum tests with Bonferroni correction were applied to fold-change values.

To note, Figures 4 and 5, which focus on differences in kinetics between antigen and antibody response type, were updated to report Wilcoxon-based results, as paired within-individual analyses are more appropriate.

Full details of the Bayesian and Wilcoxon analyses are provided in the Methods, Supplementary Results, Supplementary Figures 12–13 (Bayesian model diagnostics), Supplementary Tables 3–5 (Bayesian variance components and convergence metrics), and Source Data (Wilcoxon test statistics). Modelled trajectories from these Bayesian models are visualized in the Extended Figures.

A new result section commenting the concordance of primary versus secondary antibody dynamics across LMM and BLMM and Bonferroni-corrected Wilcoxon tests (Supplementary Table 2) was added to the manuscript (lines 106-112):

> *"Antibody kinetics were primarily inferred using linear mixed-effects models (LMMs) following the experimental and analytic approaches illustrated in Supplementary Fig S2. Bayesian hierarchical modeling and non-parametric Wilcoxon analyses were applied as complementary approaches to assess robustness of observed trends. A summary of concordance across analytical frameworks for comparisons of infection groups is provided in Table S2 and discussed in the Supplementary Results. Unless otherwise stated, biological interpretations in the main text are based on the primary LMM framework, with divergences across methods explicitly noted when present."*

Thus our key results are consistent across inference frameworks and reflect underlying biology rather than model-specific artefacts.

5. **It would be helpful to include some discussion of any potential cohort effects—for instance, the timing or serotypes of prior exposures.**

A: We recognize the importance of discussing the impact of cohort effects. We expanded the Discussion of limitations as follows (lines 457-461):

> *"Some cohort effects exist that can impact these results. For the 2° cases, the serotype and timing of prior exposure may influence antibody dynamics, but this information was not available as cases were enrolled during illness. Infection histories from 2° individuals were unknown, and it is possible that antibody kinetics are further modulated by the exact number of flavivirus exposures or the serotype(s) of prior infections…It is important to note that our samples are derived from an endemic region with sustained DENV transmission, without other flavivirus immunization."*

6. **As far as I can tell, the homogeneous and cross-reactive compartments measured in the study will both capture cross-reactive antibodies, so I am not sure how relevant these comparisons are. I am also not sure about the use of DENV4 and especially DENV2 as proxies for cross-reactive antibodies. While DENV4 circulation is lower than the other serotypes, previous work by the authors has identified its asymptomatic circulation in Nicaragua.**

A: We acknowledge the need for clarification on our rationale, and explain it below and on lines 91-105.

Regarding the comparison of antibodies against homologous versus heterologous antigens, one may consider the example of primary DENV3 infection, such that DENV3 viral proteins (i.e., E, EDIII, or NS1) represent homologous antigens. As the Reviewer noted, antibodies that bind homologous antigens include both (a) type-specific (TS) antibodies targeting DENV3 uniquely and (b) cross-reactive (XR) antibodies targeting epitopes on DENV3 that are conserved across other serotypes. In contrast, antibodies from primary DENV3 case that bind heterologous antigens (i.e., DENV4 or DENV2 serotypes) must arise *exclusively* from XR antibodies, since these individuals have never been exposed to other serotypes. Consequently, heterologous antigens enable isolation

of the XR antibody compartment from the mixed (TS and XR) compartment observed on homologous antigens. While antibody dynamics studies commonly use homologous antigens, our study uniquely investigated whether these mixed antibody subsets show distinct kinetic behavior from XR-only antibody subsets.

Secondly, from the perspective of virion structure, the cross-reactive surface of a homologous antigen represents only a fraction of the total epitope landscape. It is therefore striking, and biologically meaningful, that the TS+XR response measured on the homologous antigen and the XR-only response measured on heterologous antigens demonstrate distinct longitudinal kinetic trajectories. Critically, this result suggests a hierarchy in the establishment of the DENV antibody response during the early phase (Cv–3M) and a selective long-term maintenance of specific antibody populations but not others (6–18M), which is a valuable immunological insight.

Also, we appreciate the opportunity to clarify how our prior work applies to the use of DENV4 antigens as proxies for heterologous antigens in this study. While we have previously detected rare inapparent primary DENV4 infections (2% among all inapparent infections) in the Nicaraguan population, DENV4 was not detected among *symptomatic primary* infections since the inception of the cohort in 2004, until 2022 (Bos, Zambrana et al., *Lancet Infect* Dis 2025 PMC11864988). Additionally, DENV4 did not circulate at epidemic levels in Nicaragua from the early 1990s until 2022 (Edgerton et al., Infect Genet Evol, 2020, PMC9359797). The oldest participant in our cohort was born in 1995. This suggests that our study population, from which the latest sample included was collected in 2013, should mostly be naïve to DENV4 and that DENV4 responses can be a good representation of the cross-reactive secondary response.

Regarding the use of DENV2 antigens, it is indeed likely that a substantial fraction of participants in the secondary infection group had experienced DENV2 previously. For this reason, DENV2 antigens were used as proxies for XR antibodies *only* for primary infections, where DENV2 binding can be interpreted unambiguously as XR-only.

7. Did all participants contribute to all sample time points? Why were samples collected during the acute phase not included to capture early changes in immune dynamics?

A: We recognize that sampling rigor impacts inferences. Eighteen-month samples for three participants (n=2 DENV1 primary; n=1 DENV3 primary) and one early convalescent sample (n=1 DENV1 primary) were not available. To preserve statistical power in group-level analyses, we imputed their missing value using the median of their corresponding group with matching serotype of infection, viral antigen, and antibody isotype/subclass. We now specify how missing values were handled in the Methods (lines 543-545):

> *"Values for four missing samples (n=3 at 18M, and n=1 EC) were imputed using the median of their corresponding group with matching serotype of infection, viral antigen, and antibody isotype/subclass."*

The acute-phase sample was intentionally excluded because our specific study question was to investigate the waning profile and longitudinal dynamics of the DENV antibody response following the peak of the response. We strategically chose the early convalescent timepoint as the initial measure because it is widely accepted as the window encompassing the peak antibody magnitude. The acute phase typically precedes this peak.

8. For traditional neutralisation assays, the reference virus will determine the magnitude of serotype-specific responses. Is this true of the proteins used for multiplex assays? If so, then this could cloud the homotypic versus cross-reactive inferences being made. Either way, it would be helpful to comment on this in the discussion.

A: Indeed, in neutralization assays, genotype matching can influence the magnitude of serotype-specific antibodies (Refs: Dowd et al., 2015, https://doi.org/10.1128/mbio.01559-15, and Young et al., 2020, https://doi.org/10.1016/j.chom.2020.04.007). This effect could, in principle, also influence binding assays; however, published studies demonstrating genotype-dependent differences in neutralization do not include corresponding binding data. Such effects were mainly described for monoclonal antibodies, whereas the measurements in this study rely on polyclonal sera which may target multiple epitopes, many of which are

conserved across genotypes, making it less sensitive to minor sequence variation. Thus, genotype-related shifts in binding magnitude are expected to be minimal.

Nevertheless, we calculated the amino-acid identity of the Nicaraguan DENV clinical isolate sequences with that of the E and NS1 used in our multiplex panel (see below Table R7). The E and NS1 antigens used here share 97% identity with the Nicaraguan DENV1 strain and 99–98% identity with the Nicaraguan DENV3 strain (the infecting serotypes in our study), and >98% identity for DENV2, DENV4 and ZIKV. We believe that these levels of homology are unlikely to impact the key findings on homotypic versus cross-reactive antibody responses.

Importantly, the key conclusions do not rely on absolute binding magnitude, but instead on decay rates. Even if minor magnitude differences were introduced by genotype mismatching, this would not affect the key observation that antibodies binding the antigen set of the same serotype (e.g., DENV4) exhibit change in kinetics over time.

Table R7: Amino-acid identity between the Nicaraguan DENV clinical isolate and Luminex antigens

	DENV1	DENV2	DENV3	DENV4	ZIKV
Genotype of Nicaraguan clinical isolate strains	V	III	III	II	-
Genotype of E and NS1 antigens strains	IV	V	III	II	-
E Identity	96.99%	98.25%	99.24%	98.75%	100%
NS1 Identity	97.15%	98.30%	98.01%	98.30%	99.72%

9. By design, this study included only clinical dengue, limiting inference that can be made about the relationship between antibody kinetics and clinical risk or protection. This should at least be commented on.

A: We thank the Reviewer for this insightful comment. We have now included this point in our discussion of limitations (lines 461-466, 473-475):

> *"Of note, our longitudinal sampling timepoint is up to 18 months post-infection, and longer-term dynamics on the scale of 3-4 years after dengue will be needed to evaluate antibody dynamics in the typical risk period for subsequent disease; such studies from our Nicaraguan cohort are being reported separately. This study did not include participant groups with differences in infection or disease outcome; thus, the relationship between antibody kinetics and clinical risk or protection must be further investigated…This study included hospital pediatric dengue cases, which may differ from the antibody dynamics of community infections with asymptomatic or mild disease"*

Reviewer #1 (Remarks on code availability):

10. I was not able to find the code using the link provided.

A: We apologize for the confusion. We have updated the link with the code for this study: https://zenodo.org/records/16743227

Reviewer #2 (Remarks to the Author):

Bos et al studied antibody responses to DENV and ZIKV E, EDIII, and NS1 antigens in serial blood samples collected over 18 months following symptomatic dengue in 79 children using a multiplexed bead-based immunoassay (105 measurements per blood sample). Antibody responses were compared over time, between the infecting virus and heterologous viruses (DENV and ZIKV), and by antigen, isotype, and IgG subclass based on linear mixed-effects modeling. The main findings were a) some DENV serotype-cross-reactive antibody responses increased rather than decreased from 6 to 18 months post-infection, b) responses to different viruses

and antigens did not all follow the same trends over time, c) a significant fraction of subjects were positive for IgA, IgM, and IgG3 at 18 months post-infection. The authors conclude that these data offer novel insights into flavivirus immunity and disease risk and have implications for vaccine design and serodiagnosis.

The large multiparameter dataset, longitudinal nature of the data, and well-characterized cohort of acute dengue cases all constitute substantial strengths of the work. There are, however, significant issues regarding the statistical methods and interpretation of differences. While the questions addressed in this analysis are of significant import, and the results of interest, the conclusions as currently stated are not adequately supported.

Major comments:

1. **Throughout the manuscript, the authors present mean values derived from statistical modeling. They calculated slopes (decay rates) for each interval from the derived mean values and transformed the slopes into estimated half-lives. Confidence intervals for mean values and slopes were estimated from 1000 bootstrap replicates of each model. Statistical inferences were mostly drawn from the comparisons of the calculated slopes although they are presented with the calculated half-life values. According to the figure legends, differences were considered significant at the p<0.05 level. The manuscript does not clearly state how p values were determined. In any event, it is not clear that this analytic approach is useful or justified, particularly since modeling was done separately for each response considered and the model allowed independent slopes for each interval between actual data points. A direct comparison of actual slopes for each subject would have more accurately accounted for the variances in the data.**

&

2. **Multiple statistical comparisons were performed, e.g., "separately for each antigen, viral response category, and antibody isotype and subclass" (lines 544-545), corresponding to at least 63 (3x3x7) different comparisons for each interval considered (C-3M, 3-6M, and 6-18M). The potential for type I error needs to be more carefully considered in the Discussion.**

A: We thank the Reviewer for these thoughtful comments. Because Questions 1 and 2 address overlapping aspects of our inference framework, we respond to them jointly below for clarity.

We tested significant differences in antibody *decay rate* across groups (i.e., infection history, antigen, and antibody isotype/subclass), not titers, throughout this study. Because p-value calculations for contrasts in non-parametric case-bootstrapped linear mixed models (LMM) can be unreliable and complex to calculate (*Davison AC, Hinkley DV. 1997. Bootstrap methods and their application. Cambridge, UK: Cambridge University Press*), we instead defined significance using the non-parametric bootstrap distribution of antibody decay rates. For each antibody subset and time interval, we summarized the bootstrap distribution of the decay rate with a point estimate and 95% confidence interval. A subset was classified as significantly waning or rising when its 95% confidence interval excluded zero. Differences between groups (e.g., primary vs secondary) were assessed using a confidence-interval overlap criterion, whereby a group was considered to differ from the reference if its 95% confidence interval did not overlap the reference group’s point estimate. All figure legends have been revised to reflect this approach, and references to “$p < 0.05$” have been removed throughout the manuscript.

The following statement was added to the Methods to clarify our approach (lines 587-593):

> *“Differences between groups were assessed using contrasts in decay rates obtained from a non-parametric bootstrap of individual trajectories. For each antibody subset and time interval, a subset was classified as significantly waning or rising when its 95% confidence interval excluded zero. In addition, within each stratum, differences between groups were evaluated using a confidence interval-overlap criterion, whereby a group was considered to differ from the reference group if its 95% confidence interval for the decay rate did not overlap the reference group’s point estimate.”*

We acknowledge the concern regarding multiple comparisons. As described above, our inference framework does not rely on asymptotic p-values but instead on non-parametric case (individual-level) bootstrap of antibody decay trajectories. This approach explicitly captures between-individual variability and uncertainty arising from the relatively small number of participants contributing to each antibody subset and time interval. Given the heterogeneity of immune responses and the limited effective sample size within strata, this case-bootstrap–based confidence interval approach is usually more conservative and robust than standard distribution-based regression inference.

To further assess robustness and limit the risk of type I error in this high-dimensional setting, we performed two complementary sensitivity analyses: (i) we fit a single unified Bayesian hierarchical linear mixed model (BLMM) with the same spline-based structure and weakly informative priors on fixed and random effects. This model jointly estimates all decay parameters within a multilevel framework, inducing partial pooling and shrinkage across antibody subsets and thereby reducing the risk of false positives due to overfitting or multiple testing. (ii) we implemented a model-independent non-parametric Wilcoxon framework to test group differences in antibody kinetics. For each participant, fold-change was defined as the difference in $\log_2$(MFI) between two timepoints within each time segment (Cv–3M and 6–18M). For each comparison, Wilcoxon rank-sum tests with Bonferroni correction were applied to fold-change values.

To note, Figures 4 and 5, which focus on differences in kinetics between antigen and antibody response type, were updated to report Wilcoxon-based results, as paired within-individual analyses are more appropriate.

Full details of the Bayesian and Wilcoxon analyses are provided in the Methods, Supplementary Results, Supplementary Figures 12–13 (Bayesian model diagnostics), Supplementary Tables 3–5 (Bayesian variance components and convergence metrics), and Source Data (Wilcoxon test statistics).

A new result section commenting the concordance of primary versus secondary antibody dynamics across LMM and BLMM and Bonferroni-corrected Wilcoxon tests (Supplementary Table 2) was added to the manuscript (lines 106-112):

> *"Antibody kinetics were primarily inferred using linear mixed-effects models (LMMs) following the experimental and analytic approaches illustrated in Supplementary Fig S2. Bayesian hierarchical modeling and non-parametric Wilcoxon analyses were applied as complementary approaches to assess robustness of observed trends. A summary of concordance across analytical frameworks for comparisons of infection groups is provided in Table S2 and discussed in the Supplementary Results. Unless otherwise stated, biological interpretations in the main text are based on the primary LMM framework, with divergences across methods explicitly noted when present."*

3. The general form of the statistical model provided (lines 548-550) incorporates a single primary factor A along with days post symptom onset, age, and sex as predictor variables. In this model, treatment of a binary variable, e.g., primary versus secondary infection, is straightforward. It is not clear how this formula was applied for comparisons across factors that were not binary, e.g., viral antigen or viral response, where 3 (or 4) values were possible, as is presented in several of the figures.

A: We recognize the need for additional detail on the model. The formula provided was a generalized example and varied across factors. We provided further explanation in the Methods as follows (Lines X):

> "This generalized formula varied across factors. For example, the viral antigen has three outcomes/categories, including envelope (E protein), E protein domain III (EDIII), and nonstructural protein 1 (NS1), and this was evaluated using the following formula:
>
> log2(MFI | B) =
>
> β0 +

$\beta 1 \cdot EDIII + \beta 2 \cdot NS1 +$

$\beta 3 \cdot DPSO + \beta 4 \cdot (DPSO - 100) + \beta 5 \cdot (DPSO - 200) +$

$\beta 6 \cdot (EDIII \times DPSO) + \beta 7 \cdot (EDIII \times (DPSO - 100)) + \beta 8 \cdot (EDIII \times (DPSO - 200)) +$

$\beta 9 \cdot (NS1 \times DPSO) + \beta 10 \cdot (NS1 \times (DPSO - 100)) + \beta 11 \cdot (NS1 \times (DPSO - 200)) +$

$\beta 12 \cdot Age + \beta 13 \cdot Sex + \mu\_participant + \varepsilon$

The antibody response type variable, which has four categories (homologous/infecting virus, DENV2-cross-reactive, DENV4-cross-reactive, or ZIKV-cross-reactive), was analyzed similarly."

4. The authors should include the serotype of the current infection (DENV1 versus DENV3) as a potential predictor variable in their analysis, given published evidence of significant differences in immune responses. In the Discussion, the authors should comment on any differences according to the serotype of infection.

A: The Reviewer raises an excellent point regarding the serotype of prior infection. Additional tests were conducted to evaluate differences in decay rates by serotype of primary and secondary dengue and did not identify any notable differences. A Bayesian model including interaction terms by infecting serotype only add ~2% of additional explained variability. As shown below (Reviewer Figure R1), antibody dynamics by infecting serotype across antibody features did not reveal notable serotype-specific pattern. This may be because DENV1 and DENV3 are the most closely related serotypes from the DENV serocomplex, as well as the limited power to detect serotype-specific effects in the available sample. Given the limited incremental explanatory value and the fact that only DENV1 and DENV3 infections were represented in this cohort, serotype was not retained in the primary models.

We mention this in the Discussion (lines 343-350):

"We additionally tested whether antibody kinetics differed according to the infecting DENV serotype (DENV1 versus DENV3). In this cohort, inclusion of infecting serotype explained little additional variability in antibody decay rates and did not alter the primary contrasts between infection histories or antibody response types. This likely reflects both the close antigenic relationship between DENV1 and DENV3 and limited power to detect serotype-specific effects in the available sample set. We therefore cannot exclude the possibility that antibody kinetics differ by infection serotypes, including those not incorporated in this study (e.g., DENV2 or DENV4); this should be addressed in future studies with broader serotype coverage."

Figure R1. Antibody kinetics stratified by infecting dengue serotype (DENV1 vs DENV3)

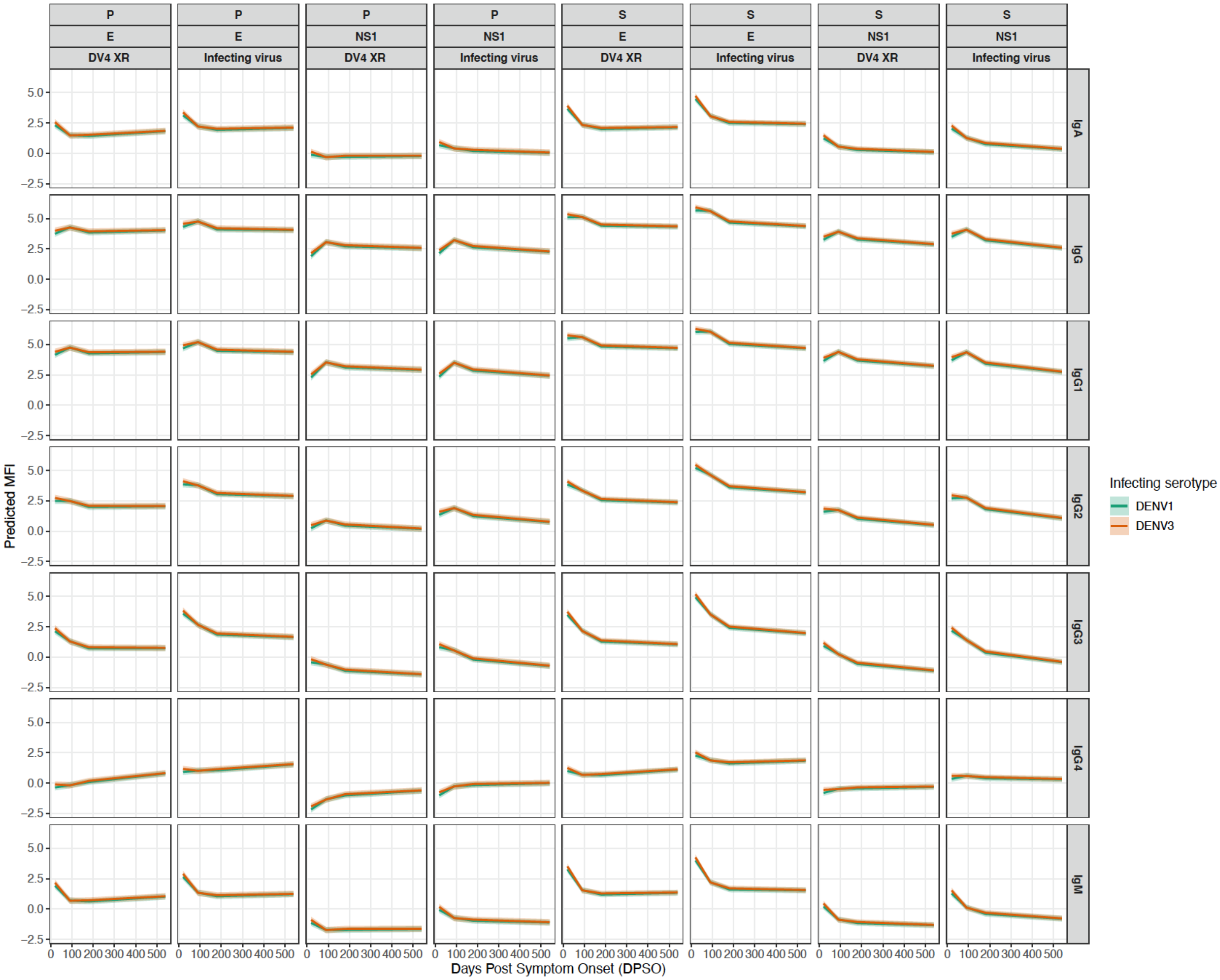

5. **The study design indicates (line 498) that blood samples were also collected at 12 months after symptom onset. Where these samples tested? Inclusion of these data would strengthen the statistical modeling between months 6 and 18.**

A: We acknowledge that 12-month sampling may strengthen the statistical modeling. Unfortunately, the availability of the 12-month sample for these participants was not included in the upfront selection criteria for these longitudinal sample sets and thus many 12-month samples are not available for further testing.

6. **In much of the manuscript, the authors equate MFI values to antibody levels, and several of the novel insights offered are based on this assumption. The authors acknowledge (e.g., lines 167-168) that MFI values may be affected by interactions between antibody subsets. However, this and other factors such as antibody affinity are not adequately considered as alternative explanations for observed response kinetics, e.g., a "rise" in IgG4 (line 433).**

A: We thank the Reviewer for this comment. To demonstrate that MFI values correspond with antibody levels, we conducted an additional experiment by testing a serially diluted DENV-binding mAb and two representative samples (see Reviewer Figure R2A below). The serial dilution curve of the known DENV-binding mAb (DV10) and polyclonal sera show that MFI tracks with the magnitude of antibody, such that a large MFI value can be

interpreted as a high level of DENV-binding antibody and a low MFI value can be interpreted as a low level of DENV-binding antibody.

With regards to the interaction between antibody subsets, from a technical perspective, the multiplex MFI values of binding antibody track well with the single-plex (i.e., single antigen) format (see Reviewer Figure R2B below). Multiplexed measurements of sera demonstrated only slightly lower MFI than single-plex, indicating multiplexing may slightly underestimate DENV-binding antibody level but with an equivalent dynamic range of detection to capture kinetics over time with the same approach.

We initially hypothesized that antibody interactions would impact inferences of waning antibodies more than rising antibodies, as the predominant and affinity-matured IgG may have masked the detection of minor isotypes like IgM or minor subclasses like IgG4. However, detection of these minority isotypes up to 18M suggests that this approach has sufficient excess antigen at the 1:800 dilution for sensitive antibody detection.

From an inferential perspective, the Reviewer brings up a good point about the potential for affinity to impact our inference of rising IgG4, which is a minor immunoglobulin in blood. We have added this valuable alternative inference to our discussion (Lines 422-425):

> *"Alternatively, the rise in DENV E-binding IgG4 antibodies may be due to long-term affinity maturation and production of IgG4 with greater affinity over time, resulting in higher MFI readouts. A rising trajectory can thus be a consequence of an increasing amount of DENV-binding immunoglobulin, as well as increasing affinity. Both possibilities are not mutually exclusive."*

Finally, the alternative of purifying immunoglobulin or isolating mAbs may make the results less physiologically relevant, as different antibody isotypes and subclasses co-exist *in vivo* as well and may presumably interact in blood too. The rigorous study of immune complexes in blood is outside the scope of this current study.

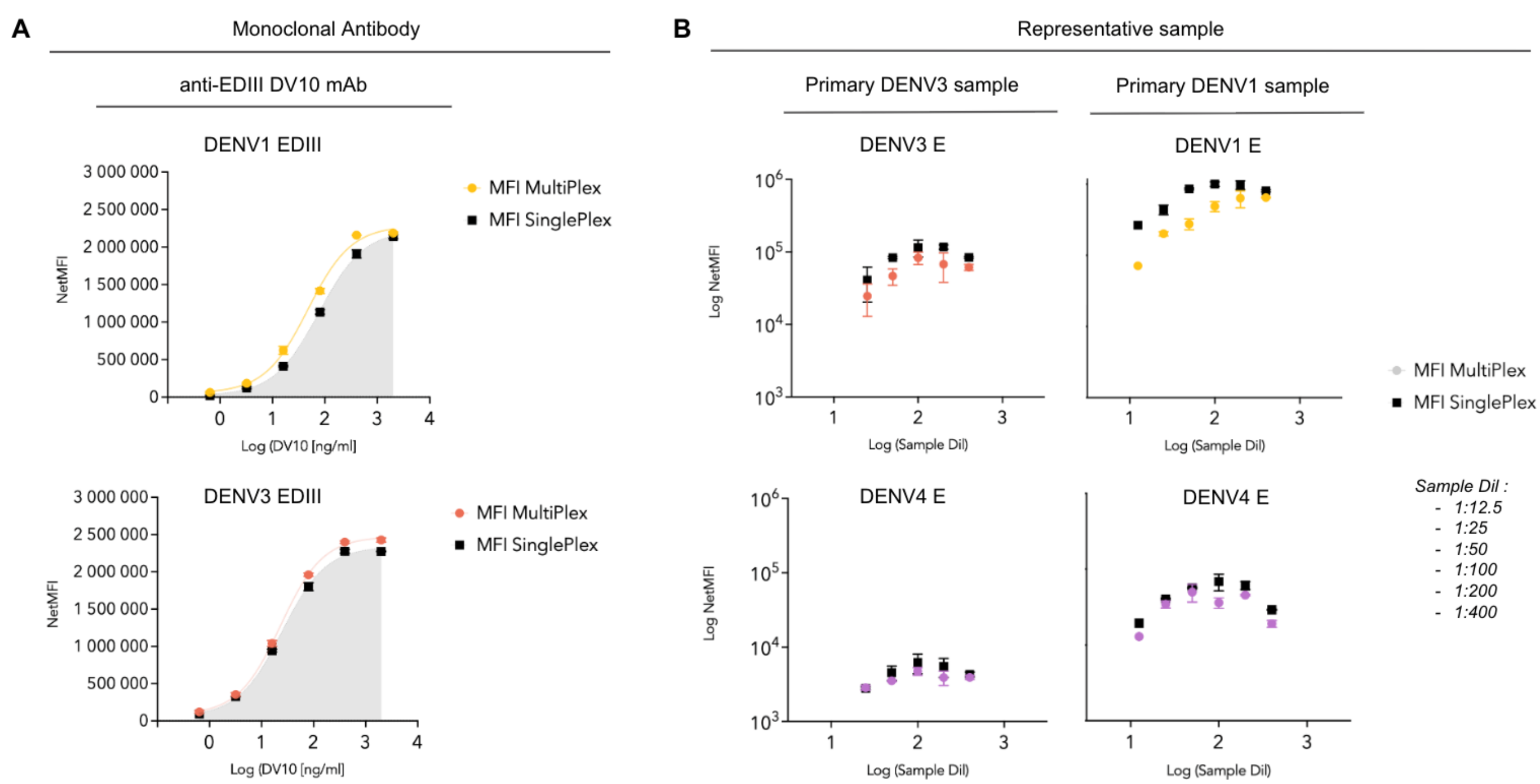

Figure R2. Antibody binding dose–response curves measured by single and multiplex assays.
A) Binding MFI dose-response curve of serially diluted DENV-binding monoclonal antibody DV10. Filled black squares represent readouts from the SinglePlex format and filled colored circles denote readouts from the MultiPlex format. B) Binding MFI dose-response curve of serially diluted plasma from a primary DENV3 and a

primary DENV1 case. Filled squares represent readouts from MultiPlex format. Net MFI is background subtracted.

7. It is evident from individual response data (Figures S2, S4, and S9) that a subpopulation of subjects showed large increases in some antibody responses between months 6 and 18. While the authors acknowledge (line 336) the possibility of re-exposure to DENV in their study population, this is not adequately considered as an alternative explanation for several of their novel conclusions.

A: We agree that distinguishing true long-term increases in antibody levels from subclinical boosting events is an important challenge when interpreting rising or stable antibody levels, particularly for cross-reactive IgG responses. We now clarify this point in the Discussion and explicitly acknowledge it as a limitation (lines 467-73):

> *"It is important to note that our samples are derived from an endemic region with sustained DENV transmission, without other flavivirus immunization. Importantly, although occasional boosting from unrecognized DENV exposures may contribute to stable or rising antibody levels in some subsets, the distinct long-term waning or rising patterns observed across specific antibody subsets suggest that boosting alone is unlikely to fully account for our main findings. However, boosting due to subclinical re-exposures cannot be ruled out. Antibody dynamics following a single 1° exposure in travelers from non-endemic regions may follow different trajectories."*

8. For several of the responses, MFI values below background are included in the statistical modeling. Conclusions drawn from these values are problematic.

A: We agree with the Reviewer that when antibody magnitudes are below the assay background, the biological interpretation of estimated slopes is limited if analyzed without taking the absolute magnitude into account. For this reason, now we do not interpret decay or rise rates when the average antibody magnitude is below the seropositivity cutoff (see below). That said, we intentionally retain below-background MFI values in the statistical modeling. The Luminex platform reports continuous fluorescence intensity values that reflect a combination of true low-level signal and instrument-level background noise. Excluding values below background would artificially truncate the response distribution, bias slope estimation, and distort uncertainty. Including these values allows the model to appropriately characterize random measurement noise and prevents upward bias in estimated antibody trajectories. To ensure transparency and avoid over-interpretation, we now do not present or interpret slope estimates for responses whose average magnitude is below the seropositivity threshold, as these slopes are not biologically meaningful even if they are statistically estimable.

All relevant figure legends have been updated to include:

> *"For time segments in which the modeled antibody isotype/subtype trajectory remained at or below zero over the entire interval, the corresponding half-life estimates and CI were omitted from half-life panels to avoid misleading interpretation."*

9. The authors highlight "long-lasting antiviral IgA, IgM, and IgG3 antibodies" and suggest that these findings have implications for the serological diagnosis of dengue. In their analysis, seropositivity at 18 M was defined as responses exceeding the mean + 3 SD MFI of five DENV-naïve samples included on each plate. In at least several cases, these positive responses are very low in magnitude. More extensive testing of the assay with a larger number of control samples would be needed to establish the practicality of this cutoff for clinical diagnosis. This limitation should be considered in the Discussion.

A: The Reviewer makes a good point. To clarify, this multiplex assay was intended for research, and we are not proposing this multiplex assay for clinical diagnosis; thus, a cut-off for clinical diagnosis was not provided. This assay is sensitive and designed to understand antibody responses to DENV infection. The point is that use of DENV-binding IgA, IgM, or IgG3 as a hallmark of recent infection may be problematic, as a fraction of the study group demonstrated positivity for these antibodies up to 18 months after infection. This may be relevant to consider for novel diagnostic approaches with more sensitivity than current tests. To address the Reviewer's comment, we have removed "serodiagnosis" as an implication from the abstract.

Minor comments:

10. Primary versus secondary infections were defined based on the serological response to infection, in keeping with normal practice. However, this should be considered in interpreting the differences in antibody kinetics, particularly for the first interval (C-3M).

A: The Reviewer makes an excellent point. To provide more detail, primary versus secondary serostatus was defined by DENV iELISA, which contains a mix of 4 crude viral antigens from infectious mouse brain or cell culture homogenate and a composite measure of all antibody isotypes as one value. In contrast, the measurements in this study were performed using a more sensitive multiplex assay, which enabled quantification of 7 antibody isotypes/subclasses binding with 15 purified recombinant protein viral antigens. Thus, different platforms are involved in case definition and generation of analytical data.

To directly address the Reviewer's concern, we evaluated the concordance between iELISA-based classification and Luminex measurements using IgG1 responses to the E protein, the most immunogenic DENV antigen and the most abundant antibody subclass following infection. As shown in the density distributions and ROC analysis (Reviewer Figure R3), IgG1 E-protein responses measured by Luminex clearly discriminate primary from secondary infections, with excellent agreement with iELISA-based classification (Youden-derived threshold [net MFI = 1,624,467], specificity ≈0.97 and sensitivity ≈0.87).

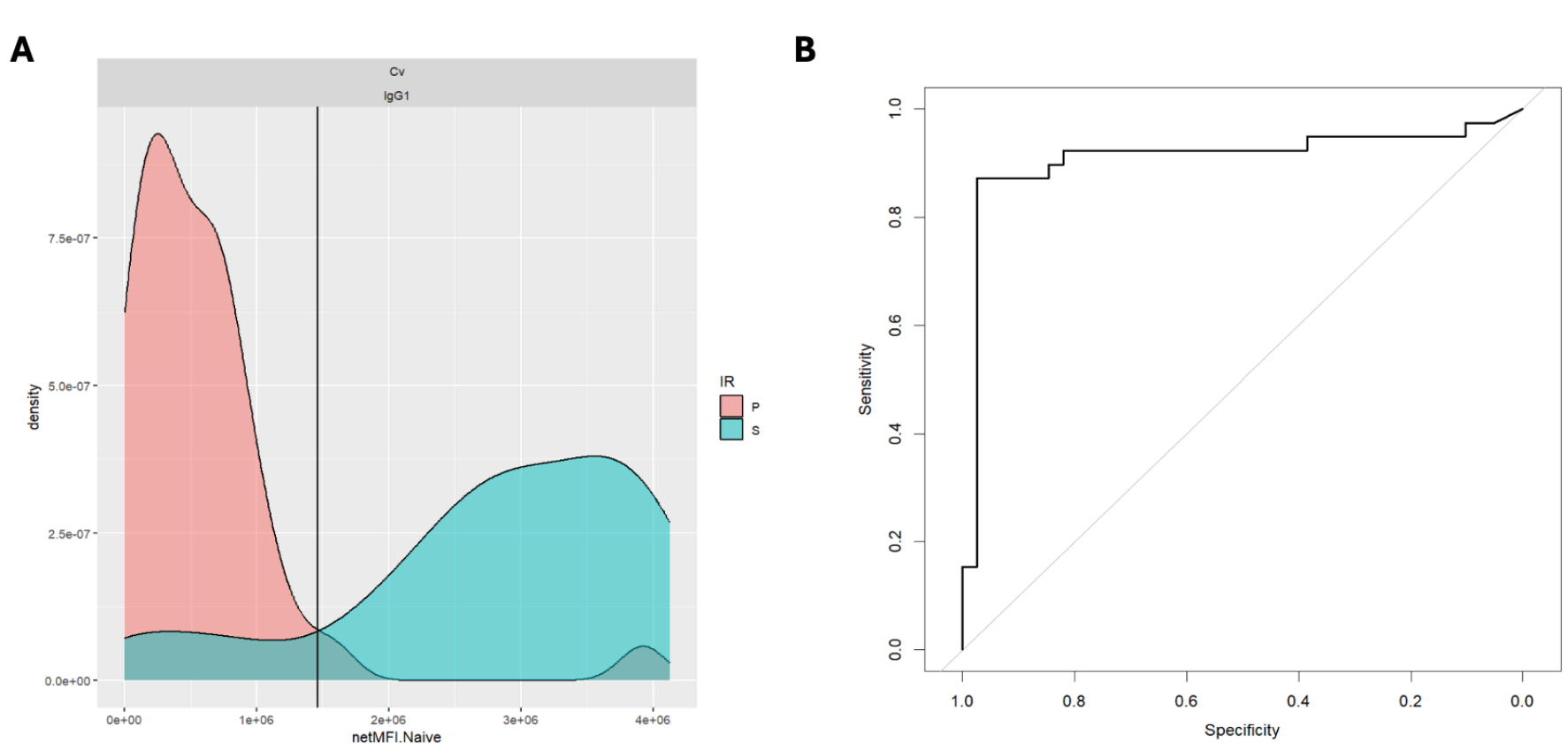

Figure R3. Concordance between iELISA-based serostatus classification and Luminex IgG1 E-protein measurements.

(A) Kernel density distributions of E-protein–specific IgG1 net MFI measured by Luminex during the early convalescent period (Cv) in individuals classified as primary (P, pink) or secondary (S, teal) DENV infection based on iELISA. The vertical black line indicates the optimal discrimination threshold derived from the Youden index. (B) Receiver operating characteristic (ROC) curve evaluating the ability of Luminex-measured E-specific IgG1 responses to distinguish primary from secondary infections as defined by iELISA. The diagonal gray line denotes random classification.

11. Figures showing the half-life of antibody responses (e.g., Figure 1B) are confusing and difficult to interpret since the y axis scale is uninformative and crosses -inf. It appears that the graphs are actually decay rates with values for y axis labels transformed to half-lives. Labeling these as decay rates instead would more accurately depict the information.

A: We thank the Reviewer for raising this point and appreciate the opportunity to clarify the presentation. As described explicitly in the Methods (lines), the primary quantities estimated by the models are decay rates expressed as daily changes in $\log_2$(MFI). These rates were subsequently transformed into half-life values.

Under this convention, "a half-life of zero implies an infinite half-life (no net change in antibody concentration), negative values represent a rising antibody response ('negative half-life'), and positive values reflect waning antibody concentrations ('positive half-life')," as defined in the Methods (lines). Accordingly, values approaching −Inf correspond to decay rates near zero and indicate stable antibody kinetics. This interpretation is also stated explicitly in the Figure Legends:

> "stable decay rate with non-estimable infinite (-Inf) $t_{1/2}$ (i.e., Not significant). Solid colors are significantly rising (-) or waning (+; shaded area) antibody kinetics."

12. The investigators tested sera at 4 dilutions, but present the analysis of data form the 1:800 dilution only. For their most novel findings, did other serum dilutions show similar results?

A**:** The Reviewer raises an important point regarding the use of serial serum dilutions. Samples were tested at four dilutions (1:50, 1:200, 1:800, and 1:3200). At the lower dilutions (1:50 and 1:200), most samples from secondary DENV infections exhibited saturating MFI values, consistent with a prozone effect, which precluded reliable quantitative comparison across groups. At the highest dilution (1:3200), signal intensity was insufficient to consistently detect lower-abundance antibody isotypes and subclasses (notably IgM and IgG3), even at early convalescent time points (e.g., 3 months post-infection), when these responses are biologically expected to be detectable.

As a result, the 1:800 dilution represented the only dilution that provided a robust and consistent dynamic range across both primary and secondary infection groups, all antibody isotypes/subclasses, and all analyzed timepoints. For this reason, all quantitative analyses presented in the manuscript were performed using data from the 1:800 dilution.

13. Line 207- Figure 4E is incorrect cited in the text. The figure for the interval 6-18M does not show a statistically significant difference.

A: We thank the Reviewer for this correction. The citation has now been corrected to refer to Figure 4F, which shows the 6-18M period.

14. Figure 7- y axis values presented in panel (a) do not appear to match corresponding results in Figure 3. Panel (b) is incorrectly described in the legend as resulting from modeling; this figure shows individual subject values. For panel (c) none of the subjects is classified as "stable" (noted in the legend).

A: We are grateful to the Reviewer for identifying this discrepancy. We inadvertently plotted non-background subtracted MFI in panel A. With regards to Panel B, the figure legend has been corrected to indicate that these are individual subject values. With respect to Panel C, the figure legend has been corrected to only indicate rise and wane to match the figure.

*"**Figure 7. Interindividual variability in DENV4 envelope cross-reactive antibody magnitude and 6-to-18-month post-infection antibody kinetics.** DENV4 E-binding antibodies were measured as log_2 of mean fluorescence intensity (MFI) after background subtraction (BG; shaded area) in both the post-primary (P) and post-secondary (S) dengue groups. **a.** Distribution of the XR anti-E antibody magnitude across individuals at 18 months post-infection by infection group. **b.** Distribution of fold-change in XR anti-E antibody binding between 6-18M across individuals by infection group. **c.** Proportion of the post-primary versus post-secondary group with either rising or waning XR anti-E antibody kinetics from 6-18M for each antibody isotype and IgG subclass. Each dot represents an individual, and color indicates 6-18M antibody kinetics as rising (orange) or waning (green)."*

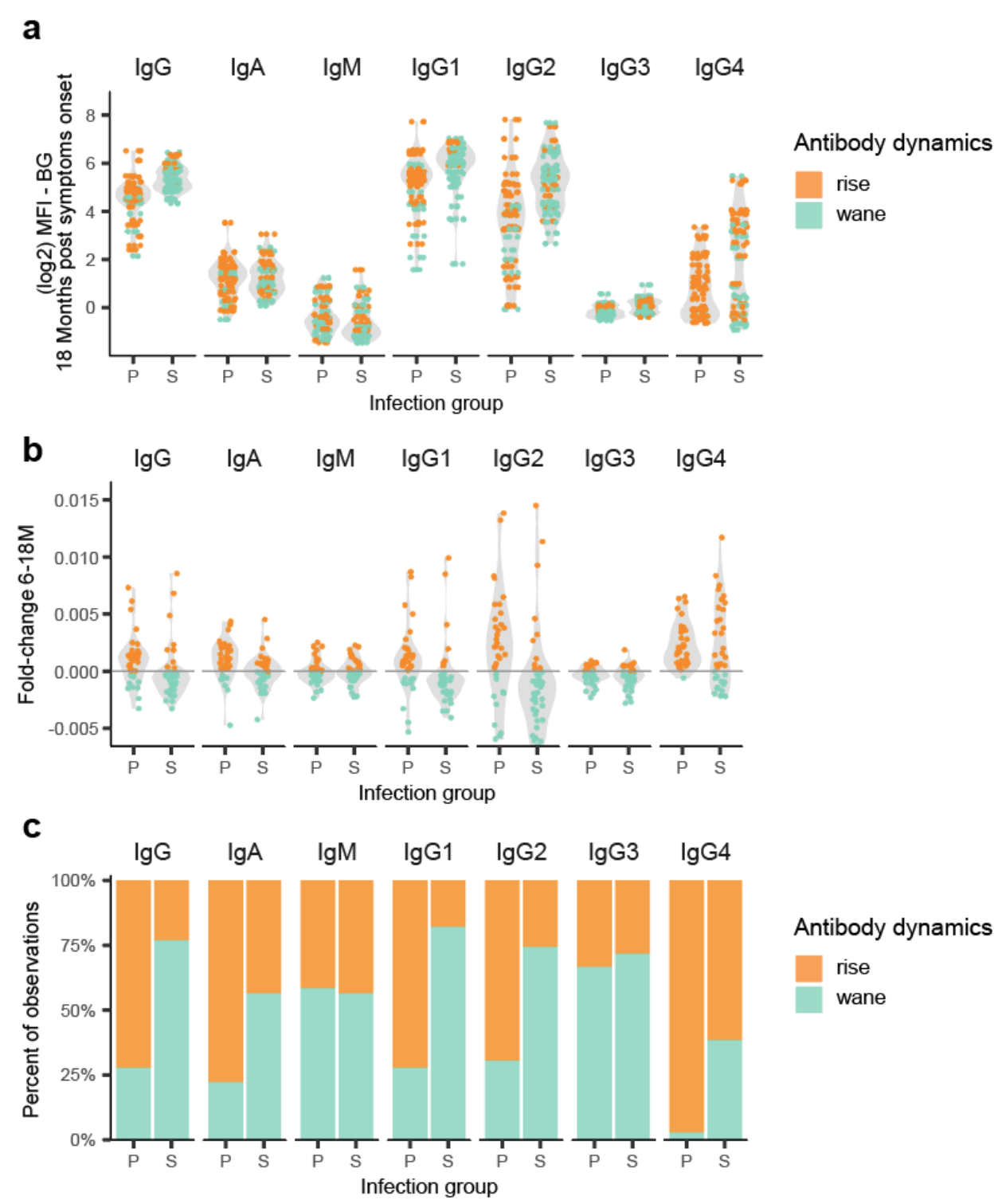

15. Line 574- "half-life of zero" is incorrect.

A: We agree and have corrected our figures and text to read "infinite" and not zero.

Reviewer #3 (Remarks to the Author):

What are the noteworthy results?

- The authors describe the kinetics and breadth of different antibody responses to different dengue virus proteins and domains within proteins following primary and secondary D1 and D3 infections. There is specific attention to defining the rise and fall of homotypic and cross reactive antibody profiles. Rise in cross reactive IgG antibodies over time (not waning) was a unique finding as was the persistence of IgM and IgA antibody populations.

Will the work be of significance to the field and related fields? How does it compare to the established literature? If the work is not original, please provide relevant references.
- The work is relevant to the dengue immunology field and describes specific immunologic profiles over time which few other groups have described. The concept of longitudinal profiling is not new but the measured endpoints and breadth of the same are unique.

A: We thank the Reviewer for their positive comments.

Does the work support the conclusions and claims, or is additional evidence needed?

- The authors lay the foundation of the significance of their work on the theory of ADE and it being the reason why people develop severe disease. ADE is a theory and should not be taken as a proven or definitive immunopathologic mechanism for severe dengue. Building off of the ADE theory as fact and using this as a bridge between the authors findings and speculations of mechanisms of severe disease weakens the conclusions. It has been a significant challenge for the field to relate in time and space a baseline immunologic profile, dengue virus infection, and subsequent virologic and immunologic endpoint which are significantly associated with particular clinical outcomes as described in the ADE theory. The manuscript would be strengthen with less speculation and more attention paid to historic data using different methods (neut ab) and how their data differs (rise and fall of cross reactive abs in particular).

A: This is a fair point. There is good consensus in the dengue research field on the role of cross-reactive DENV antibodies in worsening subsequent dengue disease and enhancing viral replication, as this has been demonstrated *in vivo*, *in vitro*, and with association analyses within cohort studies where antibody titer mediates risk in the following studies:

1. Halstead, S. B., Nimmannitya, S., Cohen, S. N. Observations related to pathogenesis of dengue hemorrhagic fever. IV. Relation of disease severity to antibody response and virus recovered. Yale J. Biol. Med. 42, 311–328 (1970).
2. Halstead, S. B., O'Rourke, E. J. Dengue viruses and mononuclear phagocytes. I. Infection enhancement by non-neutralizing antibody. J. Exp. Med. 146, 201–217 (1977).
3. Sangkawibha, N., Rojanasuphot, S., Ahandrik, S., Viriyapongse, S., Jatanasen, S., Salitul, V., Phanthumachinda, B., Halstead, S. B. Risk factors in dengue shock syndrome: A prospective epidemiologic study in Rayong, Thailand. I. The 1980 outbreak. Am. J. Epidemiol. 120, 653–669 (1984).
4. Guzman, M. G., Alvarez, M., Halstead, S. B. Secondary infection as a risk factor for dengue hemorrhagic fever/dengue shock syndrome: An historical perspective and role of antibody-dependent enhancement of infection. Arch. Virol. 158, 1445–1459 (2013).
5. Katzelnick, L. C., Gresh, L., Halloran, M. E., Mercado, J. C., Kuan, G., Gordon, A., Balmaseda, A., Harris, E. Antibody-dependent enhancement of severe dengue disease in humans. Science 358, 929–932 (2017).
6. Salje, H., Cummings, D. A. T., Rodriguez-Barraquer, I., Katzelnick, L. C., Lessler, J., Klungthong, C., Thaisomboonsuk, B., Nisalak, A., Weg, A., Ellison, D., Macareo, L., Yoon, I. K., Jarman, R., Thomas, S., Rothman, A. L., Endy, T., Cauchemez, S. Reconstruction of antibody dynamics and infection histories to evaluate dengue risk. Nature 557, 719–723 (2018).
7. O'Driscoll, M., Buddhari, D., Huang, A. T., Waickman, A., Kaewhirun, S., Iamsirithaworn, S., Khampaen, D., Farmer, A., Fernandez, S., Rodriguez-Barraquer, I., Srikiatkhachorn, A., Thomas, S., Endy, T., Rothman, A. L., Anderson, K., Cummings, D. A. T., Salje, H. Maternally derived antibody titer dynamics and risk of hospitalized infant dengue disease. Proc. Natl. Acad. Sci. U.S.A. 120, e2308221120 (2023).
8. Halstead, S. B., Porterfield, J. S., O'Rourke, E. J. Enhancement of dengue virus infection in monocytes by antibody. J. Exp. Med. 146, 201–217 (1977).
9. Kliks, S. C., Nisalak, A., Brandt, W. E., Wahl, L., Burke, D. S. Antibody-dependent enhancement of dengue virus growth in human monocytes as a risk factor for dengue hemorrhagic fever. Am. J. Trop. Med. Hyg. 40, 444–451 (1989).
10. Goncalvez, A. P., Engle, R. E., St. Claire, M., Purcell, R. H., Lai, C.-J. Monoclonal antibody–mediated enhancement of dengue virus infection in vitro and in vivo and strategies for prevention. J. Virol. 81, 8372–8380 (2007).

11. Dejnirattisai, W., Jumnainsong, A., Onsirisakul, N., Fitton, P., Vasanawathana, S., Limpitikul, W., Puttikhunt, C., Edwards, C., Duangchinda, T., Supasa, S., et al. Cross-reacting antibodies enhance dengue virus infection in humans. Science 328, 745–748 (2010).

Nevertheless, we agree that antibody-mediated enhancement of disease can be due to multiple immunopathological mechanisms, one of which being the classical ADE theory (see also next response) and others being NS1-IgA leading to excess neutrophil activation and Fcγ IIIa-activating antibodies. Thus, to address the Reviewer's comment: 1) several statements on ADE have been revised to be inclusive of diverse antibody-mediated immunopathologies and clarify the foundation for this work (see below; in addition to the five specific suggestions from the Reviewer that were accepted in subsequent responses); 2) a Discussion paragraph was substantially revised to further contextualize how these findings fit with historical data on the rise and fall of antibodies (see below). To clarify, the main goal of this study was to comprehensively understand the distinct DENV-binding antibody subsets present and estimate their different kinetics up to 18 months post-infection.

Revisions to statements underlined

♦ Abstract

Line 21- 33:

"One key issue is that certain levels and subsets of cross-reactive antibodies have been associated with enhanced disease during subsequent infection with a different DENV serotype. To understand the heterogeneity of DENV antibody responses and delineate their distinct kinetics, we defined the magnitude and kinetics of 84 antiviral antibody subsets (by isotype, subclass, antigen, and cross-reactivity) after primary versus secondary dengue, using longitudinal samples collected <1, 3, 6 and 18 months post-symptom onset from a pediatric hospital study in Nicaragua….Thus, the cross-reactive subset of post-primary anti-DENV antibody responses demonstrate distinct kinetics from overall DENV-binding antibodies as well as from secondary immune responses, which have implications for the outcome of subsequent DENV infections."

♦ Introduction

Lines 46-48:

"Among several mechanisms for antibody-mediated immunopathogenesis, the theory of antibody-dependent enhancement (ADE) posits that antibodies facilitate viral entry into immune cells via Fcγ receptors, leading to increased viral replication, immune activation, and disease severity."

Lines 72 (removed "and protective roles"):

"However, limited information exists regarding the long-term persistence of these lower-abundance antibodies."

Lines 73-74:

"In this study, we sought to understand the heterogeneity of DENV-binding antibody subsets and their distinct kinetics post-primary versus post-secondary dengue."

♦ Discussion

Lines 441-42, :

"Such heterogeneity may have important implications for long-term immunity by delineating the antibody population into distinct response groups."

"Altogether, these findings indicate that antibodies targeting different epitopes may exhibit distinct kinetics. If epitope-specific antibodies have distinct dynamics, it is possible that protective and enhancing antibodies can have different dynamics. For example, the stoichiometry of virion occupancy by antibodies can shift in vitro phenotypes from virus neutralization to enhancement of viral replication. Thus, the qualitative composition of cross-reactive antibody subsets (e.g., the epitopes they recognize in human blood) may have implications for

disease protection and risk, and our findings suggest that the composition of the cross-reactive antibody pool is dynamic."

Removed entirely:

"This is particularly relevant, as long-lasting antibody subsets may contribute to protection versus pathology in subsequent infection"

"Given that low to intermediate levels of XR IgG induced by 1° flavivirus infections are associated with an increased risk of subsequent severe dengue disease,[8–10] our data up to 18 months post-infection suggest an alternate perspective on how this may happen due to a low level of rising, rather than waning DENV XR antibodies"

Revised paragraph in the Discussion (Lines 310-335)

"Numerous historical studies show short-term cross-protection against disease with other DENV serotypes that lasts up to ~2 years, suggesting that cross-protection is temporary and wanes (Ref 1–8). While DENV-binding and neutralizing antibodies certainly wane from their peak post-primary response in early convalescence, as in the EC-3M and 3-6M periods from this and other studies (Ref 9–11), the underlying assumption has been that antibodies continue to wane on the scale of years, into the period of subsequent risk for severe dengue, which occurs 3-6 years later. Yet, we observed a significant rise in XR E-IgG, particularly the IgG1 and IgG2 subclasses, from 6 to 18 months post-1° DENV infection. This finding contrasts with prior reports on declining antibody levels.[44] Several factors may explain this discrepancy. First, differences in assays used and in statistical power, as well as the modeling approach, may have impacted kinetic changes detected in prior studies. Second, aggregation of antibody responses without distinction of DENV homotypic versus XR antibodies might have masked divergent trends. Specifically, we observed stable post-1° long-term total anti-E IgG (i.e., against the infecting serotype) but a rising XR antibody subset; thus, we infer that the type-specific antibody response may wane. In line with our finding, several prior studies show that post-primary heterotypic DENV neutralizing antibodies, heterotypic DENV hemagglutination-inhibition antibodies, and DENV E and fusion loop binding antibodies rise post-primary and wane post-secondary dengue (Ref 9,10,12–14). Prior studies with longer-term data suggest that this rise may stabilize on the scale of multiple years post-primary DENV infection (Ref 9,10). Together, this indicates that rising post-primary antibodies is a reproducible finding across distinct measurements and specifically observed in the heterotypic antibody subset. Our study supports these prior findings and additionally contributes that E-binding cross-reactive IgG1, IgG2, and IgA rise. Moreover, our data indicate that XR epitopes on E domains I/II, but not E domain III, contribute to the post-1° rise in XR E-IgG, as XR E-IgG rises post-1° infection but XR EDIII-IgG remains stable. While prior studies show that a low or intermediate titer of DENV-binding iELISA antibody is associated with severe disease in multiple cohorts, it remains unknown whether a 6-18M rising antibody kinetic has the potential to contribute to this (Ref 10,15)."

1. Sabin, A. B. Research on dengue during World War II. Am J Trop Med Hyg 1, 30–50 (1952).
2. Endy, T. P. et al. Determinants of Inapparent and Symptomatic Dengue Infection in a Prospective Study of Primary School Children in Kamphaeng Phet, Thailand. PLoS Negl Trop Dis 5, e975 (2011).
3. Anderson, K. B. et al. A Shorter Time Interval Between First and Second Dengue Infections Is Associated With Protection From Clinical Illness in a School-based Cohort in Thailand. J Infect Dis 209, 360–368 (2014).
4. Reich, N. G. et al. Interactions between serotypes of dengue highlight epidemiological impact of cross-immunity. J R Soc Interface 10, (2013).
5. Salje, H. et al. Revealing the microscale spatial signature of dengue transmission and immunity in an urban population. Proc Natl Acad Sci U S A 109, 9535–9538 (2012).
6. López, L., Paul, R. E., Cao-Lormeau, V. M. & Rodó, X. Considering waning immunity to better explain dengue dynamics. Epidemics 41, 100630 (2022).
7. Montoya, M. et al. Symptomatic versus inapparent outcome in repeat dengue virus infections is influenced by the time interval between infections and study year. PLoS Negl Trop Dis 7, (2013).

8. Bhoomiboonchoo, P. et al. Sequential dengue virus infections detected in active and passive surveillance programs in Thailand, 1994-2010 Disease epidemiology - Infectious. BMC Public Health 15, 250- (2015).
9. Katzelnick, L. C. et al. Dengue and Zika virus infections in children elicit cross-reactive protective and enhancing antibodies that persist long term. Sci Transl Med 13, (2021).
10. Salje, H. et al. Reconstruction of antibody dynamics and infection histories to evaluate dengue risk. Nature 2018 557:7707 557, 719–723 (2018).
11. Clapham, H. E. et al. Dengue Virus (DENV) Neutralizing Antibody Kinetics in Children After Symptomatic Primary and Postprimary DENV Infection. J Infect Dis 213, 1428–1435 (2016).
12. Clapham, H. E., Cummings, D. A. T. & Johansson, M. A. Immune status alters the probability of apparent illness due to dengue virus infection: Evidence from a pooled analysis across multiple cohort and cluster studies. PLoS Negl Trop Dis 11, e0005926 (2017).
13. Katzelnick, L. C., Montoya, M., Gresh, L., Balmaseda, A. & Harris, E. Neutralizing antibody titers against dengue virus correlate with protection from symptomatic infection in a longitudinal cohort. Proc Natl Acad Sci U S A 113, 728–733 (2016).
14. Lai, C. Y. et al. Analysis of Cross-Reactive Antibodies Recognizing the Fusion Loop of Envelope Protein and Correlation with Neutralizing Antibody Titers in Nicaraguan Dengue Cases. PLoS Negl Trop Dis 7, e2451 (2013).
15. Katzelnick, L. C. et al. Antibody-dependent enhancement of severe dengue disease in humans. Science (1979) 358, 929–932 (2017).

Are there any flaws in the data analysis, interpretation and conclusions? Do these prohibit publication or require revision?

- The findings are interesting and hypothesis generating but less informative as it relates to immunopathologic mechanisms or risk of severe disease outcomes. A revision with an expanded focus on historic data (they do discuss some) and the differences between those data and the authors data would be welcome.

A: We recognize the Reviewer's suggestion to expand discussion of how our findings relate to immunopathology. The following concepts regarding potential relationships to immunopathology were discussed based on the data of post-primary DENV-binding antibodies:

1. Rising and low levels of cross-reactive (XR) E-IgG1 in relation to risk for ADE (Lines 333-35).
2. Presence of E-IgA may be associated with protection due to antiviral functions of DENV neutralization and/or interference with ADE (Lines 351-54).
3. Antibodies binding non-EDIII XR epitopes on E in relation to risk for subsequent ADE (Lines 331-33).
4. Presence of XR NS1-IgA in relation to subsequent neutrophil pathogenesis (Lines 372-73).
5. Composition (i.e., relative amounts) of cross-reactive antibody subsets in relation to subsequent antibody-mediated immunopathogenesis (Lines 408-411).
6. Presence of lasting E IgM and IgG4 in some people in relation to subsequent complement-based virolysis or anti-inflammatory antibody effector function (Lines 417-21).
7. Inter-individual variation (i.e., responding versus non-responding individuals) as a factor in subsequent immunopathogenesis (Lines 436-38).

To clarify, the study does not include groups of individuals differing by clinical outcome and thus, statements on the mechanism of risk of severe disease can only be speculative and we prefer not to overstate a specific mechanism of immunopathology. Thus, the following was added to the Discussion of limitations (Line):

> *"This study did not include participant groups with differences in infection or disease outcome; thus, the relationship between antibody kinetics and clinical risk or protection must be further investigated."*

Please note the above response with regards to an expanded discussion of historical data.

Is the methodology sound? Does the work meet the expected standards in your field?
- Methodology is sound and meets standards.

A: We thank the Reviewer for acknowledging the soundness of our methodological approach.

Is there enough detail provided in the methods for the work to be reproduced?
- Many of the assays have been described before but there was at least one reference which did not seem to track with the text (REF 46). Multiplex assay is described well. Stats review is required.

A: We thank the Reviewer for indicating this discrepancy. The reference has been corrected: *Balmaseda A, Hammond SN, Pérez L, Tellez Y, Saborío SI, Mercado JC, Cuadra R, Rocha J, Pérez MA, Silva S, Rocha C, Harris E. Serotype-specific differences in clinical manifestations of dengue. Am J Trop Med Hyg. 2006 Mar;74(3):449-56. PMID: 16525106.*

Abstract:
"can enhance disease"
- More accurate to say "have been associated with…"

A: We agree. This has now been revised as per the Reviewer's suggestion in Line 21.

Introduction:
"lack of therapies"
- Evidenced based and highly effective therapeutic algorithms have been available for decades. Perhaps "DENV-specific" or "anti-viral" therapies is more accurate.

A: The Reviewer makes a valid point. This has been revised to include "DENV-specific anti-viral" (Line 40).

"Understanding immune responses is critical"
- Perhaps an overstatement. Safe and effective vaccines have been licensed and deployed into use without detailed understanding of immunogenicity and its association with clinical outcomes. Authors should make the case for why dengue is different (immunopathology of disease for example).

A: We recognize the Reviewer's point on distinct challenges due to immunopathology and have revised the statement (line 41-42):

> *"Given the potential for antibody-mediated immunopathology, understanding the antibody response to DENV infection is critical."*

"which can exacerbate infection"
- Unclear what this means? Are the authors making a virologic, immunologic, or clinical observation? The references provided use in vitro and small animal studies to explain a complex immunopathologic processes which occurs in humans and result in various clinical phenotypes. Qualifying the statement to be less conclusive seems appropriate.

A: We acknowledge the Reviewer's nuanced perspective, and have revised the statement (Line 45):

> *"...which have the potential to exacerbate infection…"*

"stable long-term antibody levels"
- What type of antibodies? The remainder of that paragraph requires clarification as to what type of antibodies the authors are referring to.

A: We appreciate the opportunity to clarify the point. Specifically, we referred to DENV binding antibodies detected via the DENV inhibition ELISA. The statement has been clarified in Lines 56-57:

"DENV-binding inhibition ELISA and neutralizing antibody levels."

Results:

"post-primary"

- How was primary and secondary defined?

A: Primary and secondary dengue were classified based on the DENV iELISA titer at early convalescence, as follows in the methods section (Lines 503-07)

"A primary DENV infection is classified by an iELISA titer[EH6.1] below 2560 in convalescent-phase samples; conversely, 2° infections are indicated by titers of 2560 or higher in convalescent-phase samples."

"Since DENV E-binding XR antibodies play"

- Are believed to play or are associated with may be more accurate phrasing.

A: We agree. This has now been revised according to the Reviewer's suggestion (Line 160-61):

"Since DENV E-binding XR antibodies are associated with subsequent immunopathology, we evaluated if this subset demonstrates distinct kinetics."

"enhancement or protection"

- Enhancement or protection of what? Virologic, immunologic, clinical outcomes? Recommend clarifying what is specifically being described.

A: We appreciate the Reviewer's suggestion to strengthen the point. The "enhancement or protection" phrase has been removed in response to a different comment, and the current statement is revised as follows (Lines 160-61):

"Since DENV E-binding XR antibodies are associated with subsequent immunopathology, we evaluated if this subset demonstrates distinct kinetics."

"outside the DENV1-4 serocomplex (ZIKV-XR"

- What was the dengue immune profile of the individuals who contributed ZIKV XR samples? Were they dengue naïve? If not, was the infecting DENV serotype(s) known? Could the timing of DENV infection in relation to ZIKV infection in these folks impact assay measurements?

A: The Reviewer raises a good point. To clarify, all the samples included in this study were from dengue cases that occurred before the introduction of ZIKV in Nicaragua. Thus, none of the individuals in this study experienced ZIKV infection. ZIKV-binding antibodies were assessed from these DENV-immune individuals to understand the trajectory of ZIKV cross-reactive antibodies elicited during DENV infection.

Individuals were enrolled in the hospital during dengue disease, not the cohort study. Each individual in the study was a primary or secondary case with either DENV1 or DENV3 infection. Each individual was sampled up to 18 months after their DENV infection, but their infection history before this infection is not known as they were not enrolled in the study prior to the infection sampled here.

"seropositivity impacts protection on the population level"

- I think this requires explanation. We have seen seropositivity NOT associated with protection in several vaccine trials. A short description and references would strengthen the manuscript.

A: We recognize the Reviewer's nuanced point regarding seropositivity, especially considering vaccine trials. To clarify, seropositivity in and of itself does not confer protection, as Halstead's historical studies from the 1970s indicate that prior immunity to dengue is a risk for subsequent severe dengue. The observed failure of seropositivity to correlate with protection in certain vaccine trials is hypothesized to be due to an imbalance in neutralizing titers or the induction of sub-neutralizing antibodies, a scenario distinct from the broad, functional antibody profile elicited by natural infections(Heinen et al., 2021, White et al., 2021, Patel et al., 2017). In this study, we evaluated antibody responses to natural DENV infections. Indeed, a short time interval since the last natural infection has been shown to be associated with temporary cross-protection and protection from symptomatic dengue (Anderson et al., 2014; Sabin, 1952). Accordingly, a higher level of population seropositivity is associated with longer interepidemic periods and lesser force of infection, showing protective effect of seropositivity by natural immunity on a population-level (Aogo et al., 2023; Rodríguez-Barraquer et al., 2014). To address the Reviewer's comment and avoid confusion about protection, the statement has been revised (Line):

> *"In natural DENV infections, higher population seropositivity is associated with longer interepidemic periods and lower force of infection (Aogo et al., 2023). Consequently, we evaluated the seropositivity of each DENV-binding antibody subset at 18M after 1° versus 2° infections."*

"variability in antibody responses shape protection"
- Again, this concept requires increased clarity. What is population level protection? Are the authors proposing herd immunity to a specific DENV type or types due to high levels of type specific and cross reactive immunity in a population? A short description and references would strengthen the manuscript.

A: We thank the Reviewer for the opportunity to clarify this point. This Results statement is intended to set the contextual framework. The rationale is that individuals differ substantially from one another in the magnitude and kinetics of immune responses; a phenomenon that is being coalesced into an emerging field on immune set-points and host-specific intrinsic factors (Reviewed in Lei and Tsang, 2025). Some antibody and cellular immune responses vary more than others. The purpose of quantifying inter- and intra-individual variability is not to directly assert a link to protection, but rather to establish the essential biological reference range of the DENV-specific antibody response by subset. High interindividual variability in the waning kinetics of certain antibody subsets may lead to a portion of a population becoming seronegative for that immune response. Future mathematical models and epidemiological predictions of herd immunity may incorporate specific DENV antibody subsets and rely on precise variability estimates provided here for more accurate predictions. Thus, to address the Reviewer's comment, the opening and closing statements of the paragraph have been revised as follows (Lines and Lines 276-77):

> *"Given that inter- and intra-individual differences in immune responses are indicative of host-specific immune set-points (Lei and Tsang, 2025), we sought to quantify this heterogeneity by antibody subsets…. Thus, we identified that DENV4 XR E-binding IgG2 and IgG4 levels and kinetics are a highly variable antibody subset across individuals."*

Discussion

"assumption that cross-reactive antibody levels"
- Is it more accurate to state ALL cross reactive… There is plenty of antibody data measured using other assay platforms (classic PRNT or HAI) which clearly demonstrate waning over time after infection.

A: We agree and have revised the statement as per the Reviewer's suggestion (Line 301-303):

> *"First, it challenges the traditional assumption that all cross-reactive antibody levels wane after primary infection."*

"documented following 1° ZIKV infection"
- In dengue immune individuals? If so, I would state the same.

A: We thank the Reviewer for this opportunity to strengthen the point, and we have revised this statement (lines):

> *"Prior studies found that neutralizing antibody titers against a heterotypic DENV serotype continued to rise 2-3 years after 1° DENV infection in a pediatric hospital study and up to 7 years in a community-based cohort. (Katzelnick et al., 2021; Katzelnick et al., 2016)."*

"or in vaccinees"
- Differences would be expected I would imagine considering the simultaneous exposure to 4 DENV types.

A: We agree and removed "or in vaccinees" from this statement.

"facilitate DENV ADE"
- Again, the authors should be more precise in the language they use (ADE, enhancement, etc.). The accessibility of the manuscript to the non-dengue expert clinician or scientist will improve if terminology and descriptions of concepts are clearer.

A: This statement was removed as per the above response to the Reviewer.

Methods:

"Blood samples are collected at the time of hospital presentation "
- The study methods focus on collection of samples and characterization of immune responses from people who are infected, ill, and ill enough to present to the hospital for care. This would seem to eliminate the largest proportion of both primary and secondary infections (asymptomatic or subclinical) making the generalization of these data highly problematic and calls into question the mechanistic relevance. Please explain why you disagree.

A: We agree with the Reviewer's point that the exclusion of asymptomatic or subclinical cases limits generalizability of the findings, and we have included this as a limitation in the Discussion (Lines):

> *"This study included hospital pediatric dengue cases, which may differ from the antibody dynamics of community infections with asymptomatic or mild disease."*

Our reasoning for this design was two-fold:

- Molecular confirmation of DENV infection: We focused exclusively on PCR-confirmed DENV infections. In the absence of frequent and intensive household PCR testing, DENV infections must be inferred serologically for asymptomatic/subclinical cases (as boosts in antibody titer). We opted not to infer DENV infection by serology in order to have the highest confidence in the DENV infection status. This approach was necessary for this exploratory and comprehensive profiling of many DENV-specific antibody subsets, and it enabled reliable attribution of the kinetic to a confirmed DENV exposure.
- Kinetic Resolution: The primary goal was to model the detailed decay rates across the early, intermediate, and lasting antibody phases. Achieving this requires frequent, high-resolution sampling (early convalescent, 3-month, 6-month, and 18-month). Our cohort infrastructure used to capture inapparent infections relies on sparser, annual sampling, which would not provide the necessary temporal resolution for the antibody kinetics presented here.

We recognize the gap in knowledge regarding antibody dynamics of asymptomatic/subclinical cases and have recently currently conducted parallel studies to evaluate DENV-antibody dynamics in milder community

infections over 4 years, which allows delineation of differences in kinetics between symptomatic and subclinical individuals.

"longitudinal follow-up of convalescent samples at 3, 6, 12, 18…"
- How would flavivirus exposures which were subclinical be captured during the prolonged convalescent period? Would undetected homotypic or heterotypic or non-dengue flavi exposure impact outputs and if so how?

A: We thank the Reviewer for highlighting this important point. Subclinical infections are typically captured with a boosting of the immune response from one timepoint to another. Exposure to flaviviruses may occur in the 6-18-month period and here is most relevant to the key finding of rising cross-reactive IgG titers (DENV4 XR) observed in ~75% of the post-primary group. While there is potential for seasonal boosting in our 6-18M sampling range, multi-epidemic mathematical models based on our Nicaraguan study data suggest that 12-14% of a DENV-immune group may experience boosting annually (Aogo et al., 2023). This study sample set is not designed to accurately define a phenomenon that occurs in a small fraction of the population. Moreover, as noted before, only symptomatic cases were analyzed here, which are only a fraction of true DENV infections. Thus, our statements on boosting can only be speculative in this study. The main goal was to understand the distinct DENV-binding antibody subsets present and estimate their unique kinetics.

In the Discussion section, we previously had mentioned this potential limitation in line :

> *"Thus, boosting due to subclinical re-exposures likely contributes to the antibody kinetics observed here. Antibody dynamics following a single primary exposure in travelers or in vaccinees from non-endemic regions may follow different trajectories."*

However, we now additionally clarify this point in the Discussion:

Lines 468-73:

> *"Importantly, although occasional boosting from unrecognized DENV exposures may contribute to stable or rising antibody levels in some subsets, the distinct long-term waning or rising patterns observed across specific antibody subsets suggest that boosting alone is unlikely to fully account for our main findings. However, boosting due to subclinical re-exposures cannot be ruled out."*

- The premise of the manuscript is that these antibody profiles provide new insight into mechanisms of severe disease, but cohort and vaccine studies demonstrate the period of risk for severe disease is after 18 months have passed from a first infection. In this study the last collection is at 18 months. Do the authors think additional timepoints would yield more informative results as it relates to understanding what happens with clinical outcomes following second, sequential, heterotypic infections?

A: We agree with the Reviewer's point that the timeframe of risk for severe dengue is after 18 months. Consequently, parallel studies have evaluated antibody dynamics up to 4 years after infection. The reasoning for selection of samples up to 18 months post-symptom onset was because our group has previously shown from the same hospital study that the lasting immune dynamic of DENV-binding antibodies measured via DENV iELISA was reached at 8 months post-symptom onset and remained stable for years thereafter (Katzelnick et al., 2021). Thus, the final timepoint of 18 months was selected to surpass the 8-month transition to stable antibody kinetics. However, it is certainly possible that specific subsets of DENV-binding antibodies may continue to change after this timepoint, and well into the period of risk for subsequent infection.

Moreover, since groups of individuals differing by clinical outcome were not included in this study, statements on risk or protection can only be speculative and thus we prefer not to overstate a mechanism of

immunopathology. The key points of this study are that there exists heterogeneity in the antibody response by antigen and isotype/subclass, and post-primary cross-reactive DENV E-binding IgG antibodies rise significantly between 6-18M.

To address the comment, thesse following statements have been added to the Discussion of limitations (line 461-66):

> *"Of note, our longitudinal sampling timepoint is up to 18 months post-infection, and longer-term dynamics on the scale of 3-4 years after dengue will be needed to evaluate antibody dynamics in the typical risk period for subsequent disease; such studies from our Nicaraguan cohort are being reported separately. This study did not include participant groups with differences in infection or disease outcome; thus, the relationship between antibody kinetics and clinical risk or protection must be further investigated".*

"DENV infection is classified by an iELISA titer below 20"
- How primary and secondary infections are defined is extremely important. Is the ref 46 the correct reference to define how these cutoffs were derived?

A: We thank the Reviewer for indicating this discrepancy. The reference has been corrected in the Methods**:** Gutiérrez G, Gresh L, Pérez MÁ, Elizondo D, Avilés W, Kuan G, Balmaseda A, Harris E. Evaluation of the diagnostic utility of the traditional and revised WHO dengue case definitions. PLoS Negl Trop Dis. 2013 Aug 22;7(8):e2385. doi: 10.1371/journal.pntd.0002385. PMID: 23991237; PMCID: PMC3749970.

As previously mentioned, primary and secondary dengue were classified based on the DENV iELISA titer at acute and early convalescence, as follows in the methods section:

> *"A primary DENV infection is classified by an iELISA titer below 2560 in convalescent-phase samples; conversely, secondary infections are indicated by titers of 2560 or higher in convalescent-phase samples."*

This approach has been utilized in several prior published studies from our group (Katzelnick et al., 2017, 2021, etc).

- Are the authors using secondary infections to mean 2nd sequential infection with different dengue virus type or simply 2 or more infections? If the former, how is it confirmed a second infection was captured?

A: The term secondary refers to two or more infections, and this has been added to the methods for clarity (line 507): *"The term 2° refers to two or more DENV infections."*

Supp Table 1
- How was DHF defined? WHO 1997 criteria?

A: Correct, DHF was defined as per the WHO 1997 criteria, and this has been included in the methods in line 507:

> "Dengue hemorrhagic fever is defined as per the WHO 1997 criteria."

Supple Figure 1
- Nice schematic, may belong in main manuscript.

A: We thank the Reviewer for this suggestion. Rather than moving this schematic into the main figures, we chose to explicitly state its content in the main Results text. We added a new paragraph at the beginning of the Results section that defines antibody compartments and outlines the analytical framework used throughout the study (Lines 91-105), with direct reference to Supplementary Fig. S1.

> *"To interrogate the composition and kinetics of anti-DENV antibody responses, we leveraged antigen selection to distinguish antibody compartments (Supplementary Fig S1). For 1° dengue cases, antibodies measured*

against viral proteins from the infecting serotype (homologous antigens) represent a composite response that includes both type-specific antibodies unique to that serotype and cross-reactive antibodies targeting epitopes conserved across dengue viruses. In contrast, antibodies measured against proteins from non-infecting serotypes (heterologous antigens) must arise exclusively from cross-reactive antibodies, as individuals had no prior exposure to those serotypes. Additionally, for assessment of cross-reactive antibodies in both 1°and 2° cases, we considered that DENV4 did not circulate at epidemiologically relevant levels in Nicaragua from the early 1990s through the end of the study period (2013) and was not detected in 1° symptomatic infections. Therefore, DENV4 antigens were reliably used as the heterologous antigen to isolate cross-reactive antibody responses for both 1° and 2° dengue cases. We therefore use homologous antigens to measure the total (type-specific plus cross-reactive) antibody response, and heterologous DENV4 antigens to specifically capture the dynamics of cross-reactive antibodies."

REVIEWER COMMENTS

Reviewer #1 (Remarks to the Author):

I am satisfied that the authors have addressed all my comments. The paper's statistical methods have been strengthened, especially regarding the choice of models and the calculation of significance. However, the code provided is still insufficient to reproduce the main findings.

Reviewer #1 (Remarks on code availability):

The authors have provided the main R file containing the methods (Models.R). However, I was unable to run the code as the main data input was not provided. If the authors cannot provide the data, they should provide simulated data instead. Additionally, there is insufficient documentation to reproduce the model code, including the absence of a README file and limited guidance within the code.

A: We thank the Reviewer for this comment. Because the original dataset contains identifiable clinical data, it cannot be publicly shared. To improve reproducibility, we have now included a simulated dataset with the same structure as the original data, along with a README file and additional documentation to facilitate running the code: available at https://zenodo.org/records/19155076 (doi: 10.5281/zenodo.19155076).

Reviewer #2 (Remarks to the Author):

In this revised manuscript, the authors have substantially clarified the basis for their inferences and have supplemented their initial analysis based on linear mixed models with alternative analyses including a more direct non-parametric comparison. The authors have also made substantial efforts to address criticisms and questions regarding the framing of their conclusions.
As noted in the previous review, this dataset has high overall significance. Therefore, the comments below focus on remaining issues in the presentation and interpretation of the data.

Major comments:

1. **The authors clarify that they define "significant" differences when the 95% CI of the comparison group did not overlap with the corresponding point estimate for the reference group (or zero in the case of defining rising or waning). While they removed textual references to "statistically significant" and no longer cite p values, the use of asterisks in figures implies statistical significance that is not supported by this analysis. This will be misleading to most readers and should be deleted.**

A: We respectfully disagree that the asterisks require removal. The Reviewer's concern appears to rest on an implicit equivalence between "statistical significance" and "p-value-based significance," but these are not synonymous. Statistical significance means the observed result is unlikely under a null hypothesis; a p-value is one way to operationalize that inference, but not the definition of it. Established alternatives include Bayesian credible intervals (significance declared when the interval excludes the null), permutation-based approaches (significance established through resampling without classical p-values), and CI-overlap criteria. The last is the approach we employed. A difference flagged by non-overlap of a 95% CI is, by definition, statistically meaningful under that criterion: if the 95% bootstrap confidence interval for the difference excludes zero, this indicates evidence against the null hypothesis of no difference. We therefore believe that the symbol is both accurate and appropriate. Please note that each figure legend defines the meaning of an asterisk precisely and unambiguously

without implying p-value-based hypothesis testing. We thus believe that the asterisk serves its intended communicative purpose. For additional clarity, we have moved the statement on the definition of the asterisks in each Figure Legend for figures 1-3 to the final statement of the figure legend, so that it is clearly visible.

2. **As pointed out in the prior review, primary versus secondary infections were defined based on the antibody response to infection, specifically, the antibody response measured at the convalescent time point. Whether iELISA or multiplex assay is used to make this determination is irrelevant to the potential implications, particularly for analyses of antibody trajectories between early convalescence and 3 months. The potential misclassification of cases is an important limitation of their analysis that needs to be acknowledged in the Discussion.**

A: We thank the Reviewer for this comment. Defining primary versus secondary infections serologically is based on our biological understanding of how primary versus secondary immune responses work, in that secondary immunity arises faster and attains a higher level than primary immunity. This knowledge has been used to identify primary versus secondary dengue cases using the profile of high early convalescent antibody response in secondary relative to primary cases and is a widely used and conventional approach in dengue immunology studies. A few examples include:

Gordon A, Kuan G, Mercado JC, Gresh L, Avilés W, Balmaseda A, Harris E. The Nicaraguan pediatric dengue cohort study: incidence of inapparent and symptomatic dengue virus infections, 2004-2010. PLoS Negl Trop Dis. 2013 Sep 26;7(9):e2462. doi: 10.1371/journal.pntd.0002462. PMID: 24086788; PMCID: PMC3784501

Patel B, Longo P, Miley MJ, Montoya M, Harris E, de Silva AM. Dissecting the human serum antibody response to secondary dengue virus infections. PLoS Negl Trop Dis. 2017 May 15;11(5):e0005554. doi: 10.1371/journal.pntd.0005554. PMID: 28505154; PMCID: PMC5444852.

Innis BL, Nisalak A, Nimmannitya S, Kusalerdchariya S, Chongswasdi V, Suntayakorn S, Puttisri P, Hoke CH. An enzyme-linked immunosorbent assay to characterize dengue infections where dengue and Japanese encephalitis co-circulate. Am J Trop Med Hyg. 1989 Apr;40(4):418-27. doi: 10.4269/ajtmh.1989.40.418. PMID: 2540664.

Waickman AT, Gromowski GD, Rutvisuttinunt W, Li T, Siegfried H, Victor K, Kuklis C, Gomootsukavadee M, McCracken MK, Gabriel B, Mathew A, Grinyo I Escuer A, Fouch ME, Liang J, Fernandez S, Davidson E, Doranz BJ, Srikiatkhachorn A, Endy T, Thomas SJ, Ellison D, Rothman AL, Jarman RG, Currier JR, Friberg H. Transcriptional and clonal characterization of B cell plasmablast diversity following primary and secondary natural DENV infection. EBioMedicine. 2020 Apr;54:102733. doi: 10.1016/j.ebiom.2020.102733. Epub 2020 Apr 18. PMID: 32315970; PMCID: PMC7170960.

Rouers A, Chng MHY, Lee B, Rajapakse MP, Kaur K, Toh YX, Sathiakumar D, Loy T, Thein TL, Lim VWX, Singhal A, Yeo TW, Leo YS, Vora KA, Casimiro D, Lim B, Tucker-Kellogg L, Rivino L, Newell EW, Fink K. Immune cell phenotypes associated with disease severity and long-term neutralizing antibody titers after natural dengue virus infection. Cell Rep Med. 2021 May 18;2(5):100278. doi: 10.1016/j.xcrm.2021.100278. PMID: 34095880; PMCID: PMC8149372.

However, we agree that some degree of misclassification is possible when the infection group is inferred from post-infection antibody responses. To address this concern, we have now explicitly acknowledged this potential limitation in the Discussion.

Lines 455-457: "Primary versus secondary immune status was inferred serologically as per widely applied and conventional standards in the field, though some level of misclassification is possible."

3. **In numerous places in the manuscript, the authors draw conclusions regarding the timing of peak responses during the C-3M window (e.g., "delayed peak", "delayed initiation"). Since antibody responses were only measured at the convalescent and 3M time points, peak responses likely were not captured. These statements should be revised accordingly.**

A: We acknowledge that peak responses may occur between the convalescent and 3-month time points and therefore cannot be directly observed. To improve clarity, we have revised the text to avoid language implying direct measurement of peak timing and instead refer to relative differences in antibody levels between these two sampling points.

Lines 123: "In contrast, the initial magnitude of anti-E IgA, IgM, and IgG3 responses were higher post-1° than post-2° infection, ~~peaking~~ reaching maximal observed levels at or before convalescence (Fig 1A)."

Lines 145: "Many aspects were comparable, such as higher levels of NS1-IgG in early 2° compared to 1° responses, ~~a delayed peak~~ a more gradual rise toward maximal recorded levels of NS1-IgG in 1° compared to 2° responses, and a similar directionality of NS1-IgA and NS1-IgM in both 1° and 2° responses (Fig 2A)."

Lines 149-150: "In 2° infections, NS1-IgG and its IgG1 and IgG2 subclasses ~~demonstrated a sustained peak response~~ remained elevated at maximal observed levels through 3M, whereas anti-E IgG declined in this period (Fig 2A, D)."

Lines 361-362: "Consistent with earlier findings, our data confirm distinct antibody trajectories across 1° versus 2° DENV infections, notably ~~the earlier and higher peak~~ earlier attainment of maximal observed levels and higher magnitude of anti-E IgG, particularly IgG1 and IgG2, and the sustained higher levels of XR E-IgG (IgG1, IgG2, IgG4) at 18 months after 2° compared to 1° DENV infections."

4. **The authors provide a formula for the linear effects model using factors with 3 or more levels (lines 565-571). However, it is not clear that these models were actually used in the manuscript, as it appears that all of the curves presented in the manuscript were based on the linear models of primary versus secondary. If so, the Methods should be revised accordingly. If this is not the case, the authors should make clear which model was used to generate data presented in each figure.**

A: We thank the Reviewer for this comment. Figures 1–3 were generated using models evaluating primary versus secondary infections as the main effect. Figure 4 instead evaluates antigen as the primary factor in the model, and Figure 5 evaluates the antibody response type (i.e., infecting serotype, DENV2 XR, DENV4 XR, and ZIKV XR) as the main effect. We have clarified this distinction in the Methods (Lines 579-582) and figure descriptions.

Methods

"As a clarification, Figures 1–3 were generated using models evaluating primary versus secondary infections as the main effect (i.e., infection history group). Figure 4 instead evaluates antigen as the primary factor in the mode (i.e., E, EDIII, and NS1), and Figure 5 evaluates antibody response type (i.e., infecting serotype, DENV2 XR, DENV4 XR, and ZIKV XR) as the main effect."

Figure 1 legend

Figure 1. Antibody responses against homologous E protein after primary and secondary dengue. *E-binding antibodies against the homologous infecting serotype were measured as Log$_2$ of mean fluorescence intensity (MFI) after background subtraction (BG; shaded area). A linear mixed-effects model with bootstrapping was applied to estimate a linear rate of decay for three time-period segments: 30-90, >90-180, and >180-540 days post-symptom onset, corresponding to early convalescence to 3 months (Cv-3M), 3-6*

months (3-6M), and 6-18 months (6-18M) post-symptom onset. The model included an interaction term between the time segments and infection group (primary versus secondary) as the primary factor of interest. Results presented here are restricted to the homotypic response against the E protein and are stratified by antibody isotype and subclass. ***a.*** *Homologous E-binding antibody levels over time of primary (P; dotted line, yellow) versus secondary (S; solid line, grey) dengue cases with 95% confidence interval for each isotype and IgG subclass. Antibodies levels are represented as the Log_2 fold-change over background (Log_2[MFI] - Log_2 [Background]); BG: shaded area).* ***b, c.*** *Short-term antibody half-lives ($t_{1/2}$) estimated from Cv-3M antibody decay rates (**b**), and (**c**) long-term $t_{1/2}$ estimated from 6-18M antibody decay rates. Transparent colors indicate stable decay rates with non-estimable infinite (-Inf) $t_{1/2}$ (i.e., Not Sig., not significant). Solid colors are significantly rising (-$t_{1/2}$ above -Inf on y-axis) or waning (+$t_{1/2}$ below -Inf on y-axis; shaded area) antibody kinetics. For time segments in which the modeled antibody isotype/subtype trajectory remained at or below zero over the entire interval (a), the corresponding half-life estimates and CI were omitted from panels b and c to avoid misleading interpretation.* ***d.*** *Comparison of the levels of homologous E-binding antibodies by isotype and IgG subclass after primary and secondary dengue. Asterisks (*) denote significant differences between primary and secondary decay rates based on a confidence-interval overlap criterion: a group (S) was considered to differ from the reference group (P) when its 95% CI for the decay rate did not overlap the reference group's point estimate."*

Figure 2 legend

*"**Figure 2. Dynamics of the antibody response against homologous DENV NS1 protein up to 18 months post-symptom onset.** NS1-binding antibodies against the homologous infecting serotype (DENV1 or DENV3) were measured as Log_2 of mean fluorescence intensity (MFI) after background subtraction (BG; shaded area). A linear mixed-effects model with bootstrapping was applied to estimate a linear rate of decay for three time-period segments: 30-90, >90-180, and >180-540 days post-symptom onset, corresponding to convalescent to 3-month (Cv-3M), 3-6 months (3-6M), and 6-18 months (6-18M) post-symptom onset. The model included an interaction term between the time segments and infection group (primary versus secondary) as the primary factor of interest. Results presented here are restricted to the homologous response against the NS1 protein and are stratified by antibody isotype and subclass.* ***a.*** *Homologous DENV NS1-binding antibody levels over time of primary (P; dotted line, yellow) versus secondary (S; solid line, grey) dengue cases with 95% confidence interval for each isotype and IgG subclass. Antibodies levels are represented as the Log_2 fold-change over background (Log_2[MFI] - Log_2[Background]); BG: shaded area).* ***b, c.*** *Short-term antibody half-lives ($t_{1/2}$) estimated from Cv-3M antibody decay rates (**b**), and **(c)** long-term $t_{1/2}$ estimated from 6-18M antibody decay rates. Transparent colors indicate stable decay rates with non-estimable infinite (-Inf) $t_{1/2}$ (i.e., Not Sig., not significant). Solid colors are significantly rising (-$t_{1/2}$ above -Inf on y-axis) or waning (+$t_{1/2}$ below -Inf on y-axis; shaded area) antibody kinetics. Asterisks denote significant differences between primary and secondary decay rates based on a confidence-interval overlap criterion: a group (S) was considered to differ from the reference group (P) when its 95% CI for the decay rate did not overlap the reference group's point estimate. For time segments in which the modeled antibody isotype/subtype trajectory remained at or below zero over the entire interval (a), the corresponding half-life estimates and CI were omitted from panels b and c to avoid misleading interpretation.* ***d.*** *Comparison of the levels of homologous NS1-binding antibodies by isotype and IgG subclass after primary and secondary dengue. Asterisks (*) denote significant differences between primary and secondary decay rates based on a confidence-interval overlap criterion: a group (S) was considered to differ from the reference group (P) when its 95% CI for the decay rate did not overlap the reference group's point estimate."*

Figure 3 legend

"***Figure 3. DENV serocomplex cross-reactive anti-E antibodies rise after primary dengue but are stable post-secondary dengue.*** *Cross-reactive (XR) E-binding antibodies were assessed as Log_2 of mean*

fluorescence intensity (MFI) after background subtraction (BG; shaded area), against the DENV4 serotype, which was infrequent in our cohort during the time-period of infections sampled here. A linear mixed-effects model with bootstrapping was applied to estimate a linear rate of decay for three time-period segments: 30-90, >90-180, and >180-540 days post-symptom onset, corresponding to convalescent to 3-month (Cv-3M), 3-6 months (3-6M), and 6-18 months (6-18M) post-symptom onset. The model included an interaction term between the time segments and infection group (primary versus secondary) as the primary factor of interest. Results presented here are restricted to the DENV4 response against the NS1 protein and are stratified by antibody isotype and subclass. ***a.*** *XR E-binding antibody levels over time of primary (P; dotted line, blue) versus secondary (S; solid line, grey) dengue cases with 95% confidence interval for each isotype and IgG subclass. Antibodies levels are represented as the Log_2 fold-change over background (Log_2[MFI] - Log_2[Background]); BG: shaded area).* ***b, c.*** *Short-term antibody half-lives ($t_{1/2}$) estimated from Cv-3M antibody decay rates (**b**), and (**c**) long-term $t_{1/2}$ estimated from 6-18M antibody decay rates. Transparent colors indicate stable decay rates with non-estimable infinite (-Inf) $t_{1/2}$ (i.e., Not Sig., not significant). Solid colors are significantly rising (-$t_{1/2}$ above -Inf on y-axis) or waning (+$t_{1/2}$ below -Inf on y-axis; shaded area) antibody kinetics. Asterisks denote significant differences between primary and secondary decay rates based on a confidence-interval overlap criterion: a group (S) was considered to differ from the reference group (P) when its 95% CI for the decay rate did not overlap the reference group's point estimate. For time segments in which the modeled antibody isotype/subtype trajectory remained at or below zero over the entire interval (a), the corresponding half-life estimates and CI were omitted from panels b and c to avoid misleading interpretation. Asterisks (*) denote significant differences between primary and secondary decay rates based on a confidence-interval overlap criterion: a group (S) was considered to differ from the reference group (P) when its 95% CI for the decay rate did not overlap the reference group's point estimate."*

Figure 4 legend

*"**Figure 4. Distinct dynamics of antibodies against E, EDIII, and NS1 proteins.** DENV-binding IgG levels were measured as Log_2 of mean fluorescence intensity (MFI) after background subtraction (BG; shaded area). A linear mixed-effects model with bootstrapping was applied to estimate a linear rate of decay for three time-period segments: 30-90, >90-180, and >180-540 days post-symptom onset, corresponding to convalescent to 3-month (Cv-3M), 3-6 months (3-6M), and 6-18 months (6-18M) post-symptom onset. The model included an interaction term between the time segments and antigen as the primary factor of interest. Results presented here are restricted to the DENV4 response against the NS1 protein and are stratified by antibody isotype and subclass. **a, d**. Comparison of homologous (**a**) and DENV4 (**d**) E (blue), EDIII (lime green), and NS1 (pink) binding IgG levels over time in the primary (P; dotted line) versus secondary (S; solid line) dengue groups. Antibodies levels are represented as the Log_2 fold-change over background (Log_2[MFI] - Log_2[Background]); BG: shaded area). **b, c**. Fold-change in binding IgG levels against E, EDIII, and NS1 from the infecting serotype between Cv-3M (**b**) and 6-18M (**c**). **e, f.** Fold-change in binding IgG levels against DENV4 E, EDIII, and NS1 between Cv-3M (**e**) and 6-18M (**f**). For each infection group, fold-change in EDIII and NS1 responses was compared with fold-change in the corresponding E response using paired, Bonferroni-corrected Wilcoxon tests. Asterisks denote significant differences derived from adjusted p-values (*$p < 0.05$; **$p < 0.01$; ***$p < 0.001$). When the modeled antigen response trajectory in panel a remained at or below zero over the entire time segment, the corresponding fold-change values were omitted from panels b, c, e and f to avoid misleading interpretation."*

Figure 5 legend

"Figure 5. Antigenic hierarchy in the levels and kinetics of cross-reactive antibody responses post-primary dengue. *The magnitude of flavivirus E-, EDIII-, and NS1-binding IgG was measured as Log_2 of mean fluorescence intensity (MFI). A linear mixed-effects model with bootstrapping was applied to estimate a linear rate of decay for three time-period segments: 30-90, >90-180, and >180-540 days post-symptom onset,*

corresponding to convalescent to 3-month (Cv-3M), 3-6 months (3-6M), and 6-18 months (6-18M) post-symptom onset. The model included an interaction term between the time segments and antibody response type as the primary factor of interest. ***a.*** *In the primary dengue group (dotted line), total DENV-reactive IgG against the infecting serotype of DENV1 or DENV3 (yellow) were compared to the magnitude of DENV2 serotype cross-reactive (DENV2 XR; green), DENV4 serotype cross-reactive (DENV4 XR; blue), and ZIKV cross-reactive (ZIKV XR; grey) antibodies over time. Antibodies levels are represented as the Log_2 fold-change over background (Log_2[MFI] - Log_2[Background]); BG: shaded area).* ***b, c.*** *Fold-change in binding IgG levels observed for each antibody response between Cv-3M (**b**) and 6-18M (**c**). For each antigen, fold-change in cross-reactive responses was compared to the infecting-serotype response using paired, Bonferroni-corrected Wilcoxon tests. Asterisks denote significant differences derived from adjusted p-values (*$p < 0.05$; **$p < 0.01$; ***$p < 0.001$). When the modeled antibody response trajectory in panel a remained at or below zero over the entire time segment, the corresponding fold-change values were omitted from panels b and c to avoid misleading interpretation."*

5. **In the prior review, it was suggested that the authors comment on whether data from the other serum dilutions supported their most novel findings. The authors' response justifies use of the 1:800 dilution for the quantitative analyses presented in the manuscript. However, it would be expected that other dilutions would fall within the interpretable range for specific comparisons of interest that they consider to provide "novel conceptual insights". The reporting of such supportive information (or lack thereof) would be very helpful to the scientific community given that the data already exist.**

A: We appreciate this suggestion and agree that evaluating whether our findings are consistent across serum dilutions strengthens the interpretability and generalizability of our results. To address this, we performed a systematic sensitivity analysis across all available dilutions (1/200 to 1/12,400), applying the same mixed-effects spline modeling framework used in the primary analyses.

We assessed the consistency of the signal by comparing model performance using conditional R squares across dilutions while holding the model structure constant (adjusting for age and sex, with a single interaction term for infection group) and using a frequentist linear mixed model approach. For each model, the infection group was evaluated while stratifying by antigen, antibody response type, and antibody isotype/subclass. Across all stratifications, we observed that model performance was highly consistent over a broad intermediate range of dilutions. Conditional R squared values plateaued between 1/200 and 1/3200, with overlapping interquartile ranges across strata (Table R1).

Importantly, while the 1/800 dilution used in the primary analyses achieved the maximal explanatory power across comparison groups, the interaction effect sizes and statistical significance remained remarkably stable across the entire dilution range (see figures below). Even at the highest dilution (1/12,400), where we observed a slight decline in R squared consistent with a reduced signal-to-noise ratio, the direction and magnitude of the immune response effect were preserved (Figures R1-R3). Overall, our results using the 1/800 dilution are robust.

Table R1. Sensitivity Analysis of Model Explanatory Power (Conditional R squares) Across Serum Dilutions.

Comparison	Dilution	Median R² [IQR]
IR	200	0.63 [0.56, 0.73]
IR	800	0.66 [0.60, 0.75]
IR	3200	0.63 [0.53, 0.75]
IR	12400	0.58 [0.48, 0.67]

Summary of 84 mixed-effects spline models evaluating the interaction between Immune Response (IR) and time segments, stratified by antibody isotype, antigen target, and viral response (adjusted for age and sex).

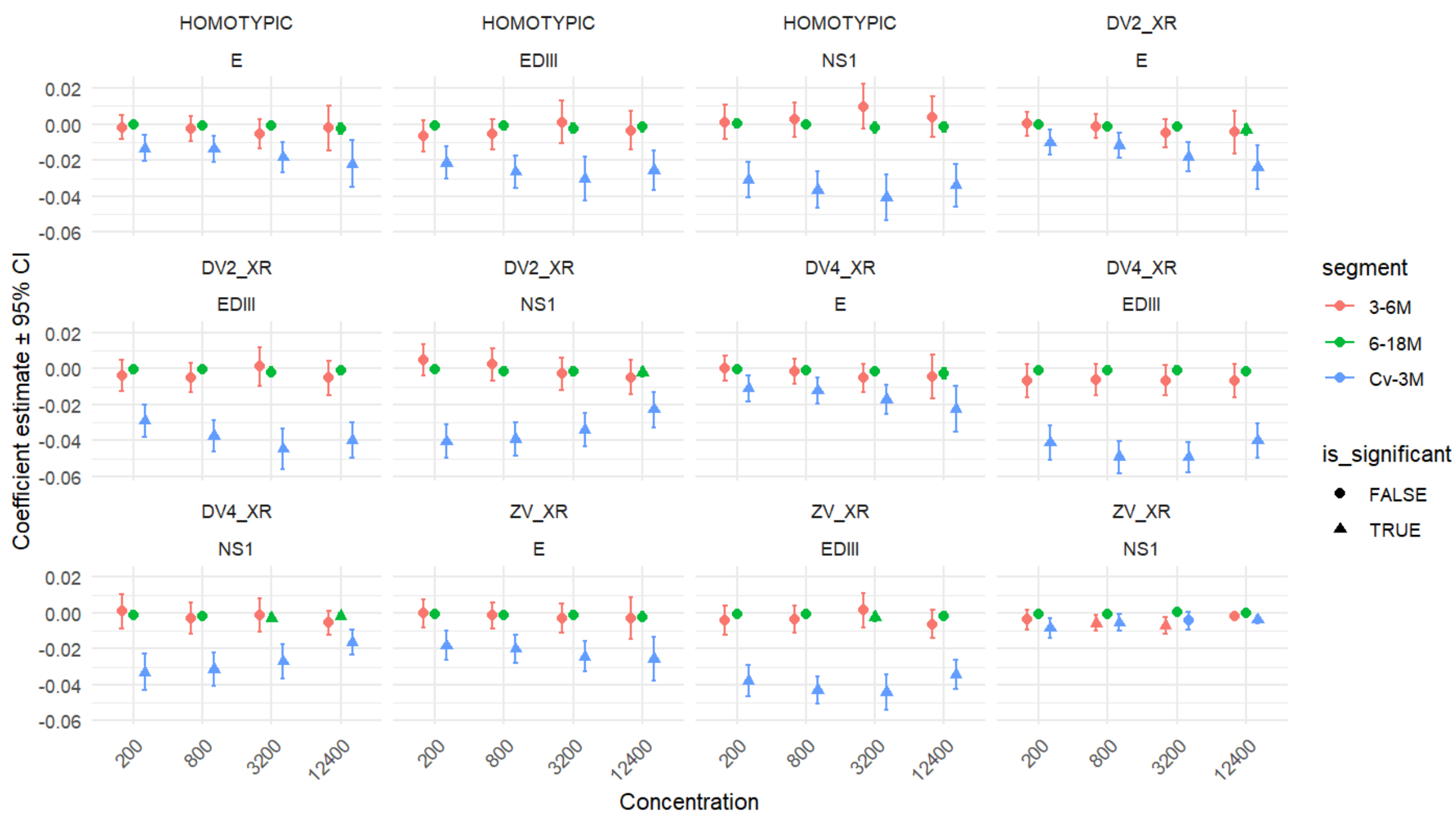

Figure R1. Sensitivity analysis of immune response-time interaction effects for IgG. Each panel represents a unique stratum of antibody response type and antigen target. Points denote the fixed-effect interaction coefficients between the immune response (IR) group and specific time segments (Cv-3M, 3-6M, and 6-18M) derived from a mixed-effects spline model. Models were adjusted for age and sex as fixed effects, with a random intercept included for individual subjects (Code). Error bars represent the 95% Confidence Interval (CI) calculated using the t-distribution. Statistical significance ($p < 0.05$) for the interaction term is indicated by triangular points (True) versus circular points (False).

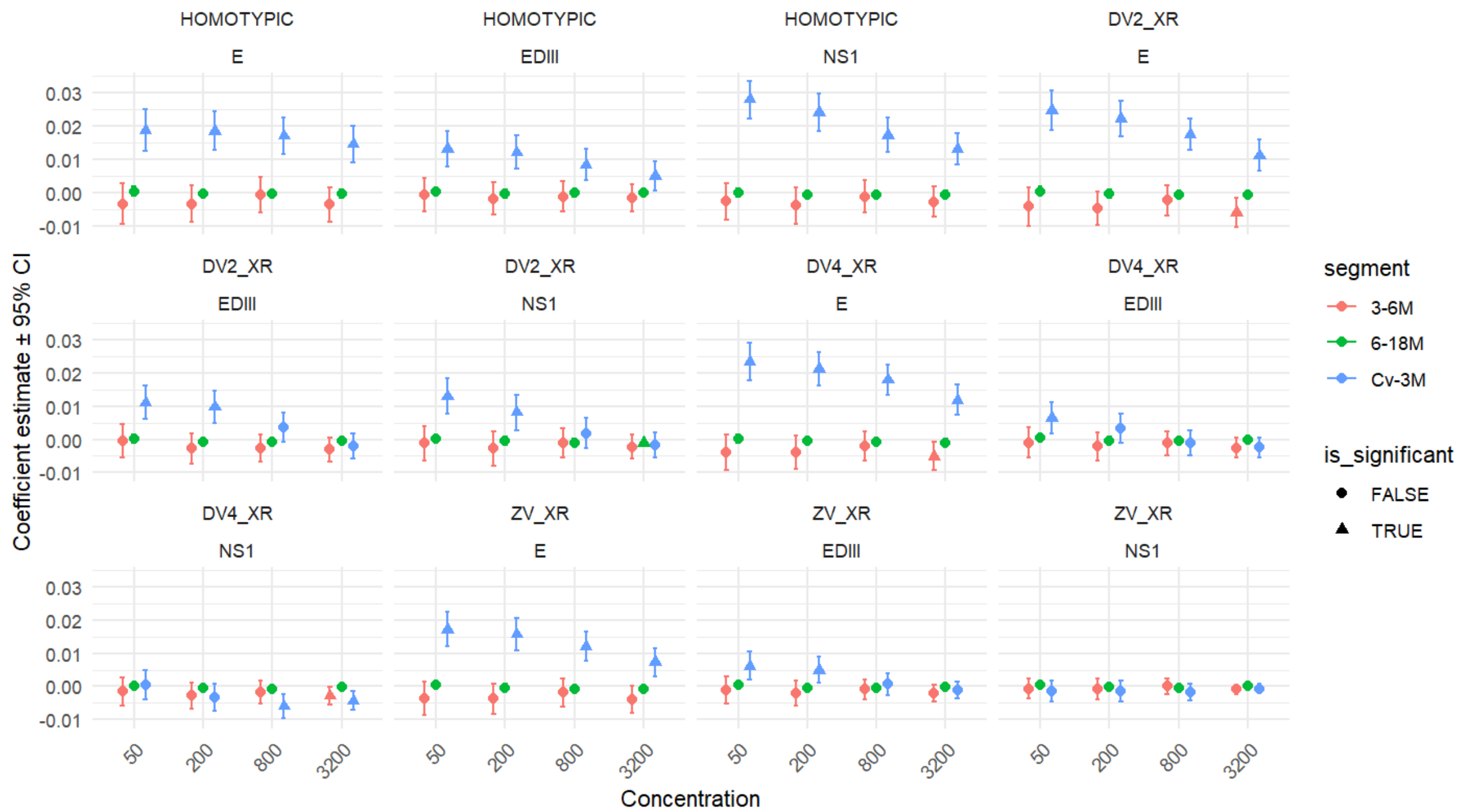

Figure R2. Sensitivity analysis of immune response-time interaction effects for IgA. Each panel represents a unique stratum of antibody response type and antigen target. Points denote the fixed-effect interaction coefficients between the immune response (IR) group and specific time segments (Cv-3M, 3-6M, and 6-18M) derived from a mixed-effects spline model. Models were adjusted for age and sex as fixed effects, with a random intercept included for individual subjects (Code). Error bars represent the 95% Confidence Interval (CI) calculated using the t-distribution. Statistical significance ($p < 0.05$) for the interaction term is indicated by triangular points (True) versus circular points (False).

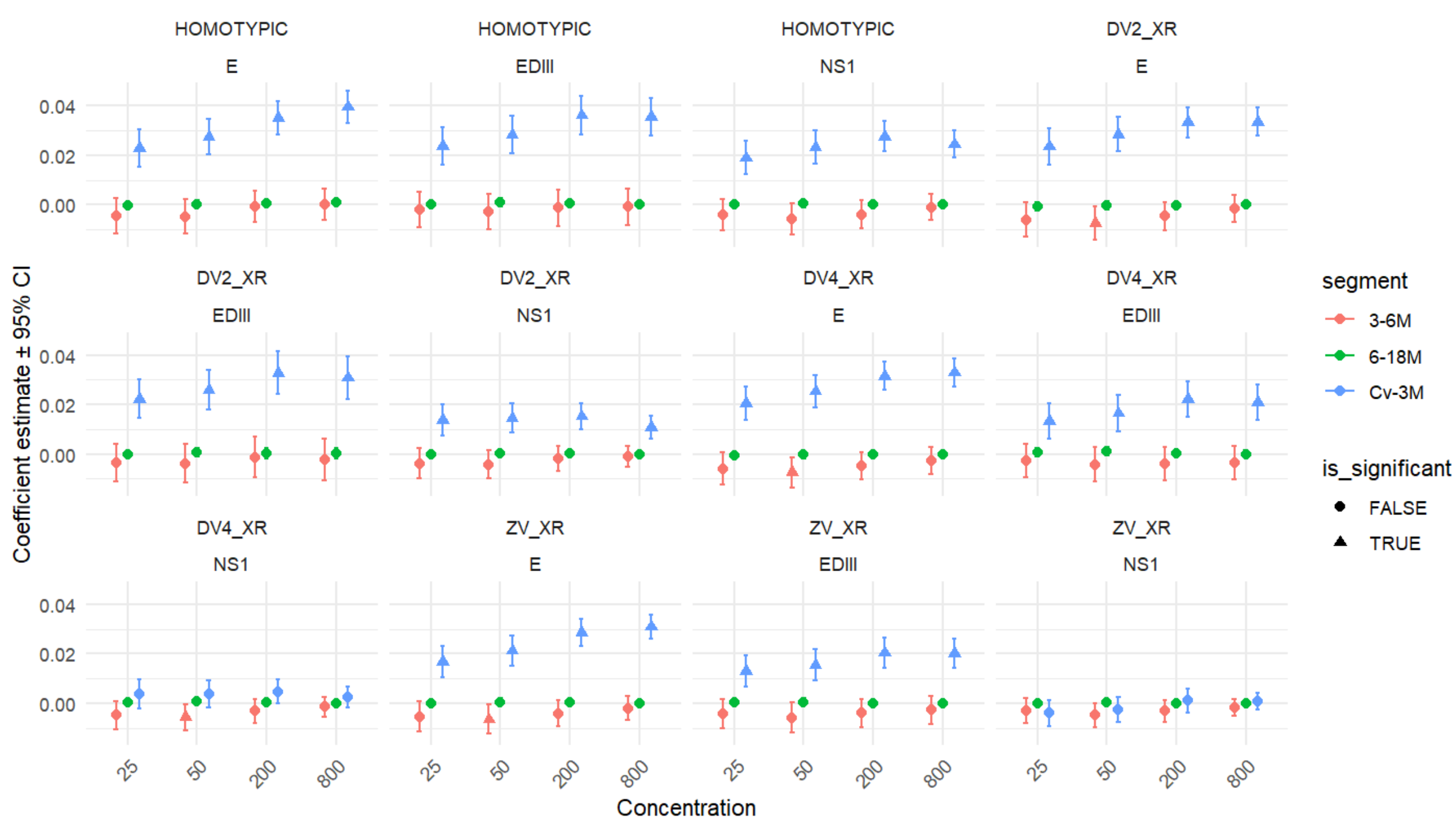

Figure R3. Sensitivity analysis of immune response-time Interaction effects for IgM. Each panel represents a unique stratum of antibody response type and antigen target. Points denote the fixed-effect interaction coefficients between the immune response (IR) group and specific time segments (Cv-3M, 3-6M, and 6-18M) derived from a mixed-effects spline model. Models were adjusted for age and sex as fixed effects, with a random intercept included for individual subjects (Code). Error bars represent the 95% Confidence Interval (CI) calculated using the t-distribution. Statistical significance ($p < 0.05$) for the interaction term is indicated by triangular points (True) versus circular points (False).

As noted above, our model performance was consistent. Nonetheless, we feel that publishing figures from suboptimal dilutions could be misleading, as we pointed out earlier:

> "At the lower dilutions (1:50 and 1:200), most samples from secondary DENV infections exhibited saturating MFI values, consistent with a prozone effect. At the highest dilution (1:3200), signal intensity was insufficient to consistently detect lower-abundance antibody isotypes and subclasses (notably IgM and IgG3), even at early convalescent time points (e.g., 3 months post-infection), when these responses are biologically expected to be detectable.
>
> As a result, the 1:800 dilution represented the only dilution that provided a robust and consistent dynamic range across both primary and secondary infection groups, all antibody isotypes/subclasses, and all analyzed timepoints. For this reason, all quantitative analyses presented in the manuscript were performed using data from the 1:800 dilution."

6. **Abstract- Statements of conclusions in the Abstract should be as accurate as possible. The following changes are recommended- "IgG antibodies against the envelope protein rise" (line 27) to "IgG antibody responses against the envelope protein rise" and "substantial seropositivity of IgA, IgM, and IgG3 at 18 months post-infection was observed" (line 29) to "a substantial fraction of subjects still had IgA, IgM, and IgG3 responses above the assay background at 18 months post-infection".**

A: We thank the Reviewer for this suggestion. We agree and have revised the Abstract accordingly.

Abstract

"The four dengue virus serotypes (DENV1-4) co-circulate worldwide, posing major challenges for vaccine development. One key issue is that certain levels and subsets of cross-reactive antibodies have been associated with enhanced disease during subsequent infection with a different DENV serotype. To understand the heterogeneity of DENV antibody responses and delineate their distinct kinetics, we defined the magnitude and kinetics of 84 antiviral antibody subsets (by isotype, subclass, antigen, and cross-reactivity) after primary versus secondary dengue, using longitudinal samples collected <1, 3, 6 and 18 months post-symptom onset from a pediatric hospital study in Nicaragua. Interestingly, we found that post-primary infection, cross-reactive IgG antibody responses against the envelope protein rise, not wane, over time. Antibody kinetics varied by specificity as measured by homologous versus cross-reactive subsets, viral antigen, and subdomain of a single antigen. Further, a substantial fraction of subjects still had IgA, IgM, and IgG3 responses above the assay background at 18 months post-infection. Thus, the cross-reactive subset of post-primary anti-DENV antibody responses demonstrate distinct kinetics from overall DENV-binding antibodies as well as from secondary immune responses, which have implications for the outcome of subsequent DENV infections."

Minor comments:

7. **Figure 1-5- "remained at or below infinite" in the legends should read "remained at or below zero".**

A: We thank the Reviewer for noting this error. We have corrected the text in the figure legends to read "remained at or below zero."

8. **Figures 4b/c, 5b/c, 7b- y axis values (all <0.05) do not appear to comport with the definition of fold change provided on lines 655-656. (Perhaps the authors divided by the number of days between samples?)**

A: We thank the Reviewer for this excellent observation. The values shown represent the change in antibody levels normalized by the time interval between samples (i.e., change per year). We have revised the Methods to clarify this definition and avoid confusion with a traditional fold change metric.

> *Lines 662-665: "As neither the bootstrap-based LMMs nor the BLMM explicitly estimate individual-level random slopes at each antibody compartment level, we performed Wilcoxon tests on individual-level data. For each participant, the annualized fold-change was defined as the difference in Log_2(MFI) between two timepoints divided by the time interval in years, within each time segment (Cv–3M and 6–18M). All tests were two-sided."*

9. **Figures S10, S11- The distribution of values on both x and y axes is inconsistent with the data presented in other figures. Correlation (r) values would be more informative than p values. Shared areas should be defined. The legends should be revised- maximum values were not WITHIN 3 months of infection according to the information in Table S1.**

A: We thank the Reviewer for these comments. We identified that results in Figures S10 and S11 had indeed not been background-subtracted, inconsistent with the presentation in other figures. We have corrected this and replotted both figures with background subtraction applied. Spearman rank correlation coefficients (ρ) and associated p-values are now displayed for each infection group in all panels. Shaded areas have been removed from these figures as all values were above background after correction. The figure and legends have been revised accordingly. We also clarify that the maximal observed antibody level was defined from samples collected up to 3 months post-symptom onset. Table S1 reports mean days post-symptom onset across individuals, which does not directly correspond to the timing of individual maximum values.

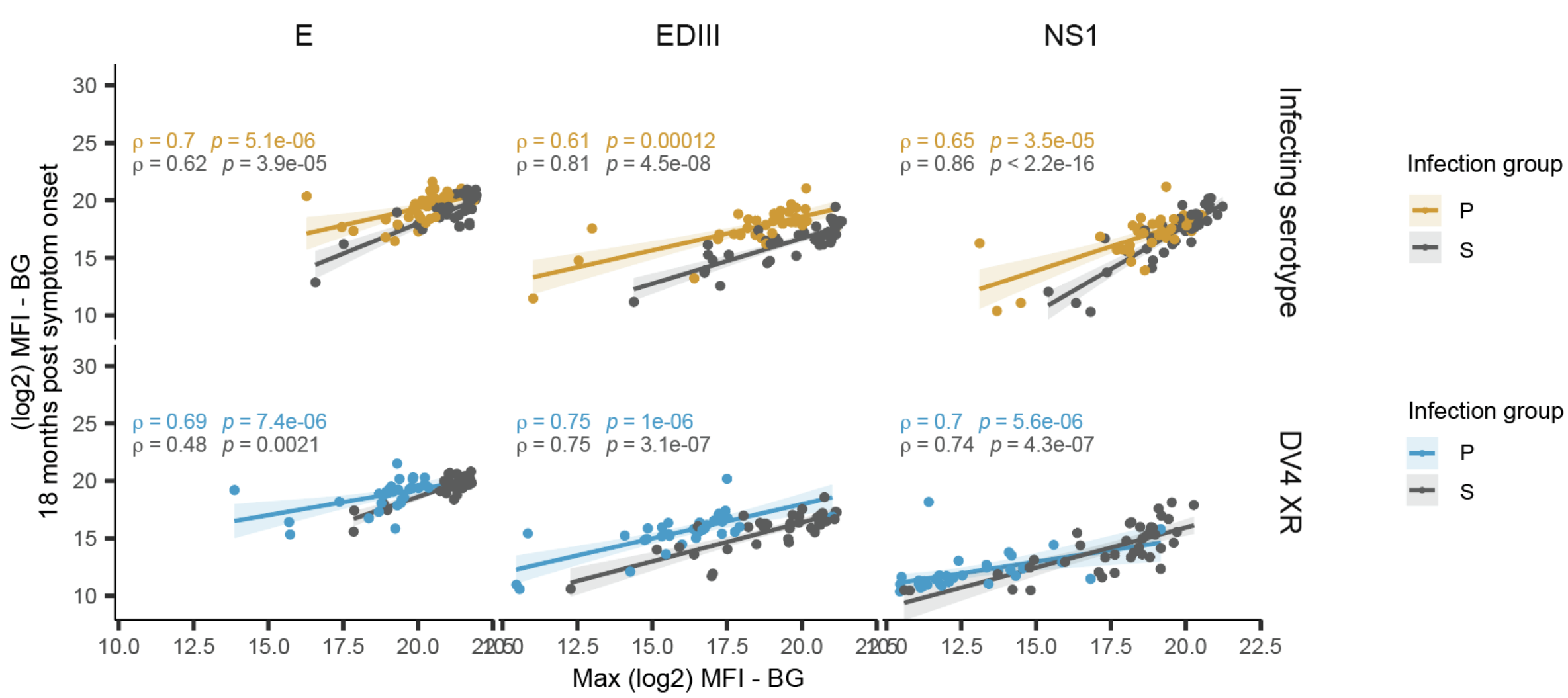

Supplementary Figure 10. Relationship between maximal observed early IgG1 antibody response and the level remaining at 18 months post-infection by antigen. *E-binding plasma IgG1 antibodies were measured as log_2 of mean fluorescence intensity (MFI) after background subtraction (BG). Post-primary*

responses against the infecting serotype are in yellow, and post-primary responses against cross-reactive DENV4 serotype are in blue (DV4 XR). All post-secondary antibody responses are in grey. The maximal observed level of DENV-binding IgG1 (i.e., Max) was defined for each individual from samples collected up to 3 months post-symptom onset. The magnitude of this maximal early DENV-binding IgG1 was correlated with the magnitude of the same antibody subset at 18 months post-symptom onset. Spearman ρ and associated p-values are shown for each infection group. Each dot represents an individual. Axes were scaled to data for each antibody subset.

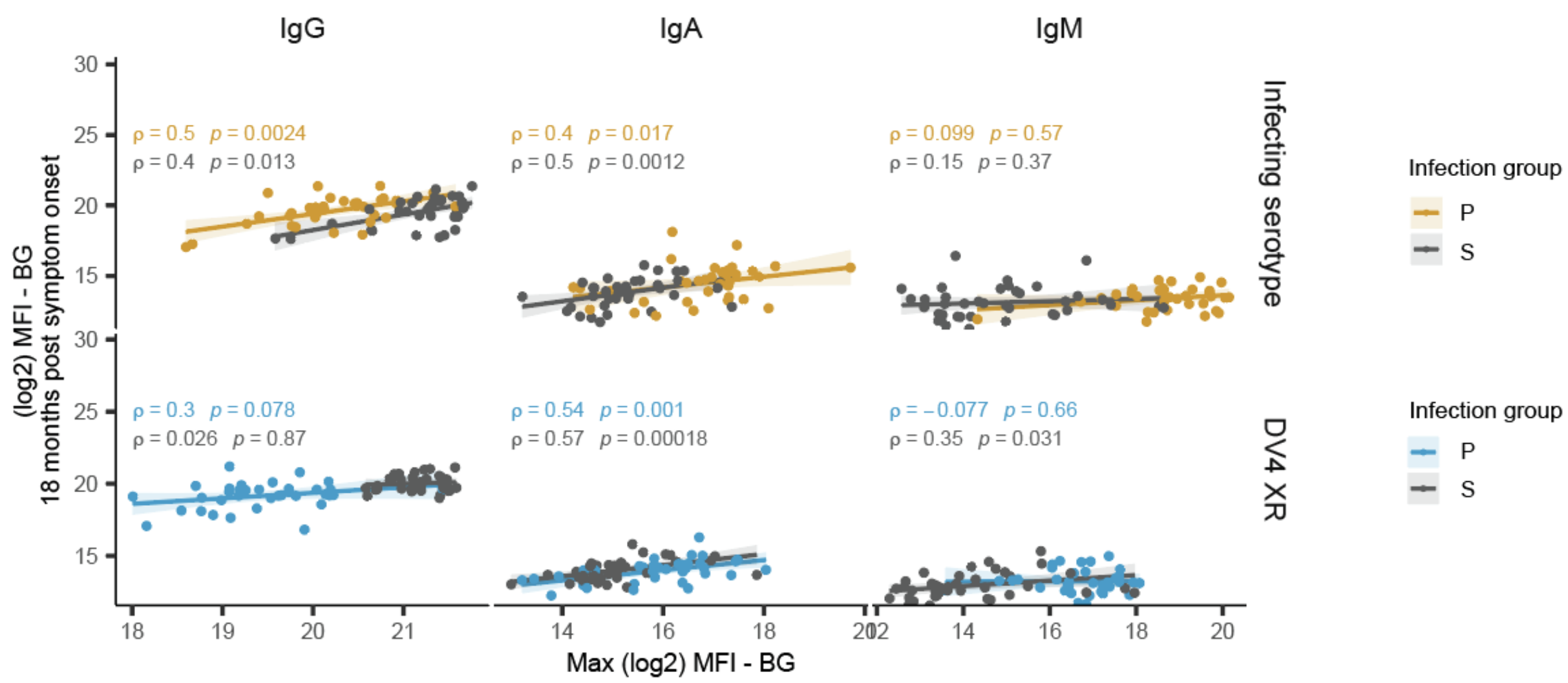

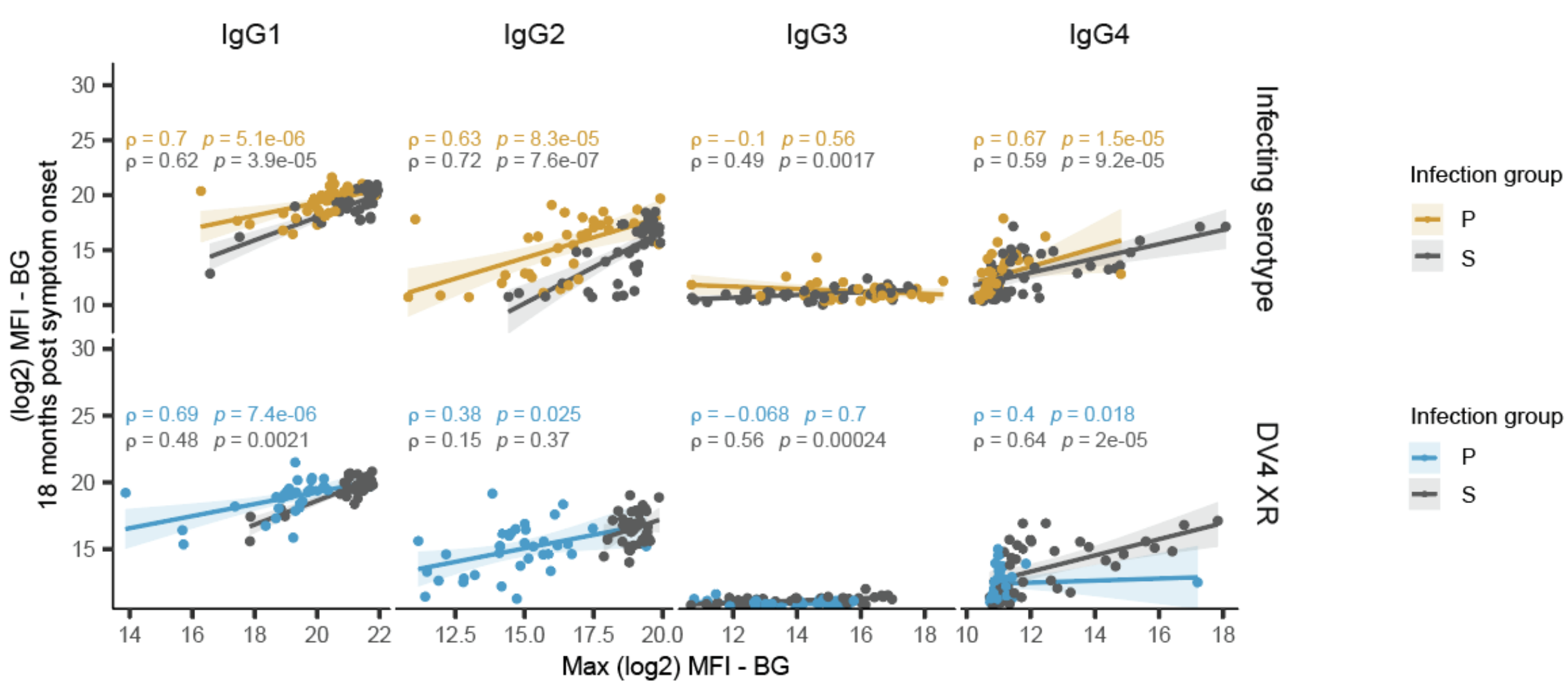

Supplementary Figure 11. High early antibody responses against E protein correlates with high antibody levels at 18 months post-infection for multiple antibody subsets. *E-binding plasma antibodies were measured as log_2 of mean fluorescence intensity (MFI) after background subtraction (BG). Post-primary responses against the infecting serotype are in yellow, and post-primary responses against cross-reactive DENV4 serotype are in blue (DV4 XR). All post-secondary antibody responses are in grey. The maximal observed level of DENV-binding antibody (i.e., Max) was defined for each individual from samples collected up to 3 months post-symptom onset. The magnitude of this maximal early DENV-binding antibody was*

correlated with the magnitude of the same antibody subset at 18 months post-symptom onset (Spearman ρ and p-values shown for each infection group). Each dot represents an individual. Axes were scaled to data for each antibody subset.

10. Line 133- Incorrect reference is made to a non-existent Figure 1F.

A: We thank the Reviewer for catching this error, which has now been corrected.

11. Lines 136-140- Reference to "levels" in this paragraph are particularly problematic; "responses" would be a more accurate description of the findings.

A: We have changed "levels" to "responses" as suggested by the Reviewer.

12. Lines 251-252- The statement "higher population seropositivity is associated with longer interepidemic periods and lower force of infection" is not an accurate description of the paper cited nor is it particularly relevant to the analysis described in this section.

A: We thank the Reviewer for this feedback. For clarification, we have removed this statement.

13. Lines 286-287- The authors should clarify that they are referring to the interval of 6-18 M.

A: We thank the Reviewer and have clarified this point.

14. Lines 292-293- "demonstrated this relationship more" should be revised to clarify whether the authors are referring to the slope or correlation coefficient.

A: This statement has been clarified to state "higher correlation coefficient."

15. Line 342- In-text citations are incorrectly formatted; "change in the antigenic composition over time" is confusing and should be revised for clarity.

A: We thank the Reviewer and have fixed this statement by removing the incorrectly placed citations.

16. Line 527- The following sentence needs further clarification- "Background-subtracted MFI values were calculated using average of naïve individuals + 3SD". The sentence should be clarified to state that "Background MFI values were calculated using average of naïve individuals + 3SD". Also, the authors should clarify whether these background values were log2 transformed before substraction (this is implied by line 526). It would be helpful to provide the range of background values, recognizing that there may be substantial variation between subsets.

A: We thank the Reviewer for this comment. Background MFI values were calculated as the mean signal among naïve individuals plus three standard deviations for each antigen–antibody combination. Log_2 transformation was applied prior to background correction; thus, values represent Log_2 signal relative to background (Log_2(MFI) − Log_2(background)). We have revised the Methods to clarify this procedure. A summary of the background value across antigen and isotype is provided as Source Data

File in the submission: "R2_AbDynamics_SourceData_LuminexBackground_summary.csv"

*Lines 529-532: "*Background MFI values were calculated as the mean signal among naïve individuals plus three standard deviations for each antigen–antibody combination. Log_2 transformation was applied prior to background correction; thus values represent Log_2 signal relative to background (Log_2(MFI) − Log_2(background)).*"*

17. Lines 588-589- "classified as significantly waning or rising when its 95% confidence interval excluded zero (i.e., infinite)" should be revised to "classified as significantly waning or rising when the 95% confidence interval of its decay rate excluded zero (i.e., infinite half-life)".

A: We thank the Reviewer for this helpful suggestion. The sentence has been revised accordingly.

Reviewer #3 (Remarks to the Author):

The authors provide an extensive rebuttal to the reviewers comments and have made significant changes to the manuscript. Statistical methodology and computational questions aside, the reviewer believes the authors have adequately addressed the comments and critiques.

The modifications in interpretation and representation of results have strengthened the paper and its accessibility to the non-dengue expert. The data are presented as novel but with a properly measured assessment of their generalizability and their limitations as it relates to understanding dengue pathogenesis.

The reviewer appreciates the long list of papers supporting the associations between first and second infections and immunologic profiles and dengue infection clinical outcomes. The reviewer's point is that association is not causation. Association can be a correlate if it meets statistical thresholds but even a correlate is not causation (unless a mechanistic correlate). The point was the importance of nuance in these discussions and not leaving the impression ADE is THE ONLY proposal. The modified language is very good and will appeal to a more general audience.

A: We thank the Reviewer for this thoughtful evaluation of the revised manuscript.
